# Genomic and functional characterization of *Bacillus* strains active against *Fusarium graminearum*

**DOI:** 10.3389/fmicb.2026.1832933

**Published:** 2026-05-29

**Authors:** Rim Touati, Mario Masiello, Isabella Pentimone, Stefania Somma, Miriam Haidukowski, Antonio Moretti, Palmira De Bellis

**Affiliations:** 1 Institute of Sciences of Food Production, Research National Council (ISPA-CNR), Bari, Italy; 2 Institute for Sustainable Plant Protection, National Research Council, Bari, Italy

**Keywords:** active metabolites, *Bacillus velezensis*, biocontrol agents, deoxynivalenol reduction, Fusarium Head Blight control, genome analysis

## Abstract

**Introduction:**

*Fusarium* species are among the most common and harmful fungal pathogens of cereals, able to synthesize a broad range of harmful mycotoxins, that they can accumulate in the kernels. The most important fungal disease is Fusarium Head Blight (FHB), caused by a complex of *Fusarium* species. *Fusarium graminearum* is the predominant species, able to produce deoxynivalenol (DON), a potent inhibitor of protein synthesis. The increasing demand for sustainable agriculture has driven research to find new eco-friendly strategies. This study aimed to identify new potential bacterial bio-control agents active against *F. graminearum*.

**Materials and methods:**

Thirty-six bacterial strains, belonging to *Bacillus velezensis*, *B. amyloliquefaciens*, *B. subtilis*, *B. licheniformis*, *B. mojavensis*, *B. simplex*, *B. oleronius*, *B. pumilus, B. safensis*, *Priestia megaterium*, and *Peribacillus simplex*, were selected. For each strain, antagonistic activity, by co-culture assay, and antifungal effect of bacteria filtrates were evaluated against five *F. graminearum* strains. All bacterial strains were molecularly characterized for the presence of genes associated with the production of antimicrobial molecules. In addition, ten selected strains were subjected to whole genome analyses.

**Results:**

Twenty-three strains exhibited antagonistic activity, achieving up to 75% mycelial growth inhibition. Furthermore, all *B. velezensis* strains, one strain of *B. amyloliquefaciens*, and *B. mojavensis* produced bioactive compounds that inhibited mycelial growth up to 64%. Co-culture assays demonstrated that all bacterial strains significantly reduced the ability of *F. graminearum* strains to produce DON up to 100%. Molecular analyses revealed the presence of genes encoding antimicrobial compounds in the majority of the bacterial strains. Genome analyses revealed the presence of a high number of secondary metabolite gene clusters (from 15 to 24), indicating a potential for secretion of multiple antimicrobial compounds. All strains shared genes devoted to the production of fengycin, surfactin, bacillibactin, mycosubtilin, bacillomycin D, bacillaene, and bacilysin, exhibiting, however, a distinct metabolite gene cluster profile. *Bacillus velezensis* strains presented the richest profiles, with N21.3 strain emerging as the most active against *F. graminearum*.

**Conclusion:**

This study provides new *Bacillus* strains with antifungal activity and genomic traits that make them promising candidates for sustainable management of FHB and mycotoxin contamination in wheat.

## 1 Introduction

Fungal diseases are among the most important factors influencing global crop production, since under environmental conditions suitable for fungal development, they can cause severe yield and quality losses. Among the phytopathogenic fungal genera, *Fusarium* is one of the most important, colonizing a broad range of plants, including cereals. Moreover, several *Fusarium* species not only represent a phytopathological issue but pose a serious toxicological risk for human and animal health for their ability to produce harmful mycotoxins. *Fusarium graminearum*, ranked fourth in the top ten of phytopathogenic fungi (Munkvold et al., 2021), is the most important pathogen of wheat, globally reported as the main causal agent of Fusarium Head Blight (FHB), a destructive small grain cereal disease. This species can colonize wheat plants from the early growing stages causing, together with a complex of other fungal species, Fusarium Crown Rot, seedling blight and seed decay (Ma et al., 2024; Guermech et al., 2025). *Fusarium graminearum* is the main producer of trichothecenes, potent inhibitors of protein synthesis, especially the type B trichothecenes deoxynivalenol (DON), nivalenol and their acetylated derivatives. Deoxynivalenol is highly toxic and poses serious health risks, causing skin irritations, hemorrhagic syndrome, feed rejection, and vomiting (Sobrova et al., 2010). The maximum levels of DON in unprocessed cereals and cereal based products are regulated by European Union (EU 2024/1022). In addition, DON has been proved to act as pathogenicity/virulence factor for infection of wheat heads thus playing a role in the pathogenesis of FHB (Bai et al., 2002; Proctor et al., 1995).

Due to the global phytopathological and toxicological concern of *F. graminearum* and other *Fusarium* species co-occurring on wheat, their control in field is crucial to guarantee safe final crop products.

In the last decades, the use of synthetic fungicides has been the main tool to control fungal plant pathogens. However, recently, the increased interest in environmental impact of chemicals and the attention posed to pesticide residues in foodstuff, addressed the researchers to develop new pest management tools. Among them, the use of microbial biocontrol agents (BCAs) is considered a promising eco-friendly strategy for integrated pest management programs. Within the BCAs, the most studied for their beneficial potential use in the agri-food supply chain are the lactic acid bacteria and different species belonging to *Bacillus*, *Pseudomonas* and *Streptomyces* genera (Daranas et al., 2019). In particular, *Bacillus* species play a key role as BCAs for their ability to reproduce actively in field and for their capability to overcome unfavorable environmental conditions (Ntushelo et al., 2019). Recently, some *Bacillus* species have been reported highly effective against a wide range of mycotoxigenic fungi, such as *Aspergillus flavus* (Einloft et al., 2021), the most important aflatoxin B1 producing species occurring on maize, and *F. graminearum* (Yuan et al., 2024). The activity of *B. siamensis* strains against *F. graminearum* and *F. pseudograminearum* was also reported by Zhang et al. (2022) and Dong et al. (2023), respectively.

The activity of BCAs is influenced by several factors including the interactions between the microbial agent, the target pathogen, the host plant and the environmental conditions. BCAs can act against the pathogen either by competing for space and nutrients or secreting antimicrobial compounds such as lipopeptides, bacteriocins, antibiotics, biosurfactants, cell wall degrading enzymes or microbial volatiles. In particular, *Bacillus* species have been reported as able to produce a wide range of active metabolites (Zhao and Kuipers, 2016; Tran et al., 2022; Wang et al., 2024), including subtilin, bacilysin, mycobacillin, bacillomycin, mycosubtilin, iturins, fengycins, and surfactins, all showing antifungal activities against pathogenic microorganisms (Assena et al., 2024; Adeniji et al., 2019; Khan et al., 2021).

Nowadays, few biocontrol agents have yet been registered as commercial products (https://food.ec.europa.eu/plants/pesticides/eu-pesticides-database_en, accessed on 15 February 2026). Therefore, it is necessary to deepen investigation to increase the number of commercially available microbial products for plant disease management and the knowledge of their mechanisms of action.

This study aimed to select new potential BCAs active against *F. graminearum*, and for this purpose, different *Bacillus* strains have been studied for their antagonistic and antifungal activities against five toxigenic *F. graminearum* strains and for their effect on DON production; the bacterial strains were molecularly characterized for the presence of key genes involved in the biosynthesis of active metabolites associated to antifungal activity; the whole genome of the strains selected as potential biocontrol agents was explored.

## 2 Materials and methods

### 2.1 Bacterial and fungal strains

Thirty-three bacterial strains, previously isolated from wheat grains and flours (De Bellis et al., 2015; Valerio et al., 2012), were used in this study. In particular, the strains belonged to 10 different species: nine strains of *B. velezensis*, four strains of *B. amyloliquefaciens*, five strains of *B. subtilis*, six strains of *B. licheniformis*, two strains of *B. mojavensis*, *B. pumilus* and *Peribacillus simplex,* and a single strain of *Priestia megaterium*, *B. oleronius*, and *B. safensis*. In addition, the strain *B. amyloliquefaciens* ATCC8473, previously identified as *B. subtilis* (ATCC-LGC Standards S.r.l., Sesto San Giovanni, MI, Italy), and two strains registered as BCAs on different crops, *B. velezensis* D747, previously reported as *B. amyloliquefaciens* subsp*. plantarum* D747 (Accession N. NRRL B-50405, NRRL Collection, Agricultural Research Service Culture Collection, Peoria, Illinois, United States) (European Commission, 2014), and *B. velezensis* QST713, synonymous of *B. subtilis* QST713 (Pandin et al., 2018), were also included in the study. All the strains, stored at −80 °C under cryoprotection, were cultivated on Tryptic Soy Agar (TSA, Biolife) at 30 °C for 2 days before each experiment.

The biological activity of each bacterial strain was evaluated against five *F. graminearum* strains, ITEM 633, ITEM 4577, ITEM 6415, ITEM 8311, ITEM 8318, isolated from wheat kernels and available in the ITEM microbial collection.^1^ They were grown on Potato Dextrose Agar (PDA, Difco) medium at 25 °C for 7 days before each experiment.

### 2.2 *In vitro* interaction studies

#### 2.2.1 Antagonistic activity against *Fusarium* strains

The antagonistic activity of the *Bacillus* strains against *F. graminearum* strains was evaluated by the dual culture method on solid nutrient media. Preliminary experiments were carried out to define the optimal conditions of co-cultivation, using the following media: Plate Count Agar (PCA, Difco), PDA and TSA. The latter was selected to perform in triplicates the experiments. In detail, a mycelial plug (4 mm in diameter) from the margin of 1-week-old colony was placed onto the center of TSA plate and two aliquots of 3 μL of a suspension in NaCl 0.85% of the *Bacillus* strain (10^9^ CFU/mL) was placed ca. 35 mm from the fungal plug on both sides. The plates inoculated only with each *Fusarium* strain were used as control. After 7 days of incubation at 25 °C, the orthogonal diameters of the fungal colonies were measured and used to evaluate the effect of bacterial strains on fungal growth. Inhibition values, expressed as percentage (I%), were calculated using the following formula:

I%=[(D−d)÷D]×100

where *D* was the diameter of the fungal colony used as control and *d* the radial growth of the fungal colony in dual culture tests (Haddoudi et al., 2021). Three independent experiments were carried out.

#### 2.2.2 Antifungal activity of *Bacillus* strains

All the *Bacillus* strains were evaluated for their capability to produce antimicrobial compounds active against *F. graminearum* strains in liquid medium. Preliminary tests were conducted to identify the optimal conditions for the active metabolites production. Three liquid media, Brain Heart Infusion (Oxoid) supplemented with 0.1% glucose (BHIG), Tryptic Soy Broth (TSB, Biolife) and PD3 (Davis et al., 1980; Zicca et al., 2020) were considered. *Bacillus* strains were 2% inoculated in 20 mL of broth and incubated under shaking (120 rpm) at 25 °C. After 24, 72, and 120 h of incubation, cell-free culture filtrates were obtained by centrifugation (13,000 rpm, 4 °C, 10 min) of the cultures and filtration of the supernatants through 0.22 μm filters. Based on preliminary test (data not shown), for the further analyses, PD3 broth and 72 h of incubation at 25 °C were selected as the best condition to obtain culture filtrates. The pH value of each filtrate and of the uninoculated medium was measured. The antifungal activity of the culture filtrates against *F. graminearum* strains was evaluated by agar well diffusion method. In detail, a mycelial plug (4 mm in diameter) from the margin of 1-week-old colony was placed onto the center of PDA plate and aliquots of 150 μL of culture filtrates were added in two wells (6 mm diameter) at ca. 35 mm from the fungal plug on both sides. The uninoculated medium was used as a control. After 7 days of incubation at 25 °C, the activity of culture filtrates on mycelial growth inhibition was calculated using the same formula above described. All experiments were performed in triplicate.

### 2.3 Detection of antimicrobial peptides genes

All *Bacillus* strains were screened for the presence of genes involved in the synthesis of active biomolecules, such as surfactin (*srfAA*), fengycin (*fenD*), iturin (*ituA*), bacillomycin (*bmyB*), bacilysin (*bacA*), difficidin (*dfnM*) and mycosubtilin (*mycA*). Bacterial DNA was extracted using the Wizard Genomic DNA Purification Kit (Promega, Madison, WI, United States) and amplified with primer pairs (Table 1) reported by Haddoudi et al. (2021), Mora et al. (2011), and Arguelles-Arias et al. (2009). The PCR reactions were carried out in a total volume of 15 μL containing 50 ng DNA template, 1.5 μL (10×) Hot Start buffer, 0.45 μL of each primer (10 mM), 1.2 μL of dNTPs (2.5 mM each) and 0.125 μL of Hot Start Taq DNA polymerase (5 U/μL; Fisher Molecular Biology). *srfAA*, *fenD*, *ituA*, *bacA*, *dfnM* and *mycA* gene fragments were amplified following the PCR parameters, 7 min at 95 °C, and 40 cycles of denaturation for 1 min at 95 °C, annealing for 1 min at 58 °C, elongation at 72 °C for 1 min and a final extension at 72 °C for 7 min. *bmyB* gene fragment was amplified following the PCR parameters, 7 min at 95 °C, and 35 cycles of denaturation for 1 min at 95 °C, annealing for 1 min at 55 °C, elongation at 72 °C for 1 min and a final extension at 72 °C for 7 min. The PCR products were verified by electrophoresis on a 1.5% agarose gel and visualized on a UV transilluminator.

**Table 1:** Primer pairs used for the screening of the genes involved in synthesis of antimicrobial peptides in *Bacillus* strains.

Gene	Primer name	Sequences of forward/reverse primers	PCR conditions			Reference
			Ta (°C)	N. of cycles	Expected amplicon size (bp)	
Surfactin (*srfAA*)	SRFAF SRFAR	5’-TCGGGACAGGAAGACATCAT-3’	58	40	201	Mora et al. (2011)
		5’-CCACTCAAACGGATAATCCTGA-3’				
Fengycin (*fenD*)	FENDF FENDR	5’-GGCCCGTTCTCTAAATCCAT-3’	58	40	269	
		5’-GTCATGCTGACGAGAGCAAA-3’				
Bacillomycin (*bmyB*)	BMYBF BMYBR	5’-GAATCCCGTTGTTCTCCAAA-3’	55	35	370	
		5’-GCGGGTATTGAATGCTTGTT-3’				
Bacilysin (*bacA*)	BACF BACR	5’-CAGCTCATGGGAATGCTTTT-3′ 5’-CTCGGTCCTGAAGGGACAAG-3′	58	40	498	
Mycosubtilin (*mycA*)	mycAF mycAR	5’-GCTGGACAAATTGCGGTTGT-3′	58	40	296	Haddoudi et al. (2021)
		5’-TGAGAAACAGCGGGCATCTT-3′				
Iturin (*ituA*)	ituAF ituAR	5’-TCGCCCTTCACTTTCAGCAT-3’	58	40	479	
		5’-TTAGCCGTCCTTCGCCATTT-3’				
Difficidin (*dfnM*)	dfnMF1 dfnMR1	5’-CGGAGTGAAACCGTGCCGGGATAAAGA-3′	58	40	1,250	Arguelles-Arias et al. (2009)
		5’ -GACCATTCAGAGCGGAAAGCTCC-3′				

Primer sequences, annealing temperature (Ta) and bibliographic reference have been reported for each gene.

### 2.4 Genome sequencing, assembly and annotation

Ten bacterial strains were selected for the whole genome sequencing among the three species that showed both antagonistic and antifungal activity, *B. velezensis, B. amyloliquefaciens,* and *B. mojavensis*. In particular, seven strains (*B. velezensis* N21.3, S106.1b, N3.2, N20.3 and S111.4, *B. amyloliquefaciens* ATCC 8473 and *B. mojavensis* S110.5) showed antimicrobial activity against *F. graminearum* while three (*B. amyloliquefaciens* S77.1 and N45.1, and *B. mojavensis* N67. B2), although showing a good antagonistic activity when cultured with *F. graminearum*, did not show any antifungal effect.

The total genomic DNA was extracted from overnight cultures grown in BHIG broth at 30 °C, using the Wizard® Genomic DNA Purification Kit (Promega, United States) according to manufacturer instructions. The genomes were sequenced using Illumina Novaseq 6,000 PE150 by an external service provider (Novogene Europe, Cambridge, United Kingdom). The taxonomic sequence classifier Kraken (Wood and Salzberg, 2014) was used for both detecting contaminants in datasets, eventually introduced during sample handling or sequencing, and asserting bacterial identifications. FastQC was employed for sequence quality control and reads quality trimming to the Q30 confidence level. Quality passed sequenced were assembled into draft genomes by CLC Genomics Workbench 25.0.1 (Qiagen Inc., Cambridge, MA), using default parameters. Genome Average Nucleotide Identity (ANI) values were evaluated to confirm the taxonomy of each strain.

Strains were evaluated by comparative genomics approach of carbohydrate active enzymes (CAZymes) and secondary metabolites profiles. Genomes of *B. velezensis* FZB42 and QST713 strains, both registered for the control of bacterial and fungal diseases, were retrieved from NCBI database and used for comparison. Secondary metabolites and CAZymes were searched into assembled draft genomes using antiSMASH 6.1.1 software (Blin et al., 2021) and dbCAN3 (Zheng et al., 2023) web server tools, respectively. For a more reliable CAZymes prediction, only proteins annotated according to three tools (HMMER, DIAMOND, dbCAN) were considered.

### 2.5 Safety prediction

The presence of virulence factor genes was investigated using the VFanalyzer tool from the Virulence Factor Database (VFDB), a comprehensive and curated online resource dedicated to bacterial virulence determinants.^2^

All genomes were also screened for the presence of antimicrobial resistance (AMR) genes with abriTAMR, an AMR gene identification pipeline that runs AMRFinderPlus organizing detected genes into functionally relevant groups (Horan et al., 2023). In addition, ABRicate was employed for large-scale screening of assembled contigs for antibiotic resistance determinants, using multiple reference databases, including ResFinder, CARD, and NCBI (Seemann, 2016).

PlasmidFinder tool was used to detect plasmid-associated sequences, since plasmids can facilitate the dissemination of antibiotic resistance genes, virulence factors, and other adaptive traits across bacterial populations (Carattoli and Hasman, 2019).

### 2.6 Deoxynivalenol production in presence of bacterial strains

To evaluate the capability of bacterial strains to influence mycotoxin production, each strain was co-cultivated with *F. graminearum* ITEM 8318 strain, selected for its great capability to produce DON in experimental conditions. Co-cultivation was performed into 250 mL Erlenmeyer Flasks containing 50 g of rice imbibed overnight with 30 mL (approx. 60% v/w) distilled water and then sterilized at 121 °C for 30 min. Each flask was inoculated with a 1 mL of fungal conidial suspension (10^6^ spores/ml) and 1 mL of *Bacillus* strain suspension (10^6^ CFU/mL) and incubated for 21 days at 25 °C, in darkness. Then, the inoculated kernels were dried at 55 °C for 48 h and finely milled for further analyses.

DON was determined by HPLC-DAD at 220 nm following a PDA-based chromatographic approach supported by the validated method of D’Ascanio et al. (2026) for cereal matrices, including rice and wheat. Briefly, five grams of dried milled rice culture were extracted with 15 mL of acetonitrile/ water (84:16, v/v) with 1% of acetic acid by orbital shaking for 1 h. The extract was diluted with water (50:50, v/v) and directly injected into HPLC apparatus (Agilent 1,260 Series). The column was set at 40 °C and a diode array (DAD) detector at 220 nm wavelength. The analytical column was a Synergi Hydro-RP 80A (150 × 3 mm, 4 μm, Phenomenex). The mobile phase consisted of a mixture of acetonitrile/ water (10:90, v/v) elute at a flow rate of 0.5 mL/min. DON was quantified by measuring peak areas at the retention time of DON standard solutions and comparing them with the relevant calibration curve from 20 to 2,500 ng/mL. Deoxynivalenol standard (purity > 99%) was supplied by Sigma-Aldrich (Milan, Italy). All solvents (HPLC grade) were purchased from J. T. Baker (Deventer, Netherlands). Water was of Milli-Q quality (Millipore, Bedford, MA, United States). Calibrant solutions for standard calibration curves were prepared by drying different aliquots of the DON stock solution (1 mg/mL in acetonitrile) and successively reconstituted in the HPLC mobile phase. Under these experimental conditions, retention time was 5.9 min and the detection limit was 0.04 μg/g.

Moreover, after incubation the presence of fungal and each bacterial strain was verified. Ten grams of inoculated rice were diluted with 90 mL of sterile NaCl solution (0.85%, w/v) and homogenized in a Stomacher (Seward, London, United Kingdom) for 2 min. After serial dilutions, the microbial suspensions were plated on an Plate Count Agar (PCA, Difco, Franklin Lakes, NJ, United States) supplemented with 100 mg/L of cycloheximide (EMD Millipore Corp., Billerica, MA, United States) incubated at 30 °C for 48 h for the determination of Bacillus strains, and on a Potato Dextrose Agar (PDA, Difco, Franklin Lakes, NJ, United States) supplemented with 200 mg/L chloramphenicol (Sigma, Milan, Italy) incubated at 25 °C for 72 h for the enumeration of the fungal strain.

### 2.7 Statistical analyses

The results of antagonistic and antifungal activities were presented as mean values ± standard deviations. Data were subject to statistical analysis using Statistica 12.0 software (StatSoft, Inc., Tulsa, OK, United States). Data concerning antifungal activity were compared using one-way ANOVA followed by Tukey’s test to determine significant differences (*p* < 0.05).

## 3 Results

### 3.1 Effects of the *Bacillus* strains on fungal growth

**Figure 1:**
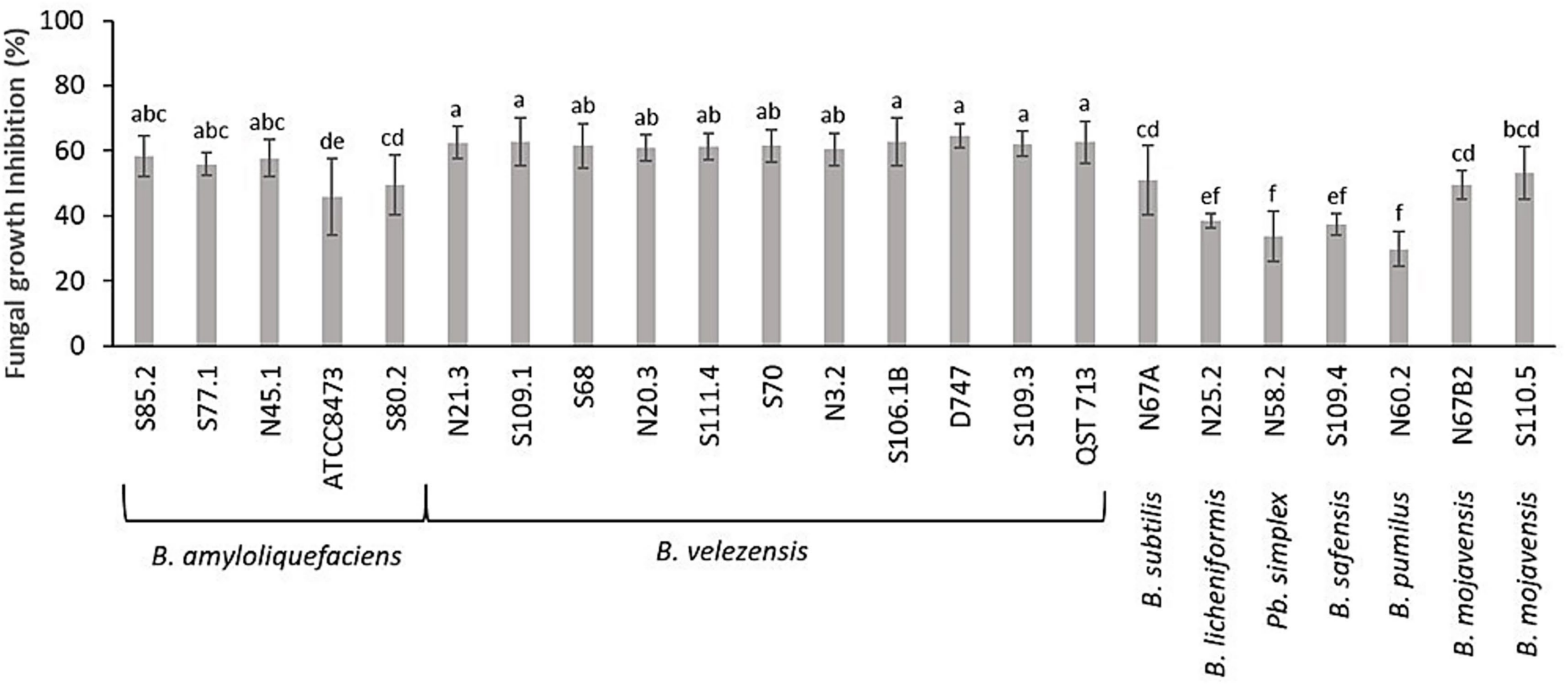
Antagonistic activity of *Bacillus* strains against five *F. graminearum* strains on TSA, after 7 days of incubation at 25 °C. The mean value of fungal growth inhibition of five *F. graminearum* strains was expressed as percentage. Different letters indicate significant differences (*p* < 0.05) among the tested *Bacillus* strains, determined by one-way analysis of variance (ANOVA) followed by the Tukey test.

**Figure 2:**
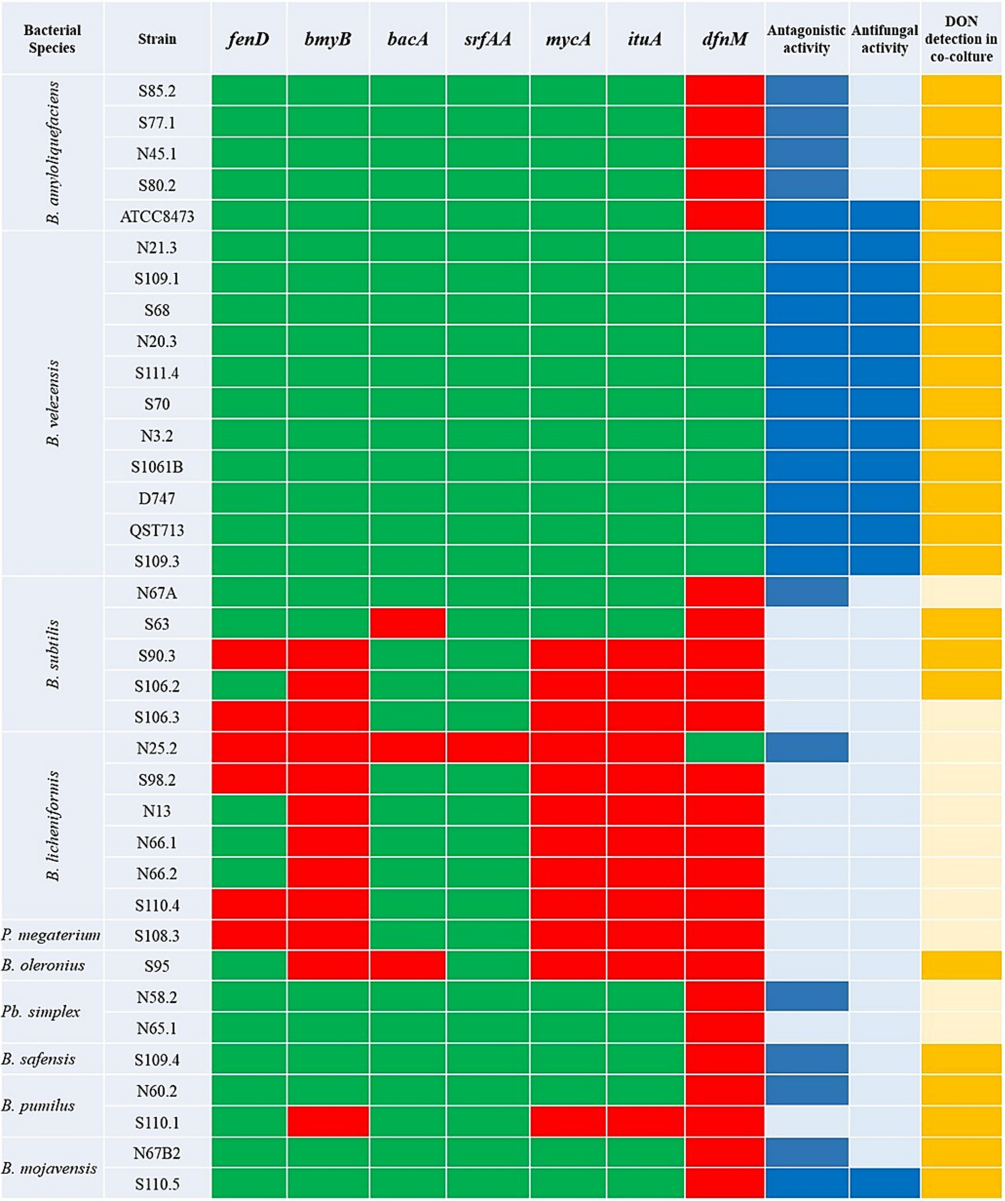
Molecular characterization of 36 bacterial strains for the presence of genes involved in the synthesis of antimicrobial compounds (*fenD:* fengycin, *bmyB:* bacillomycin, *bacA:* bacilysin, *srfAA*: surfactin, *mycA:* mycosubtilin*, ituA:* iturin, and *dfnM:* difficidin). Antagonistic and antifungal activities, and DON detection in co-culture assays are also reported. Color keys correspond to presence (green) or absence (red) of the genes; presence (blue) or absence (light blue) of antagonistic/antifungal activities; absence (orange) or presence of DON (light orange) detected on rice co-cultures.

Twenty-three out of 36 *Bacillus* strains showed antagonistic activity against the five tested *F. graminearum* strains, although variable fungal growth inhibition values were observed (Supplementary Figure 1). Among the bacterial species, *B. velezensis*, *B. amyloliquefaciens* and *B. mojavensis* strains were the most active, inhibiting mycelial growth up to 75, 68 and 60%, respectively (Supplementary Figure 1). In particular, all *B. velezensis* strains showed a similar capability to inhibit fungal growth, with mean inhibition values ranging between 61 and 65%. On the other hand, a greater variability was observed among *B. amyloliquefaciens* and *B. mojavensis* strains (mean inhibition values ranging from 45 to 58%; Figure 1). Among the tested *B. amyloliquefaciens* strains, S85.2, S77.1 and N45.1 strains were the most effective against *F. graminearum*, inhibiting mycelial growth of all fungal strains with values higher than 50% (Figure 1). With regard to the other *Bacillus* species, only a single *B. subtilis* strain (N67A) and one *B. licheniformis* strain (N25.2) showed antagonistic activity, with mean inhibition values of 50.9 and 38.5%, respectively (Figure 1). Antagonistic activity, with mean inhibition values of 37.4, 29.8, and 33.7% was observed for the *B. safensis* strain S109.4, *B. pumilus* N60.2, and *Pb. simplex* N58.2, respectively (Figure 1). Conversely, *P. megaterium* S108.3 and *B. oleronius* S95 strains, four *B. subtilis* strains and five *B. licheniformis* strains showed no antagonistic activity against the five tested *F. graminearum* strains (Figure 2).

All fungal strains were inhibited by the 23 active *Bacillus* strains, although a slight variability, in terms of mycelial growth, was observed among them (Supplementary Figure 1). In particular, the growth of *F. graminearum* ITEM6415 strain was reduced by all *B. velezensis* strains with inhibition values ranging from 67% (N20.3 and S111.4 strains) to 75% (S109.1 strain).

### 3.2 Antifungal activity of *Bacillus* strains

The antifungal activity varied depending on the growth medium used, suggesting that medium composition influenced metabolite production. PD3 medium for 72 h of incubation at 25 °C were the conditions selected for antimicrobial activity assays. After 72 h of incubation, a slight pH difference, ranging from 6.28 to 7.00, in the 36 filtrates compared to uninoculated medium (pH 6.9) was observed, indicating that the antifungal activity of the *Bacillus* strains was not attributable to pH variations.

**Figure 3:**
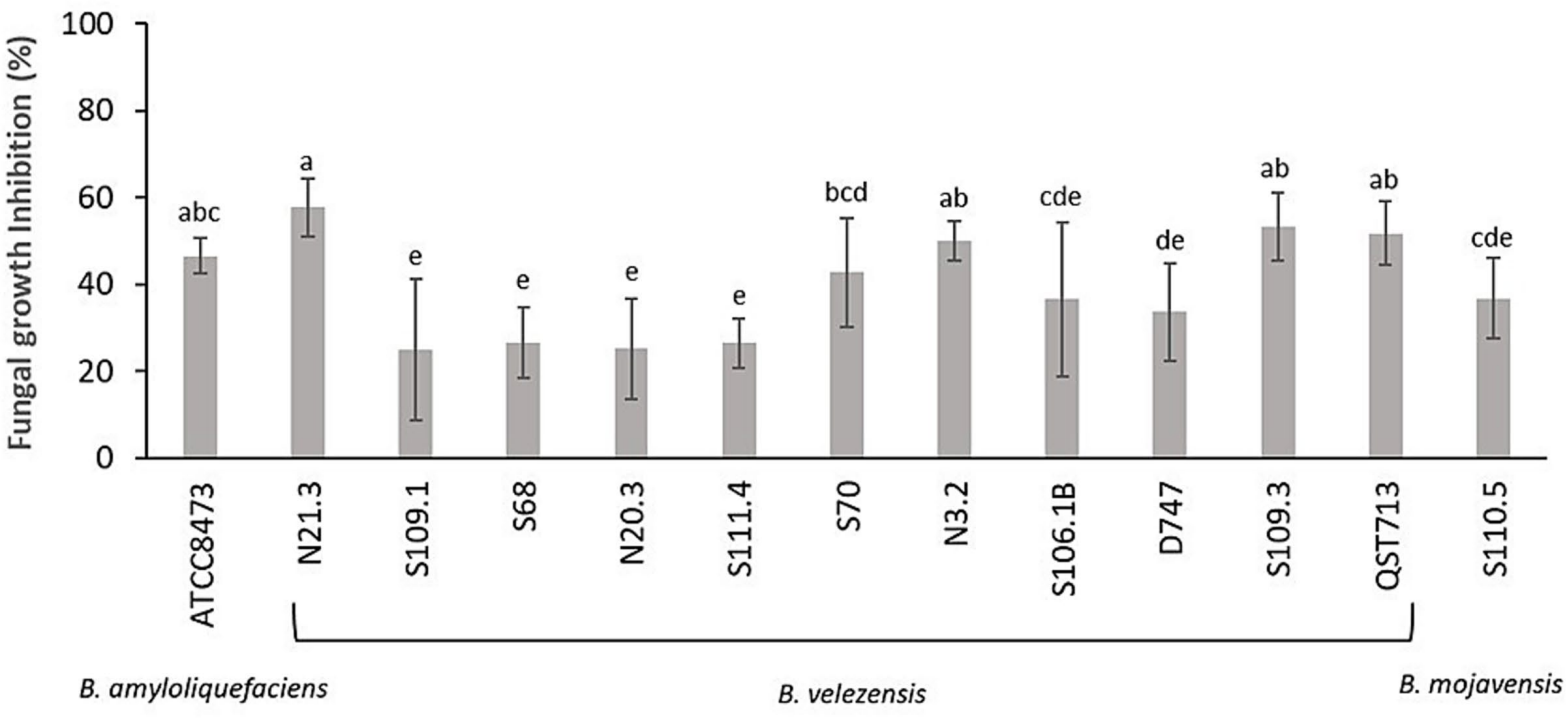
Antifungal activity of *Bacillus* culture filtrates against five *F. graminearum* strains on PDA, after 7 days of incubation at 25 °C. The mean value of fungal growth inhibition of five *F. graminearum* strains was expressed as percentage. Different letters indicate significant differences (*p* < 0.05) among the tested *Bacillus* strains, determined by one-way analysis of variance (ANOVA) followed by the Tukey test.

The results of the antifungal activity assays are shown in Figure 3 and Supplementary Figures 2, 3. Among the 36 *Bacillus* strains, those that did not exhibit antagonistic activity *in vitro* also failed to produce antimicrobial compounds when cultured in PD3 liquid medium. Therefore, among the 23 strains showing antagonistic activity (Figure 1), only 13 of them produced bioactive filtrates (*B. amyloliquefaciens* ATCC 8473; *B. velezensis* N21.3, S109.1, S68, N20.3, S111.4, S70, N3.2, S106.1B, S109.3, D747 and QST713; *B. mojavensis* S110.5; Figure 3).

All filtrates of *B. velezensis* strains were active against the five *F. graminearum* strains, with inhibition values up to 64% (Supplementary Figure 2). The most effective strains were *B. velezensis* N21.3, N3.2, S109.3 and QST713, while the strains S109.1, S68, N20.3, and S111.4 exhibited lowest mean antifungal activity (Figure 3). In addition, antifungal effects were observed for *B. amyloliquefaciens* ATCC 8473 strain and *B. mojavensis* S110.5 strain, with mean inhibition values of 46 and 37%, respectively. No antifungal activity was observed for the other *B. amyloliquefaciens* strains. Furthermore, several strains that had shown antagonistic activity *in vitro*—namely *B. subtilis* N67A, *B. licheniformis* N25.2, *B. simplex* N58.2, *B. safensis* S109.4, *B. pumilus* N60.2, and *B. mojavensis* N67B2—did not produce bioactive compounds (Figure 2).

### 3.3 Molecular identification of genes involved in antimicrobial compounds production

All 36 bacterial strains were screened for the presence of seven key genes involved in the synthesis of known bioactive compounds (Figure 2; Supplementary Figure 4). All screened genes were detected in all *B. velezensis* strains, while a different profile was observed in all other strains of analyzed species. Indeed, PCR amplifications revealed that among all strains analyzed that were not belonging to *B. velezensis*, the occurrence of genes varied from 2 to 6 genes detected. In particular, all bacterial strains, except for *B. licheniformis* N25.2, and *B. subtilis* S63 and *B. oleronius* S95, had the *srfAA* and *bacA* genes. *FendD* gene was detected in the majority of the strains (83%), being amplified in all *B. amyloliquefaciens, B. oleronius, B. safensis, B. pumilus,* and *B. mojavensis* strains. A different response was observed in *B. subtilis* and *B. licheniformis* strains, since some of them failed *fendD* PCR amplification. Moreover, all *B. licheniformis* strains, *B. subtilis* S90.3, S106.2, S106.3, *P. megaterium* S108.3, *B. oleronius* S95 and *B. pumilus* S110.1 lacked the *bmyB, mycA* and *ituA* genes (Figure 2; Supplementary Figure 4).

### 3.4 Genome sequence

For a more in-depth characterization, seven strains that produced active filtrates and three strains that did not produce them, were subjected to whole genome analysis. An average of approximately 10 million good quality reads (150 bp Illumina paired reads) were obtained for each strain and used for genome assembling. A summary of the results is provided in Supplementary Table 1. Although fragmented, all draft genomes were compatible with corresponding genome of the reference strains for dimension and ANI values very high for each strain (data not shown).

#### 3.4.1 Genetic basis for the anti-pathogen activity

Assembled draft genomes were analyzed, also with comparative genomics approaches, to identify bioactive molecules involved in fungal growth inhibition.

All genomes analyzed harbored multiple secondary metabolites gene clusters. Several clusters showed high similarity with well-known biosynthetic gene clusters involved in antimicrobial activity, plant immunity, resistance towards pathogenesis, plant growth promotion and biofilm formation (Table 2). Other putative gene clusters involved in the synthesis of antimicrobial compounds, including Non-Ribosomal Peptide Synthetase (NRPS), ribosomally synthesized and post-translationally modified peptide product (RiPP), lanthipeptide-class-iii, Type III Polyketide synthase (T3PKS), were also identified (Supplementary Table 2).

**Table 2:** Secondary metabolites identified into *Bacillus* strains genomes.

Known cluster	*B. velezensis*					*B. amyloliquefaciens*			*B. mojavensis*	
	N21.3	S106.1b	N3.2	N20.3	S111.4	S77.1	N45.1	ATCC8473	N67B2	S110.5
Fenycin	√	√	√	√	√	√	√	√	√	√
Surfactin	√	√	√	√	√	√	√	√	√	√
Bacillibactin	√	√	√	√	√	√	√	√	√	√
Mycosubtilin	√	√	√	√	√	√	√	√	√	√
Bacillomycin D	√	√	√	√	√	√	√	√	√	√
Paenilarvins	√	√	√	√	√	√	√	√		
Iturin	√	√	√	√	√	√	√	√		
Plipastatin	√	√		√					√	√
Paenibactin									√	√
Bacillaene	√	√	√	√	√	√	√	√	√	√
Difficidin	√	√	√	√	√					
Macrolactin H	√	√	√	√	√					
Macrobrevin				√					√	
Aurantinin B/C/D				√					√	
Bryostatin				√						
Mersacidin					√					
Amylocyclina			√			√	√	√		
Sorangicin A									√	
Bacilysin	√	√	√	√	√	√	√	√	√	√
Butirosin A/B	√	√	√	√	√	√	√	√		
Rhizocticin A	√									
Thailanstatin A									√	√
Ericin S	√									
Plantazolicin		√								
Subtilin			√							
Subtilosin A									√	√
Subtilomycin										√
Known cluster	15	14	14	16	13	11	11	11	14	12
Unknown cluster	9	5	5	4	6	6	6	6	3	3
Total clusters	**24**	**19**	**19**	**20**	**19**	**17**	**17**	**17**	**17**	**15**

Known clusters are colored according to classification type: Non-Ribosomal LipoPeptide (green); Ribosomal Peptide (pink), Polyketides (blue); other types (white).

In particular, secondary metabolite gene cluster profiles showed few differences among analyzed strains belonging to the same species. All strains were presumably capable of secreting fengycin, surfactin, bacillibactin, mycosubtilin, bacillomycin D, bacillaene, and bacilysin (Table 2).

In addition, *B. velezensis* strains also harbored gene clusters for difficidin, macrolactin, iturin, butirosin, and paenilarvins, metabolites with antimicrobial activity, while plipastatin cluster was predicted only in N20.3, N21.3 and S106.1b strains of the same species. Other clusters detected result as single strain features, including those related to metabolites with a narrow spectrum of antibiotic action, such as nematicidal plantazolicin (S106.1b) or bactericidal subtilin and amylocyclin (N3.2), ericin and rhizocticin (N21.3), mersacidin (S111.4) or macrobrevin, aurantinin and bryostatin (N20.3). As to concern products with no sequence similarity to notorious bacterial gene clusters, terpene and T3PKS types were detected in all *B. velezensis*, as well as in all other *Bacillus* strains (Supplementary Table 2). Phosphonate type was present in genome of *B. velezensis* N3.2, N20.3 and S111.4 strains, while the laddarene cluster was observed in S106.1b and the LAP-thiopeptide cluster in strain N3.2. Non-Ribosomal Peptide Synthetase class was also widely detected in strain N21.3, containing as many as six gene clusters, whereas strain S111.4 harbored two clusters of this class. Among all strains, *B. velezensis* exhibited the highest biosynthetic capacity for secondary metabolites production. In particular, *B. velezensis* N21.3 strain, with its 24 secondary metabolite clusters, accounting for a considerable fraction (20%) of the genome size, was the most endowed.

As to concern *B. mojavensis,* in addition to the common above-mentioned gene clusters, both analyzed strains could produce the secondary metabolites plipastatin, paenibactin, subtilosin and thailanstatin. Strain S110.5 could predictively secrete also subtilomycin, while N67B2 strain could produce aurantin, sorangin and macrobrevin.

With regards to *B. amyloliquefaciens*, the three strains analyzed shared an identical profile, having 11 gene clusters showing high similarity to the well-known clusters, and 6 gene clusters, belonging to metabolite type terpene, lanthipeptide-class-iii, T3PKS, NRPS and NRPS-like, showing no similarity to already known bacterial gene clusters.

#### 3.4.2 Carbohydrate active enzymes and prediction

Strains were analyzed for their CAZyme repertoire, as these enzymes influence the nature and efficiency of carbohydrate utilization, playing a key role in shaping bacterial interactions with fungi and plants, particularly in terms of antagonistic behavior.

**Figure 4:**
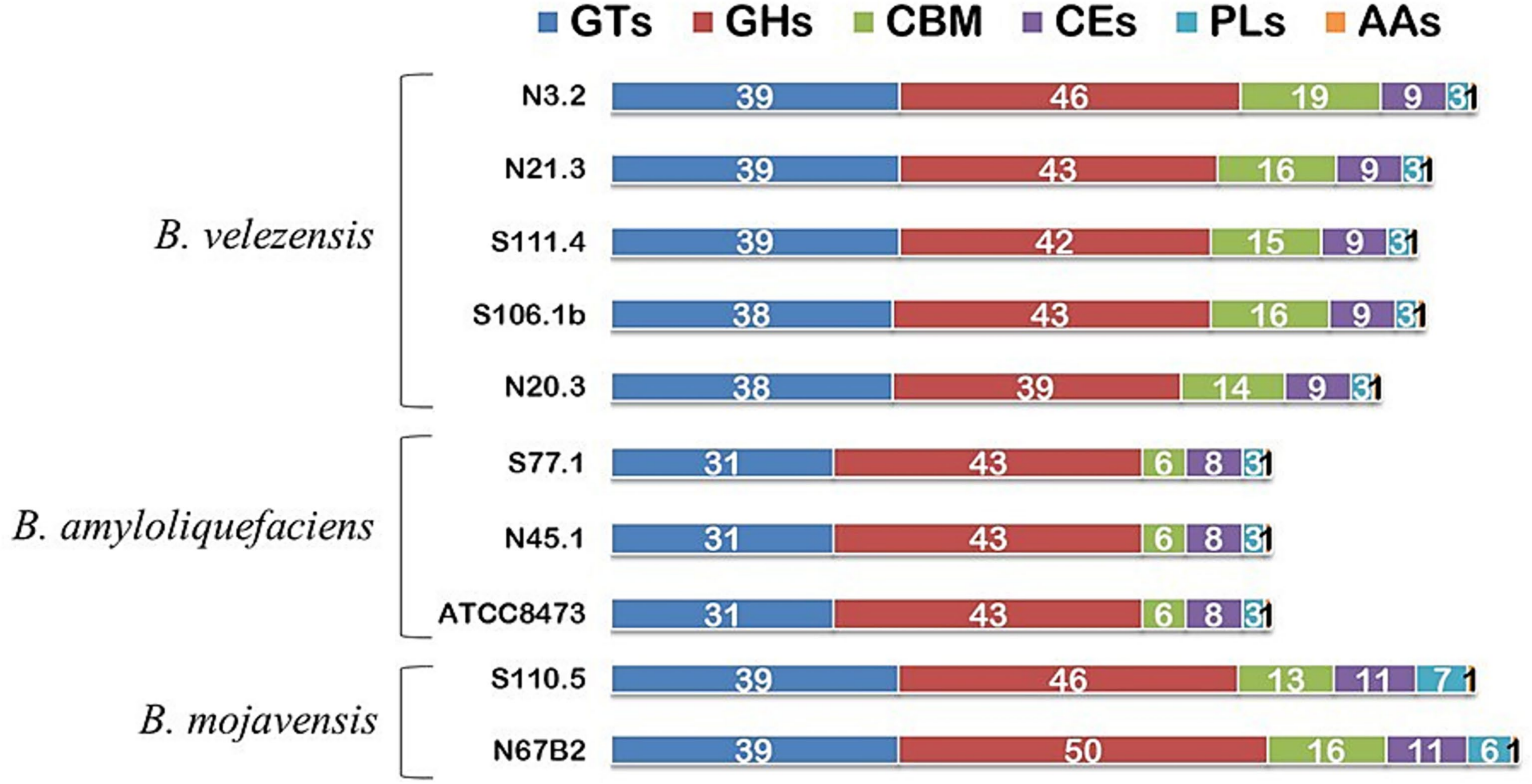
CAZyme distribution in the genomes *of B. velezensis, B. mojavensis* and *B. amyloliquefaciens* strains. GTs, glycosyltransferases; GHs, glycoside hydrolases; CBM, carbohydrate-binding modules; AAs, auxiliary activities; CEs, carbohydrate esterase; PLs, polysaccharide lyases.

All *B. velezensis* and *B. mojavensis* strains shared a similar enzymes panel, including glycosyl-transferases (GTs), polysaccharide lyases (PLs), polysaccharide esterases (CEs), and auxiliary activity family (AAs) (Figure 4). Within these two species, differences among the strains regarded the consistency of glycoside hydrolase (GH), from 38 to 46 members for *B. velezensis* and from 46 to 50 members for *B. mojavensis*. Similarly, carbohydrate binding modules (CBMs) varied from 14 to 19 members for *B. velezensis* and from 13 to 16 members for *B. mojavensis* (Figure 4).

CAZyme analysis of *B. amyloliquefaciens* revealed identical assortment among strains, specifically 43 GH members, 31 GTs, 6 CBMs, 3 PLs, 8 CEs, and 1AAs (Figure 4).

Hydrolytic enzymes such as chitinases, chitosanases, glucanases, cellulases, lipases, and proteases, were also extensively predicted in all *Bacillus* strains analyzed. These compounds efficiently hydrolyze the major components of the fungal and bacterial cell walls, leading to plant pathogen inhibition. All strains had genes coding for chitinase (i.e., GH18, GH23, GH73 and GH171), pectinase such as poligalatturonase (i.e., GH28) and pectate lyase (PL1, PL9), all involved in antimicrobial activity (Supplementary Table 3).

#### 3.4.3 Safety and regulatory assessment

Strain identification was supported by Average Nucleotide Identity (ANI) analysis, defined as the mean nucleotide identity of shared orthologous gene pairs between two microbial genomes, in combination with Kraken-based taxonomic classification. These complementary approaches enabled accurate taxonomic assignment and confirmed that the analyzed strains do not belong to known pathogenic groups, thereby mitigating potential biosafety concerns. Analysis using VFDB indicated that the number of genes putatively associated with virulence factors was limited, about 40 genes out of the total number of genes (Supplementary Table 4). Closer inspection revealed that many of these genes encode core biological functions, primarily involved in environmental stress response, nutrient acquisition, or general fitness mechanisms rather than traits directly associated with pathogenicity (Supplementary Table 5). No canonical virulence determinants associated with host invasion, toxin production, or immune evasion were detected.

Although a limited number of antimicrobial resistance genes were detected, these were primarily intrinsic, chromosomally encoded, and not associated with mobile genetic elements or clinically relevant resistance phenotypes. Therefore, their presence does not compromise the biosafety profile of the strains. Moreover, plasmid analysis revealed that none of the analyzed strains harbored plasmids that could carry worrisome virulence associated genes, further reducing concerns related to horizontal gene transfer of pathogenic traits (Supplementary Table 4).

### 3.5 Effect of *Bacillus* strains on the deoxynivalenol production

The effect of 36 *Bacillus* strains on DON production was evaluated *in vitro* condition, co-culturing each bacterial strain with the high DON producer *F. graminearum* strain ITEM 8318 (Table 3; Figure 2). After 21 days of incubation, ITEM 8318 grown alone on rice (positive control) produced the highest amount of DON (61.2 mg Kg^−1^). In contrast, in all co-cultures DON was either undetected (detection limit < 0.04 μg·g^−1^) or markedly reduced. Specifically, in 69% of the samples, DON was not detected, while in the remaining cases the production was drastically reduced, ranging from 0.4 mg·kg^−1^ (*B. subtilis* S106.3) to 18 mg·kg^−1^ (*B. simplex* N58.2).

**Table 3:** Effect of microbial co-inoculation on bacterial and fungal counts (log CFU/g), and DON production after 21 days of incubation at 25 °C.

Species	Bacterial strain	Bacterial counts (log CFU/g)	Fungal counts (log CFU/g)	DON (mg/kg)	DON reduction (%)
Control (ITEM8318)		–	6.53	61.2	0
*B. amyloliquefaciens*	S85.2	10.24	nd	nd	100
	S77.1	7.17	nd	nd	100
	N45.1	7.69	nd	nd	100
	S80.2	7.7	nd	nd	100
	ATCC8473	7.84	nd	nd	100
*B. velezensis*	N21.3	8.69	nd	nd	100
	S109.1	8.54	nd	nd	100
	S68	8.96	nd	nd	100
	N20.3	8.3	nd	nd	100
	S111.4	8.544	nd	nd	100
	S70	9.13	nd	nd	100
	N3.2	8.94	nd	nd	100
	S1061B	8.63	nd	nd	100
	D747	8.94	nd	nd	100
	QST713	8.59	nd	nd	100
	S109.3	8.7	nd	nd	100
*B. subtilis*	N67A	9.13	2.48	4.9	92
	S63	5.48	5.48	nd	100
	S90.3	7.6	nd	nd	100
	S106.2	8.08	nd	nd	100
	S106.3	7.08	5.48	0.4	99.4
*B. licheniformis*	N25.2	7.01	5.15	0.4	99.3
	S98.2	6.3	7.45	2.7	95.6
	N13	5.6	5.58	0.2	99.7
	N66.1	8.36	6.08	0.2	99.7
	N66.2	4.9	5.6	2.4	96.1
	S110.4	7.45	6.5	5.8	90.5
*P. megaterium*	S108.3	5.36	5.78	1.7	97.2
*B. oleronius*	S95	5.48	6.11	nd	100
*Pb. simplex*	N58.2	4.23	6.49	18	70.6
	N65.1	8.48	6.04	7.2	88.2
*B. safensis*	S109.4	9.59	nd	nd	100
*B. pumilus*	N60.2	8.37	6.36	nd	100
	S110.1	8.12	7.3	nd	100
*B. mojavensis*	N67B2	8.74	3.3	nd	100
	S110.5	9	4.28	nd	100

*B*., *Bacillus*; *P*., *Priestia*; *Pb*., *Peribacillus.*

Additionally, after incubation, the presence of both bacteria and *F. graminearum* was evaluated in the inoculated rice samples. High bacterial densities were observed, ranging from 4.9 log CFU/g (*B. licheniformis* N66.2) to 10.24 log CFU/g (*B. amyloliquefaciens* S85.2; Table 3). Conversely, fungal growth, with few exceptions, was either undetected or consistently lower than in the control samples. Notably, the fungal strain and DON were absent in all samples containing *B. amyloliquefaciens* and *B. velezensis* strains, as well as in two out of five *B. subtilis* strains and the single *B. safensis* strain, suggesting a direct inhibition of fungal growth; while in samples containing *B. subtilis* (S63), *B. oleronius*, or any *B. pumilus* and *B. mojavensis*, the fungus was present, but the DON was not detected. In these cases, the bacteria may have interfered with fungal metabolic activity or degraded the toxin.

## 4 Discussion

*Fusarium graminearum* is one of the most important fungal pathogens of cereals worldwide, representing both a phytopathological issue and a great concern for human health for its ability to produce DON. In recent years, species of the genus *Bacillus* have emerged as suitable BCAs for their great ability to inhibit fungal growth and the strong resilience under adverse environmental conditions (Ntushelo et al., 2019). Previous studies reported the biological activity of *Bacillus* strains against several *Fusarium* species, including *F. graminearum* (Gong et al., 2015; Zalila-Kolsi et al., 2016; Fan et al., 2018), *F. verticillioides*, the most important pathogen of maize (Yuan et al., 2024), *F. equiseti* and *F. poae*, weak pathogens occurring on a wide range of different crops (Haddoudi et al., 2021; Zanon et al., 2024). To broaden the range of candidates for biological control, this study explored the activity of 36 bacterial strains belonging to different *Bacillus* species, against *F. graminearum* strains. Twenty-three bacterial strains exhibited antagonistic activity, significantly inhibiting mycelial growth. Bacterial strains showing antagonistic activity belonged to *B. velezensis*, *B. amyloliquefaciens*, *B. subtilis*, *B. licheniformis*, *B. mojavensis*, *B. pumilus*, *B. safensis* and *B. simplex* species. However, only *B. velezensis* strains, and a strain each of *B. amyloliquefaciens* and *B. mojavensis* produced extracellular metabolites, showing both antagonistic and antifungal activities, simultaneously. Our results corroborate that inhibitory mechanisms differ among strains and could be influenced by the substrate composition and growth conditions (Wang et al., 2024; Mizumoto and Shoda, 2007).

All *B. velezensis* strains were the most effective showing both antagonistic and antimicrobial activities, as observed by other authors. Zanon et al. (2024) selected a *B. velezensis* strain as a potential candidate to control the development of *F. graminearum* and *F. poae* in barley, while Zhang et al. (2026) reported the activity of the same species against *F. graminearum, Alternaria alternata* and *A. flavus*. Furthermore, the potential importance of this bacterial species as BCAs is strengthened by the recent taxonomic revisions, since several strains marketed as BCAs were reidentified as *B. velezensis* (Pandin et al., 2018; Dunlap, 2019).

The potential capability of synthesizing antimicrobial metabolites was assessed by detecting key associated genes. In all *B. velezensis* strains, genes encoding for surfactin, fengycin, iturin, bacillomycin, bacilysin, difficidin and mycosubtilin were detected, while in the strains belonging to the other species only from two to six genes were detected. This agrees with previous reports, confirming that some bacterial species are particularly enriched in genes linked to antimicrobial activity and that notable differences exist among and within species. For instance, Gond et al. (2015) reported the presence of fengycin and iturin genes in the genomes of *Bacillus* species. A *B. subtilis* strain, with antagonistic activity against *F. graminearum,* showed the presence of five antimicrobial peptide genes, including *bmyB*, *fenD*, *ituC*, *srfAA* and *bacA* (Zhao et al., 2014). In addition, more than 50% of the *B. amyloliquefaciens*, *B. velezensis*, *B. subtilis* and *B. mojavensis* strains analyzed by Haddoudi et al. (2021) simultaneously harbored five to seven genes encoding for bacillomycin B, fengycin D, surfactin A, subtilin S, difficidin M, mycosubtilin A and iturin A.

Antagonistic and antifungal assays, combined with PCR screening, highlighted the most promising potential candidates for biological control. Nowadays, genomic insights represent a crucial step in the selection of BCAs, since they provide relevant information for the identification of key traits involved in biocontrol activity linked to the synthesis of antimicrobial compounds. Comparative genomics also offer a framework for future functional studies, such as evaluations of environmental fitness and biocontrol efficacy. To gain deeper insights, whole-genome sequencing was performed on selected strains exhibiting different activity against *F. graminearum*. Genomes of strains with highest antifungal activity were compared with those that showed to be less active and with available reference genomes retrieved from NCBI database. The analyses revealed that all strains are putatively producers of bioactive secondary metabolites and enzymes that hold significant potential for agricultural applications. Specifically, genome-sequenced *Bacillus* strains harbored from 15 to 24 secondary metabolite clusters, suggesting their potential capability to synthesize multiple antimicrobial compounds. Previous studies showed that bacillomycin, fengycin, iturin, and surfactin act synergistically against plant pathogens exhibing enhanced antifungal activity (Mora et al., 2015). Furthermore, the simultaneous production of these compounds enables bacteria to exhibit antagonism against a broader range of microorganisms (Han et al., 2018). All genomes analyzed showed the presence of gene clusters encoding fengycin, surfactin, bacillibactin, mycosubtilin, bacillomycin D, bacillaene and bacilysin. In addition, strain-specific presence of other gene clusters encoding for putative active metabolites was proved. Therefore, all strains likely shared the potential capacity to produce at least seven compounds that are known to act against fungal pathogens disrupting membrane integrity and cell wall structure, or inhibiting protein synthesis (Ntushelo et al., 2019; Tran et al., 2022). Fengycin has demonstrated strong antagonistic activity against *F. graminearum*, inhibiting spore formation and germination (Hanif et al., 2019), and inhibited mycotoxins biosynthesis, including DON (Gu et al., 2017).

Among the species analyzed, *B. velezensis* exhibited the highest number of secondary metabolite clusters, being strain N21.3 the most effective antagonist and the most genetically endowed, devoting approximately 20% of its genome to secondary metabolite synthesis, that is a remarkable proportion compared to the strain FZB42, used as BCA and plant growth promoter, which reserves only about 10% (Fan et al., 2018). Comparative genomics between our *B. velezensis* strains and *B. velezensis* FZB42 and QST713, already used as BCAs, showed that the majority of gene clusters involved in the biocontrol are conserved in *B. velezensis*. However, some gene clusters putatively associated with active metabolites are strain specific. Specifically, genome analysis of *B. velezensis* strains N21.3, N20.3 and S106.1b, and *B. mojavensis* N67. B2 and S110.5 revealed the presence of biosynthetic gene cluster for plipastatin, a well-known compound active against filamentous fungi, including *Fusarium* (Tran et al., 2022). Interestingly, our genomic analysis revealed the potential capability of the strain N21.3 to produce also the active molecules ericin S and rhizocticin A. In fact, it harbors the lantibiotic gene cluster responsible for ericin S biosynthesis, detected to date only in the genomes of other few *B. velezensis* strains (Palazzini et al., 2016; Keshmirshekan et al., 2024). The genome of strain N21.3 also contains the gene cluster for rhizocticin A, an antifungal phosphonate oligopeptide, which to our knowledge has not yet been reported in *B. velezensis* (Tran et al., 2022). Additionally, strain N21.3 harbors numerous unknown clusters, including the NRPS-type clusters with predicted antimicrobial activity (Zhao and Kuipers, 2016).

Within the CAZyme repertoire, hydrolytic enzymes, including chitinases, glucanases, cellulases, lipases, and proteases, were consistently identified in *B. velezensis*, *B. mojavensis*, and *B. amyloliquefaciens*. These enzymes could contribute significantly to antagonistic interactions. Notably, CB family 50, bound to GH18, GH19, GH23, GH24, GH25, and GH73 family enzymes, mediate the cleavage of chitin and peptidoglycan. These enzymes can efficiently hydrolyze key components of fungal and bacterial cell walls, thereby playing a pivotal role in plant pathogen suppression (Bonaterra et al., 2022). Moreover, these enzymes may interfere with quorum sensing systems in pathogens by degrading or inhibiting the signaling molecules synthesis, impairing pathogen colonization and mitigating plant disease symptoms (Kalia et al., 2019).

In all strains, the detection of genes coding for antimicrobial molecules was consistent with predictions based on secondary metabolite gene clusters (antiSMASH). *Bacillus* strains, however, produce various isoforms of the three major lipopeptide families as already observed (Assena et al., 2024). This could explain the no strict correspondence between gene detection and secondary metabolites gene clusters prediction (i.e., positive *ituA* gene detection in *B. mojavensis* strains).

Finally, despite the prediction of gene clusters in the genome, metabolite production could not always occur. Indeed, the detection of gene clusters associated with the biosynthesis of secondary metabolites occurred even in strains that did not show antifungal activity under the same experimental conditions. The differences in antifungal activity observed between strains were likely due to different metabolic potentials, but also to the presence of multiple copies of the same cluster in the genome or, under our experimental conditions, to different levels of gene expression, including a lack of expression of identified metabolic pathways. Further metabolomic and transcriptomic analyses could better elucidate the association between metabolite production and genetic profile.

In this study, some strains of *B. velezensis* emerged as particularly promising antifungal agents. This species, together with *B. amyloliquefaciens* and *B. mojavensis*, is included in the list of microorganisms granted Qualified Presumption of Safety (QPS) status by the European Food Safety Authority (EFSA), as it is considered non-toxic, non-infectious to humans, and not closely related to plant, animal, or human pathogens.

As an additional safety measure, all genomes were screened for the presence of VF genes. The limited number of VF genes were detected. Similar genes have been previously reported to contribute to microbial survival, ecological competitiveness, and adaptation to environmental conditions, including traits relevant to plant-associated and soil-dwelling microorganisms (Silveira et al., 2024). These functions are regarded as important and desirable characteristics for effective biocontrol agents and are not intrinsically linked to virulence in humans or animals. Moreover, no critical virulence determinants were detected. The detected antimicrobial resistance profile was consistent with naturally occurring intrinsic resistance in environmental bacteria and was not considered a barrier to the safe use of the strains as biological control agents for agricultural applications. Furthermore, the observed antimicrobial resistance profile did not differ from that of strains already registered as biocontrol agents.

Overall, the absence of plasmid-borne virulence genes, the very low abundance of putative virulence-associated loci and their functional annotation, and the antimicrobial resistance profiles, in line with EFSA guidelines (Regulation (EC) No 1107/2009), support the conclusion that the selected strains do not pose biosafety concerns and can be considered safe for use as biocontrol agents.

In this study, we also evaluated whether the bacterial strains influenced DON production, since, to ensure food safety, an effective BCA should simultaneously reduce both fungal growth and mycotoxin accumulation (Medina et al., 2017; Einloft et al., 2021). Under the experimental conditions used, all strains appeared able to influence DON production, but different mechanisms were likely involved. In particular, most of the strains with antifungal activity caused complete inhibition of fungal growth, which was associated with the absence of DON detection. Interestingly, the lack of DON detection was also observed in association with bacterial strains that did not show antagonistic activity, suggesting that different factors, including the inhibition of fungal growth, interference with the fungal metabolic pathway involved in DON production, or direct bacterial degradation of DON could co-occur. For instance, Pan et al. (2015) identified a *B. megaterium* strain that significantly reduced both the growth of *F. graminearum* and DON production, while Palazzini et al. (2018) selected a *B. velezensis* strain able to reduce DON accumulation on durum wheat. Moreover, Kim et al. (2017) reported that lipopeptides affected DON biosynthetic gene expression inhibiting DON production. In particular, among lipopeptides, fengycin produced by *B. amyloliquefaciens* was able to reduce *F. graminearum* growth and DON biosynthesis (Hanif et al., 2019).

In conclusion, this study identified novel *Bacillus* strains as promising candidates to control *F. graminearum* growth and to mitigate DON production. Additionally, it provided new insights into *B. velezensis* strains QST7132 and D747, already used in commercial formulations but not yet authorized to control FHB. Comparative genomic analyses revealed key traits for biocontrol, including different secondary metabolite gene clusters and hydrolytic enzymes. Furthermore, these analyses highlighted specific traits that could be exploited for the management of other phytopathogens. Further studies could better elucidate the molecular mechanisms involved in the antifungal activity of bacterial strains analyzed, as well as their role in mitigating DON production. Finally, metabolomic approaches will be essential to confirm the production of the metabolites predicted in this study, and they are in progress.

## Data Availability

The sequencing data generated in this study have been deposited in the NCBI Sequence Read Archive (SRA) under BioProject ID PRJNA1454280.

## Footnotes

1
www.ispa.cnr.it/Collection

2
http://www.mgc.ac.cn/cgi-bin/VFs/v5/main.cgi?func=VFanalyzer

## References

[ref1] Adeniji A. A. Aremu O. S. Babalola O. O. (2019). Selecting lipopeptide-producing, *Fusarium*-suppressing *Bacillus* spp.: Metabolomic and genomic probing of *Bacillus velezensis* NWUMFkBS10.5. Microbiologyopen 8:e742. doi: 10.1002/mbo3.742, 30358165 PMC6562122

[ref2] Arguelles-Arias A. Ongena M. Halimi B. Lara Y. Brans A. Joris B. . (2009). *Bacillus amyloliquefaciens* GA1 as a source of potent antibiotics and other secondary metabolites for biocontrol of plant pathogens. Microb. Cell Factories 8, 1–13. doi: 10.1186/1475-2859-8-63, 19941639 PMC2787494

[ref3] Assena M. W. Pfannstiel J. Rasche F. (2024). Inhibitory activity of bacterial lipopeptides against *Fusarium oxysporum* f. sp. Strigae. BMC Microbiol. 24:227. doi: 10.1186/s12866-024-03386-2, 38937715 PMC11212183

[ref4] Bai G. H. Desjardins A. Plattner R. (2002). Deoxynivalenol-nonproducing *Fusarium graminearum* causes initial infection but does not cause disease spread in wheat spikes. Mycopathologia 153, 91–98. doi: 10.1023/A:1014419323550, 12000132

[ref5] Blin K. Shaw S. Kloosterman A. M. Charlop-Powers Z. van Weezel G. P. Medema M. H. . (2021). antiSMASH 6.0: improving cluster detection and comparison capabilities. Nucleic Acids Res. 49, W29–W35. doi: 10.1093/nar/gkab335, 33978755 PMC8262755

[ref6] Bonaterra A. Badosa E. Daranas N. Francés J. Roselló G. Montesinos E. (2022). Bacteria as biological control agents of plant diseases. Microorganisms 10:1759. doi: 10.3390/microorganisms10091759, 36144361 PMC9502092

[ref7] Carattoli A. Hasman H. (2019). “PlasmidFinder and in Silico pMLST: identification and typing of plasmid replicons in whole-genome sequencing (WGS),” *Methods Mol Biol*. (2020) 2075, 285–294. doi: 10.1007/978-1-4939-9877-7_20 31584170

[ref8] D’Ascanio V. Annunziato A. Carella D. Wiesenberger G. Susca A. Haidukowski M. . (2026). Multitoxin analysis of trichothecenes, including NX toxins, in rice and wheat. Appl. Food Res. 6:101709. doi: 10.1016/j.afres.2026.101709

[ref9] Daranas N. Roselló G. Cabrefiga J. Donati I. Francés J. Badosa E. . (2019). Biological control of bacterial plant diseases with *Lactobacillus plantarum* strains selected for their broad-spectrum activity. Ann. Appl. Biol. 174, 92–105. doi: 10.1111/aab.12476, 30686827 PMC6334523

[ref10] Davis M. J. Purcell A. H. Thompson S. V. (1980). Isolation medium for the Pierce’s disease bacterium. Phytopathology 70, 425–429. doi: 10.1094/Phyto-70-425

[ref11] De Bellis P. Minervini F. Di Biase M. Valerio F. Lavermicocca P. Sisto A. (2015). Toxigenic potential and heat survival of spore-forming bacteria isolated from bread and ingredients. Int. J. Food Microbiol. 197, 30–39. doi: 10.1016/j.ijfoodmicro.2014.12.017, 25555227

[ref12] Dong Q. Liu Q. Goodwin P. H. Deng X. Xu W. Xia M. . (2023). Isolation and genome-based characterization of biocontrol potential of Bacillus siamensis YB-1631 against wheat crown rot caused by Fusarium pseudograminearum. J. Fungi 9:547. doi: 10.3390/jof9050547, 37233258 PMC10219336

[ref13] Dunlap C. A. (2019). Taxonomy of registered Bacillus spp. strains used as plant pathogen antagonists. Biol. Control 134, 82–86. doi: 10.1016/j.biocontrol.2019.04.011

[ref14] Einloft T. C. de Oliveira P. B. Radünz L. L. Dionello R. G. (2021). Biocontrol capabilities of three *Bacillus* isolates towards aflatoxin B1 producer *aspergillus flavus in vitro* and on maize grains. Food Control 125:107978. doi: 10.1016/j.foodcont.2021.107978

[ref40] European Commission (2014). Review report for the active substance Bacillus amyloliquefaciens subsp. plantarum strain D747. SANCO/11391/2014 Rev. 1. Available at: https://ec.europa.eu/food/plant/pesticides/eu-pesticides-database/backend/api/active_substance/download/184

[ref15] Fan B. Wang C. Song X. Ding X. Wu L. Wu H. . (2018). *Bacillus velezensis* FZB42 in 2018: the gram-positive model strain for plant growth promotion and biocontrol. Front. Microbiol. 9:2491. doi: 10.3389/fmicb.2018.02491, 30386322 PMC6198173

[ref16] Gond S. K. Bergen M. S. Torres M. S. White J. F. (2015). Endophytic *Bacillus* spp. produce antifungal lipopeptides and induce host defence gene expression in maize. Microbiol. Res. 172, 79–87. doi: 10.1016/j.micres.2014.11.004, 25497916

[ref17] Gong A. D. Li H. P. Yuan Q. S. Song X. S. Yao W. He W. J. . (2015). Antagonistic mechanism of iturin a and plipastatin a from *Bacillus amyloliquefaciens* S76-3 from wheat spikes against *Fusarium graminearum*. PLoS One 10:e0116871. doi: 10.1371/journal.pone.0116871, 25689464 PMC4331432

[ref18] Gu Q. Yang Y. Yuan Q. Shi G. Wu L. Lou Z. . (2017). Bacillomycin D produced by *Bacillus amyloliquefaciens* is involved in the antagonistic interaction with the plant-pathogenic fungus *Fusarium graminearum*. Appl. Environ. Microbiol. 83, e01075–e01017. doi: 10.1128/AEM.01075-17, 28733288 PMC5601353

[ref19] Guermech S. Somma S. Masiello M. Haidukowski M. Sanzani S. M. Ippolito A. . (2025). Geographical distribution of Fusarium species involved in Fusarium head blight and Fusarium crown rot of wheat in Tunisia and their mycotoxin accumulation. Plant Pathol. 74, 1290–1301. doi: 10.1111/ppa.14092

[ref20] Haddoudi I. Cabrefiga J. Mora I. Mhadhbi H. Montesinos E. Mrabet M. (2021). Biological control of Fusarium wilt caused by Fusarium equiseti in Vicia faba with broad spectrum antifungal plant-associated Bacillus spp. Biol. Control 160:104671. doi: 10.1016/j.biocontrol.2021.104671

[ref21] Han J. W. Choi G. J. Kim B. S. (2018). Antimicrobial aromatic polyketides: a review of their antimicrobial properties and potential use in plant disease control. World J. Microbiol. Biotechnol. 34:163. doi: 10.1007/s11274-018-2546-0, 30368604

[ref22] Hanif A. Zhang F. Li P. Li C. Xu Y. Zubair M. . (2019). *Fengycin* produced *by Bacillus amyloliquefaciens* FZB42 inhibits *Fusarium graminearum* growth and mycotoxins biosynthesis. Toxins 11:295. doi: 10.3390/toxins11050295, 31137632 PMC6563212

[ref23] Horan K. da Silva A. G. Perry A. (2023). MDU-PHL/abritamr: v1.0.20b (v1.0.20b). doi: 10.5281/zenodo.10369242

[ref24] Kalia V. C. Patel S. K. S. Kang Y. C. Lee J. K. (2019). Quorum sensing inhibitors as antipathogens: biotechnological applications. Biotechnol. Adv. 37, 68–90. doi: 10.1016/j.biotechadv.2018.11.006, 30471318

[ref25] Keshmirshekan A. de Souza Mesquita L. M. Ventura S. P. M. (2024). Biocontrol manufacturing and agricultural applications of *Bacillus velezensis*. Trends Biotechnol. 42, 986–1001. doi: 10.1016/j.tibtech.2024.02.003, 38448350

[ref26] Khan M. Salman M. Jan S. A. Shinwari Z. K. (2021). Biological control of fungal phytopathogens: a comprehensive review based on *Bacillus* species. MOJ Biol. Med. 6, 90–92. doi: 10.15406/mojbm.2021.06.00137

[ref27] Kim K. Lee Y. Ha A. Kim J. I. Park A. R. Yu N. H. . (2017). Chemosensitization of *Fusarium graminearum* to chemical fungicides using cyclic Lipopeptides produced by *Bacillus amyloliquefaciens* strain JCK-12. Front. Plant Sci. 8:2010. doi: 10.3389/fpls.2017.02010, 29230232 PMC5711811

[ref28] Ma G. Wang H. Qi K. Ma L. Zhang B. Zhang Y. . (2024). Isolation, characterization, and pathogenicity of *Fusarium* species causing crown rot of wheat. Front. Microbiol. 15:1405115. doi: 10.3389/fmicb.2024.1405115, 38873144 PMC11169711

[ref29] Medina A. Mohale S. Samsudin N. Rodriguez-Sixtos A. Rodriguez A. Magan N. (2017). Biocontrol of mycotoxins: dynamics and mechanisms of action. Curr. Opin. Food Sci. 17, 41–48. doi: 10.1016/j.cofs.2017.09.008

[ref30] Mizumoto S. Shoda M. (2007). Medium optimization of antifungal lipopeptide, iturin a, production by *Bacillus subtilis* in solid-state fermentation by response surface methodology. Appl. Microbiol. Biotechnol. 76, 101–108. doi: 10.1007/s00253-007-0994-9, 17476498

[ref31] Mora I. Cabrefiga J. Montesinos E. (2011). Antimicrobial peptide genes in *Bacillus* strains from plant environments. Int. Microbiol. 14, 213–223. doi: 10.2436/20.1501.01.151, 22569759

[ref32] Mora I. Cabrefiga J. Montesinos E. (2015). Cyclic lipopeptide biosynthetic genes and products, and inhibitory activity of plant-associated Bacillus against phytopathogenic bacteria. PLoS One 10:e0127738. doi: 10.1371/journal.pone.0127738, 26024374 PMC4449161

[ref33] Munkvold G. P. Proctor R. H. Moretti A. (2021). Mycotoxin production in *Fusarium* according to contemporary species concepts. Annu. Rev. Phytopathol. 59, 373–402. doi: 10.1146/annurev-phyto-020620-102825, 34077240

[ref34] Ntushelo K. Ledwaba L. K. Rauwane M. E. Adebo O. A. Njobeh P. B. (2019). The mode of action of Bacillus species against Fusarium graminearum, tools for investigation, and future prospects. Toxins 11:606. doi: 10.3390/toxins11100606, 31635255 PMC6832908

[ref35] Palazzini J. M. Dunlap C. A. Bowman M. J. Chulze S. N. (2016). *Bacillus velezensis* RC 218 as a biocontrol agent to reduce Fusarium head blight and deoxynivalenol accumulation: genome sequencing and secondary metabolite cluster profiles. Microbiol. Res. 192, 30–36. doi: 10.1016/j.micres.2016.06.002, 27664721

[ref36] Palazzini J. Roncallo P. Cantoro R. Chiotta M. Yerkovich N. Palacios S. . (2018). Biocontrol of Fusarium graminearum sensu stricto, reduction of deoxynivalenol accumulation and phytohormone induction by two selected antagonists. Toxins 10:88. doi: 10.3390/toxins10020088, 29461480 PMC5848189

[ref37] Pan D. Mionetto A. Tiscornia S. Bettucci L. (2015). Endophytic bacteria from wheat grain as biocontrol agents of *Fusarium graminearum* and deoxynivalenol production in wheat. Mycotoxin Res. 31, 137–143. doi: 10.1007/s12550-015-0224-8, 25956808

[ref38] Pandin C. Le Coq D. Deschamps J. Védie R. Rousseau T. Aymerich S. . (2018). Complete genome sequence of *Bacillus velezensis* QST713: a biocontrol agent that protects *Agaricus bisporus* crops against the green mould disease. J. Biotechnol. 278, 10–19. doi: 10.1016/j.jbiotec.2018.04.014, 29702132

[ref39] Proctor R. H. Hohn T. M. McCormick S. P. (1995). Reduced virulence of *Gibberella zeae* caused by disruption of a trichothecene toxin biosynthetic gene. Mol. Plant-Microbe Interact. 8, 593–601. doi: 10.1094/mpmi-8-0593, 8589414

[ref41] Seemann T. (2016). ABRicate: Mass Screening of Contigs for Antiobiotic Resistance Genes. Available online at: https://github.com/tseemann/abricate (Accessed February 20, 2026).

[ref42] Silveira R. D. Fonseca Veras F. Cardoso Hernandes K. Bach E. Pereira Passaglia L. M. Alcaraz Zini C. . (2024). Genomic analysis reveals genes that encode the synthesis of volatile compounds by a *Bacillus velezensis*-based biofungicide used in the treatment of grapes to control *aspergillus carbonarius*. Int. J. Food Microbiol. 415:110644. doi: 10.1016/j.ijfoodmicro.2024.110644, 38417280

[ref43] Sobrova P. Adam V. Vasatkova A. Beklova M. Zeman L. Kizek R. (2010). Deoxynivalenol and its toxicity. Interdiscip. Toxicol. 3, 94–99. doi: 10.2478/v10102-010-0019-x 21217881 PMC2984136

[ref44] Tran C. Cock I. E. Chen X. Feng Y. (2022). Antimicrobial *Bacillus*: metabolites and their mode of action. Antibiotics 11:88. doi: 10.3390/antibiotics11010088, 35052965 PMC8772736

[ref45] Valerio F. De Bellis P. Di Biase M. Lonigro S. L. Giussani B. Visconti A. . (2012). Diversity of spore-forming bacteria and identification of *Bacillus amyloliquefaciens* as a species frequently associated with the ropy spoilage of bread. Int. J. Food Microbiol. 156, 278–285. doi: 10.1016/j.ijfoodmicro.2012.04.005, 22551674

[ref46] Wang Y. Pei Y. Wang X. Dai X. Zhu M. (2024). Antimicrobial metabolites produced by the plant growth-promoting rhizobacteria (PGPR): *Bacillus* and *Pseudomonas*. Adv. Agrochem 3, 206–221. doi: 10.1016/j.aac.2024.07.007

[ref47] Wood D. E. Salzberg S. L. (2014). Kraken: ultrafast metagenomic sequence classification using exact alignments. Genome Biol. 15:R46. doi: 10.1186/gb-2014-15-3-r46, 24580807 PMC4053813

[ref48] Yuan Y. Zhang S. Tan X. Deng J. Gong S. Zhai X. . (2024). Intestinal bacterium *Bacillus siamensis* M54 from *Allomyrina dichotoma* is a potential biocontrol agent against maize stalk rot. Biol. Control 199:105660. doi: 10.1016/j.biocontrol.2024.105660

[ref49] Zalila-Kolsi I. Mahmoud A. B. Ali H. Sellami S. Nasfi Z. Tounsi S. . (2016). Antagonist effects of *Bacillus* spp. strains against *Fusarium graminearum* for protection of durum wheat (*Triticum turgidum* L. subsp. *durum*). *Microbiol*. Res. 192, 148–158. doi: 10.1016/j.micres.2016.06.012, 27664733

[ref50] Zanon M. S. A. Rosales Cavaglieri L. Palazzini J. M. Chulze S. N. Chiotta M. L. (2024). *Bacillus velezensis* RC218 and emerging biocontrol agents against *Fusarium graminearum* and *Fusarium poae* in barley: *in vitro*, greenhouse and field conditions. Int. J. Food Microbiol. 413:110580. doi: 10.1016/j.ijfoodmicro.2024.110580, 38246027

[ref51] Zhang L. Shao L. Zhang B. Li Y. Wang Z. Song S. . (2026). Genome analysis and secondary metabolite characterization of Bacillus velezensis JZ as a potential biocontrol agent. Physiol. Mol. Plant Pathol. 141:103015. doi: 10.1016/j.pmpp.2025.103015

[ref52] Zhang K. Wang L. Si H. Guo H. Liu J. Jia J. . (2022). Maize stalk rot caused by Fusarium graminearum alters soil microbial composition and is directly inhibited by Bacillus siamensis isolated from rhizosphere soil. Front. Microbiol. 13:986401. doi: 10.3389/fmicb.2022.986401, 36338067 PMC9630747

[ref53] Zhao X. Kuipers O. P. (2016). Identification and classification of known and putative antimicrobial compounds produced by a wide variety of *Bacillales* species. BMC Genomics 17:882. doi: 10.1186/s12864-016-3224-y, 27821051 PMC5100339

[ref54] Zhao Y. Selvaraj J. N. Xing F. Zhou L. Wang Y. Song H. . (2014). Antagonistic action of Bacillus subtilis strain SG6 on Fusarium graminearum. PLoS One 9:e92486. doi: 10.1371/journal.pone.0092486, 24651513 PMC3961383

[ref55] Zheng J. Ge Q. Yan Y. Zhang X. Huang L. Yin Y. (2023). dbCAN3: automated carbohydrate-active enzyme and substrate annotation. Nucleic Acids Res. 51, W115–W121. doi: 10.1093/nar/gkad328, 37125649 PMC10320055

[ref56] Zicca S. De Bellis P. Masiello M. Saponari M. Saldarelli P. Boscia D. . (2020). Antagonistic activity of olive endophytic bacteria and of *Bacillus* spp. strains against *Xylella fastidiosa*. Microbiol. Res. 236:126467. doi: 10.1016/j.micres.2020.126467, 32248049

