# Supplementary material for: Genomic and functional characterization of Bacillus strains active against Fusarium graminearum
Source: Front Microbiol. 2026 May 29;17:1832933. doi: 10.3389/fmicb.2026.1832933 (PMC13260424; doi:10.3389/fmicb.2026.1832933)
Supplement: Supplementary file 1 [file Table_3.pdf]

Supplementary Table 3. CAZymes prediction, according to three tools (HMMER, DIAMOND, dbCAN) in the genomes of 10 *Bacillus* strains selected for whole-genome sequencing.

N3.2_overview

Gene ID	EC#	HMMER	dbCAN_sub	DIAMOND	Signalp	#ofTools
contig_11_142	-	CE14(8-113)	CE14_e0	CE14	N	3
contig_11_143	2.4.1.-	GT4(196-344)	GT4_e1442	GT4	N	3
contig_11_157	2.4.1.129	GT51(87-263)	GT51_e113	GT51	N	3
contig_11_197	2.4.1.157	GT28(204-354)	GT28_e89	GT28	N	3
contig_12_6	-	GT2(49-279)	GT2	GT2	N	3
contig_12_9	-	GH126(42-350)	GH126_e0	GH126	Y(1-25)	3
contig_13_62	-	GT83(8-225)	GT83_e60	N	N	2
contig_13_63	-	GT4(251-402)	GT4_e2274	GT4	N	3
contig_13_66	-	GT4(253-406)	GT4_e3738	GT4	N	3
contig_13_77	2.4.1.10	GH68_1(42-469)	GH68_e0	GH68_1	Y(1-30)	3
contig_13_78	3.2.1.65|3.2.1.64	GH32(64-369)	GH32_e117	GH32	N	3
contig_14_58	N	AA1(48-499)	N	N	N	1
contig_15_108	3.2.1.99	GH43_4(33-359)	GH43_e38	GH43_4	Y(1-26)	3
contig_15_139	-	GH177(3-295)	GH109_e5	N	N	2
contig_15_22	-	GT8(5-257)	GT8_e207	GT8	N	3
contig_15_33	3.2.1.86	GH4(6-185)	GH4_e24	GH4	N	3
contig_15_48	3.2.1.86|3.2.1.85|3.2.1.21	GH1(7-465)	GH1_e13	GH1	N	3
contig_15_51	3.2.1.78|3.2.1.-	GH26(30-348)	GH26_e0	GH26	Y(1-25)	3
contig_15_61	-	CE4(114-242)	CE4_e274	N	Y(1-28)	2
contig_15_7	-	GT2(8-218)	GT2	N	N	2
contig_15_78	3.2.1.73|3.2.1.6|3.2.1.8	GH16_21(38-238)	GH16_e14	GH16_21	Y(1-30)	3
contig_15_95	-	PL1_8(88-284)	PL1_e106	PL1_8	Y(1-36)	3
contig_17_132	-	GH18(31-301)	GH18_e71	GH18	N	3
contig_17_182	-	GH18(171-401)	CBM50_e96+CBM50_e96+GH18_e174	CBM50+GH18	N	3
contig_17_21	N	GT119(18-374)	N	GT119	N	2
contig_17_40	-	CE4(73-195)	CE4_e129	N	Y(1-33)	2
contig_18_117	4.2.2.2	PL1_6(135-348)	PL1_e108	PL1_6	Y(1-22)	3
contig_18_127	-	GT2(5-126)	GT2	GT2	N	3
contig_18_134	-	GT2(4-129)	GT2	GT2	N	3
contig_18_16	-	GT2(7-168)	GT2	GT2	N	3
contig_18_51	3.2.1.122	GH4(8-185)	GH4_e4	GH4	N	3
contig_18_67	3.5.1.104	CE4(59-185)	CE4_e23	CE4	Y(1-24)	3
contig_18_89	3.2.1.93	GH13_29(30-376)	GH13_e1	GH13_29	N	3
contig_1_89	1.1.3.-	AA7(23-242)	AA7_e0	N	N	2
contig_20_29	3.2.1.55|3.2.1.8|3.2.1.-|3.2.1.37	GH43_16(37-359)+CBM6(381-510)	GH43_e261+CBM6_e6	CBM6+GH43_16	Y(1-26)	3
contig_20_30	3.2.1.8|3.2.1.136	GH30_8(33-419)	GH30_e78	GH30_8	Y(1-33)	3
contig_20_34	3.2.1.4|3.2.1.78|3.2.1.132	GH5_2(53-290)+CBM3(357-437)	GH5_e251+CBM3_e18	CBM3+GH5_2	Y(1-30)	3
contig_20_88	1.14.99.53	AA10(28-203)	AA10_e1	AA10	N	3
contig_21_18	3.2.1.99	GH43_5(41-319)	GH43_e56	GH43_5	Y(1-31)	3
contig_21_6	-	AA4(11-236)	AA4_e1	N	N	2
contig_21_9	3.2.1.55	GH51_1(7-496)	GH51_e0	GH51_1	N	3
contig_23_103	N	CE1(31-261)	N	N	N	1
contig_23_13	3.2.1.-	GH73(58-193)	GH73_e25	GH73	Y(1-34)	3
contig_23_19	N	GH188(2-145)	N	N	N	1
contig_23_30	5.4.99.11|3.2.1.20|3.2.1.10|3.2.1.-|3.2.1.70	GH13_31(28-371)	GH13_e122	GH13_31	N	3
contig_23_54	-	GT51(64-238)	GT51_e55	GT51	N	3
contig_24_106	-	GT2(4-161)	GT2	GT2	N	3
contig_24_24	3.1.1.41|3.1.1.72	CE7(4-312)	CE7_e11	CE7	N	3
contig_24_38	3.2.1.1	GH13_28(58-339)+CBM26(565-642)	GH13_e34+CBM26_e0	CBM26+GH13_28	Y(1-32)	3
contig_24_56	5.4.99.11|3.2.1.20|3.2.1.10|3.2.1.-|3.2.1.70	GH13_31(28-375)	GH13_e122	GH13_31	N	3
contig_24_7	3.2.1.86|3.2.1.38|3.2.1.85|3.2.1.21|3.2.1.-	GH1(8-475)	GH1_e115	GH1	N	3
contig_25_1	-	CE4(52-178)	CE4_e14	CE4	N	3
contig_26_11	3.2.1.17|4.2.2.n1	GH23(981-1093)	GH23_e335	GH23	N	3
contig_27_2	3.2.1.17|4.2.2.n1	GH23(1441-1553)	GH23_e335	GH23	N	3
contig_2_15	3.2.1.26	GH32(33-338)	GH32_e57	GH32	N	3
contig_2_167	3.2.1.8	GH11(32-211)	GH11_e15	GH11	Y(1-29)	3
contig_2_176	N	GT119(17-362)	N	GT119	N	2
contig_2_21	-	GT2(4-162)	GT2	GT2	N	3
contig_2_254	-	GT2(4-133)	GT2	GT2	N	3
contig_2_27	-	GT2(5-168)	GT2	GT2	N	3
contig_2_8	N	GT119(19-384)	N	GT119	N	2
contig_2_84	-	GT51(63-237)	GT51_e8	GT51	N	3
contig_33_16	-	GT2(6-168)	GT2	GT2	N	3
contig_33_40	3.2.1.86|3.2.1.23|3.2.1.21	GH1(3-476)	GH1_e0	GH1	N	3
contig_33_41	-	GH30_3(81-424)	GH30_e56	GH30_3	Y(1-30)	3
contig_33_55	-	CE14(6-124)	CE14_e39	N	N	2
contig_33_60	-	GT1(9-393)	GT1_e505	GT1	N	3
contig_35_16	N	GT119(2-337)	N	GT119	N	2
contig_35_17	-	GT28(188-350)	GT28_e1	GT28	N	3
contig_36_12	N	CE1(14-241)	N	N	N	1
contig_36_6	3.2.1.52	GH3(108-343)	GH3_e28	GH3	Y(1-21)	3
contig_36_7	3.2.1.92	GH171(57-414)	GH171_e0	GH171	Y(1-24)	3
contig_3_107	3.2.1.85	GH1(3-463)	GH1_e65	GH1	N	3
contig_3_113	-	GH53(40-341)	GH53_e0	GH53	Y(1-35)	3
contig_3_141	N	CE1(15-237)	N	N	N	1
contig_3_177	3.2.1.17|4.2.2.n1	GH23(123-226)	GH23_e335	GH23	N	3
contig_3_2	-	GT2(11-95)	GT2	GT2	N	3
contig_3_30	-	GT2(8-170)	GT2	GT2	N	3
contig_3_305	2.4.1.129	GT51(55-229)	GT51_e20	GT51	Y(1-60)	3
contig_3_31	-	GT83(10-262)	GT83_e35	N	N	2
contig_3_352	-	CE4(81-207)	CE4_e25	CE4	Y(1-42)	3
contig_3_38	-	CBM50(161-203)	CBM50_e826	CBM50	N	3
contig_3_411	-	AA6(2-169)	AA6_e2	N	N	2
contig_3_441	1.1.3.-	AA7(21-243)	AA7_e0	N	N	2
contig_3_96	2.4.1.-	GT1(184-387)	GT1_e225	GT1	N	3
contig_40_3	3.2.1.17|4.2.2.n1	GH23(997-1109)	GH23_e335	GH23	N	3
contig_46_2	-	GT4(343-492)	GT4_e2213	GT4	N	3
contig_46_4	2.4.1.-	GT26(57-224)	GT26_e19	GT26	N	3
contig_46_6	3.2.1.-|3.2.1.17	GH73(734-861)	GH73_e162	GH73	Y(1-28)	3
contig_4_114	3.2.1.37|3.2.1.55|3.2.1.-|-	GH43_11(3-301)+CBM91(331-532)	GH43_e285+CBM91_e24	CBM91+GH43_11	N	3
contig_5_5	-	GT1(115-391)	GT1_e274	GT1	N	3
contig_60_6	3.2.1.22	GH4(3-180)	GH4_e17	GH4	N	3
contig_62_11	-	CE4(117-241)	CE4_e20	CE4	N	3
contig_64_10	3.2.1.-	GH18(126-404)	CBM50_e699+GH18_e157	CBM50+GH18	N	3
contig_7_197	N	CE12(30-200)	N	N	Y(1-26)	1
contig_7_198	-	CE4(42-163)	CE4_e317	CE4	Y(1-24)	3
contig_7_202	-	CE6(55-146)	CE6_e1	CE0	N	3
contig_7_294	-	CBM50(4-48)	CBM50_e257	CBM50	N	3
contig_7_367	3.2.1.55	GH51_1(7-490)	GH51_e19	GH51_1	N	3
contig_8_100	3.2.1.54|3.2.1.133|3.2.1.135	CBM34(6-126)+GH13_20(175-468)	CBM34_e0+GH13_e225	CBM34+GH13_20	N	3
contig_8_105	2.4.1.8	GH65(319-687)	GH65_e0	GH65	N	3
contig_8_106	5.4.99.11|3.2.1.20|3.2.1.10|3.2.1.-|3.2.1.70	GH13_31(27-377)	GH13_e122	GH13_31	N	3
contig_8_11	-	GT4(215-345)	GT4_e23	GT4	N	3
contig_8_111	3.2.1.26	GH32(27-331)	GH32_e0	GH32	N	3
contig_8_127	-	GT4(193-345)	GT4_e2316	GT4	N	3
contig_8_128	-	GT2(10-163)	GT2	GT2	N	3
contig_8_129	-	GT4(197-305)	GT4_e1131	GT4	N	3
contig_8_130	N	GT122(39-352)	N	GT122	N	2
contig_8_131	-	GT2(7-147)	GT2	GT2	N	3
contig_8_133	-	GT2(7-133)	GT2	GT2	N	3
contig_8_15	-	GT2(9-123)	GT2	GT2	N	3
contig_8_212	N	GH109(1-153)	N	N	N	1
contig_8_306	3.2.1.132	GH46(38-262)	GH46_e0	GH46	Y(1-37)	3
contig_8_46	-	PL9_2(33-390)	PL9_e18	PL9_2	Y(1-28)	3
contig_8_67	3.5.1.25	CE9(8-384)	CE9_e51	CE9	N	3
contig_12_1	-	N	CBM50_e826	N	N	1
contig_17_105	-	N	CBM50_e55	N	N	1
contig_17_134	-	N	CBM50_e1022	CBM50	N	2
contig_33_91	-	N	GH23_e590	GH23	N	2
contig_3_52	-	N	GH23_e126	GH0	N	2
contig_6_60	-	N	CBM50_e932	CBM50	N	2
contig_6_73	-	N	CBM50_e826	CBM50	N	2
contig_7_322	-	N	CBM50_e1	CBM50	N	2
contig_11_88	-	N	CBM50_e1023	N	N	1
contig_17_133	-	N	CBM50_e77	N	N	1
contig_33_61	-	N	CBM50_e817+CBM50_e1004+CBM50_e	CBM50	Y(1-27)	2
contig_33_85	-	N	CBM50_e375+CBM50_e520	CBM50	Y(1-24)	2
contig_3_373	-	N	CBM50_e520+CBM50_e438	CBM50	Y(1-26)	2
contig_3_378	-	N	CBM50_e833+CBM50_e1004+CBM50_e	CBM50	Y(1-27)	2
contig_3_51	-	N	CBM50_e932	CBM50	N	2
contig_3_78	-	N	CBM50_e826	CBM50	N	2
contig_6_59	-	N	GH23_e126	GH23	N	2
contig_8_16	-	N	GT4_e101	GT4	N	2
contig_2_11	N	N	N	GH0	Y(1-26)	1
contig_2_33	N	N	N	GT0	N	1
contig_2_128	N	N	N	GH23	N	1
contig_2_139	N	N	N	GH13_11	N	1
contig_2_148	N	N	N	CBM48+GH13_8	N	1
contig_2_202	N	N	N	GT2	N	1
contig_3_181	N	N	N	CBM50	N	1
contig_3_384	N	N	N	GH130_1	N	1
contig_3_396	N	N	N	CBM50	Y(1-29)	1
contig_3_401	N	N	N	GH1	N	1
contig_3_434	N	N	N	GH38	N	1
contig_7_1	N	N	N	GT2	N	1
contig_7_14	N	N	N	GT30	N	1
contig_7_19	N	N	N	GH101	N	1
contig_7_174	N	N	N	GT1	N	1
contig_7_178	N	N	N	GH28	N	1
contig_7_228	N	N	N	GT4	N	1
contig_7_319	N	N	N	GH5_11	N	1
contig_7_331	N	N	N	GT2	N	1
contig_7_337	N	N	N	GT2	N	1
contig_8_4	N	N	N	GT0	N	1
contig_8_12	N	N	N	GT2	N	1
contig_8_101	N	N	N	CBM50+CE4	Y(1-34)	1
contig_8_102	N	N	N	GH13_48	N	1
contig_8_134	N	N	N	GT2	N	1
contig_8_163	N	N	N	GH13_31	N	1
contig_8_215	N	N	N	CBM50	N	1
contig_8_245	N	N	N	CE8	N	1
contig_8_325	N	N	N	CE4	N	1
contig_11_1	N	N	N	GT0	N	1
contig_11_4	N	N	N	GT2	N	1
contig_11_128	N	N	N	GH1	N	1
contig_11_137	N	N	N	CE8	N	1
contig_11_149	N	N	N	GH24	N	1
contig_12_8	N	N	N	GH126	N	1
contig_12_43	N	N	N	GH36	N	1
contig_12_87	N	N	N	GT2	N	1
contig_13_14	N	N	N	GH13_3	N	1
contig_13_38	N	N	N	GT22	N	1
contig_13_75	N	N	N	CBM50	N	1
contig_13_99	N	N	N	GH9	N	1
contig_14_29	N	N	N	GH13_23	N	1
contig_14_52	N	N	N	GH131	N	1
contig_15_94	N	N	N	CBM0+CBM16	Y(1-33)	1
contig_16_1	N	N	N	GT2	N	1
contig_16_2	N	N	N	GT1	N	1
contig_17_48	N	N	N	GT2	N	1
contig_17_51	N	N	N	GT2	N	1
contig_17_53	N	N	N	GT2	N	1
contig_17_145	N	N	N	GT4	N	1
contig_18_49	N	N	N	GH4	N	1
contig_18_84	N	N	N	CBM50	Y(1-36)	1
contig_18_133	N	N	N	GT0	N	1
contig_18_135	N	N	N	GT0	N	1
contig_18_140	N	N	N	GT4	N	1
contig_19_15	N	N	N	GH84	N	1
contig_19_46	N	N	N	GT50	N	1
contig_19_59	N	N	N	GT119	N	1
contig_20_1	N	N	N	GT1	N	1
contig_20_2	N	N	N	GT2	N	1
contig_20_26	N	N	N	GT2	N	1
contig_20_27	N	N	N	GT1	N	1
contig_20_28	N	N	N	GT2	N	1
contig_20_82	N	N	N	GH0	N	1
contig_21_30	N	N	N	GH28	N	1
contig_21_63	N	N	N	GH13_30	N	1
contig_21_122	N	N	N	GT2	N	1
contig_21_129	N	N	N	GT4	N	1
contig_22_1	N	N	N	GT0	N	1
contig_22_4	N	N	N	GT2	N	1
contig_23_98	N	N	N	GT1	N	1
contig_23_140	N	N	N	GH0	Y(1-18)	1
contig_24_1	N	N	N	GT1	N	1
contig_24_73	N	N	N	CBM6	N	1
contig_24_96	N	N	N	GH0	N	1
contig_25_47	N	N	N	CE4	N	1
contig_25_51	N	N	N	GH1	N	1
contig_25_72	N	N	N	GT4	N	1
contig_26_9	N	N	N	GH113	N	1
contig_27_1	N	N	N	GH113	N	1
contig_29_2	N	N	N	GT2	N	1
contig_29_3	N	N	N	GT0	N	1
contig_29_4	N	N	N	GT2	N	1
contig_29_5	N	N	N	GT2	N	1
contig_29_45	N	N	N	GT2	N	1
contig_30_33	N	N	N	GH13_3	N	1
contig_31_1	N	N	N	GH23	N	1
contig_33_65	N	N	N	GT2	N	1
contig_33_72	N	N	N	GH92	N	1
contig_35_35	N	N	N	GT0	N	1
contig_35_58	N	N	N	CBM50	N	1
contig_35_101	N	N	N	GT2	N	1
contig_35_149	N	N	N	CBM50+GH25	N	1
contig_35_155	N	N	N	GT2	N	1
contig_35_161	N	N	N	GH13_48	N	1
contig_37_12	N	N	N	GH28	N	1
contig_38_14	N	N	N	GH13	N	1
contig_40_1	N	N	N	GH113	N	1
contig_45_9	N	N	N	GH23	N	1
contig_46_1	N	N	N	GT2	N	1
contig_47_1	N	N	N	GT1	N	1
contig_55_1	N	N	N	GT1	N	1
contig_55_2	N	N	N	GT1	N	1
contig_56_11	N	N	N	GH0	N	1
contig_60_5	N	N	N	GH4	Y(1-37)	1
contig_60_9	N	N	N	GT4	N	1
contig_64_4	N	N	N	CBM13	N	1
contig_71_2	N	N	N	GH0	N	1

N21.3_overview

Gene ID	EC#	HMMER	dbCAN_sub	DIAMOND	Signalp	#ofTools
contig_10_30	3.2.1.22	GH4(3-180)	GH4_e17	GH4	N	3
contig_12_1	-	GT2(7-168)	GT2	GT2	N	3
contig_12_34	3.2.1.122	GH4(8-185)	GH4_e4	GH4	N	3
contig_12_50	3.5.1.104	CE4(59-185)	CE4_e23	CE4	N	3
contig_12_72	3.2.1.93	GH13_29(30-376)	GH13_e1	GH13_29	N	3
contig_13_101	N	AA1(48-499)	N	N	N	1
contig_13_14	-	GT2(5-126)	GT2	GT2	N	3
contig_13_21	-	GT2(4-129)	GT2	GT2	N	3
contig_13_4	4.2.2.2	PL1_6(135-348)	PL1_e108	PL1_6	N	3
contig_14_111	3.2.1.55	GH51_1(7-490)	GH51_e19	GH51_1	N	3
contig_14_183	-	CBM50(4-48)	CBM50_e257	CBM50	N	3
contig_14_81	3.2.1.99	GH43_5(41-319)	GH43_e56	GH43_5	N	3
contig_14_90	3.2.1.55	GH51_1(7-496)	GH51_e0	GH51_1	N	3
contig_14_93	-	AA4(11-237)	AA4_e1	N	N	2
contig_15_37	-	GH73(12-145)	GH73_e27	GH73	N	3
contig_15_85	-	CE6(55-146)	CE6_e1	CE0	N	3
contig_15_96	-	CE4(42-163)	CE4_e317	CE4	N	3
contig_15_97	N	CE12(30-200)	N	N	N	1
contig_16_112	3.2.1.86|3.2.1.38|3.2.1.85|3.2.1.21|3.2.1.-	GH1(8-475)	GH1_e115	GH1	N	3
contig_16_13	-	GT2(4-161)	GT2	GT2	N	3
contig_16_63	5.4.99.11|3.2.1.20|3.2.1.10|3.2.1.-|3.2.1.70	GH13_31(28-375)	GH13_e122	GH13_31	N	3
contig_16_81	3.2.1.1	GH13_28(58-339)+CBM26(565-642)	GH13_e34+CBM26_e0	CBM26+GH13_28	N	3
contig_16_95	3.1.1.41|3.1.1.72	CE7(4-312)	CE7_e11	CE7	N	3
contig_17_24	2.4.1.129	GT51(87-263)	GT51_e113	GT51	N	3
contig_17_38	2.4.1.-	GT4(196-344)	GT4_e1442	GT4	N	3
contig_17_39	-	CE14(8-113)	CE14_e0	CE14	N	3
contig_19_137	N	GT119(17-362)	N	GT119	N	2
contig_19_146	3.2.1.8	GH11(32-211)	GH11_e15	GH11	N	3
contig_19_38	-	GT2(9-123)	GT2	GT2	N	3
contig_19_42	-	GT4(215-346)	GT4_e23	GT4	N	3
contig_19_54	-	GT4(220-369)	GT4_e2213	GT4	N	3
contig_19_57	2.4.1.-	GT26(57-224)	GT26_e19	GT26	N	3
contig_19_59	3.2.1.-|3.2.1.17	GH73(734-861)	GH73_e162	GH73	N	3
contig_19_64	-	GT2(4-139)	GT2	GT2	N	3
contig_19_7	-	PL9_2(31-390)	PL9_e18	PL9_2	N	3
contig_1_102	-	CBM50(161-203)	CBM50_e826	CBM50	N	3
contig_1_160	2.4.1.-	GT1(176-388)	GT1_e225	GT1	N	3
contig_1_173	3.2.1.85	GH1(3-463)	GH1_e65	GH1	N	3
contig_1_179	-	GH53(40-341)	GH53_e0	GH53	N	3
contig_1_201	N	CE1(15-237)	N	N	N	1
contig_1_237	3.2.1.17|4.2.2.n1	GH23(123-226)	GH23_e335	GH23	N	3
contig_1_365	2.4.1.129	GT51(55-229)	GT51_e20	GT51	N	3
contig_1_411	-	CE4(81-207)	CE4_e25	CE4	N	3
contig_1_56	-	GH18(171-401)	CBM50_e96+CBM50_e96+GH18_e174	CBM50+GH18	N	3
contig_1_66	-	GT2(11-95)	GT2	GT2	N	3
contig_1_7	-	GH18(31-301)	GH18_e71	GH18	N	3
contig_1_94	-	GT2(8-170)	GT2	GT2	N	3
contig_1_95	-	GT83(11-262)	GT83_e35	N	N	2
contig_21_94	2.4.1.157	GT28(204-354)	GT28_e89	GT28	N	3
contig_23_102	3.2.1.17|4.2.2.n1	GH23(1437-1549)	GH23_e335	GH23	N	3
contig_24_20	1.1.3.-	AA7(80-301)	AA7_e0	N	N	2
contig_25_114	-	GT2(6-168)	GT2	GT2	N	3
contig_25_70	-	GT1(9-393)	GT1_e505	GT1	N	3
contig_25_75	-	CE14(6-124)	CE14_e39	N	N	2
contig_25_89	-	GH30_3(82-488)	GH30_e56	GH30_3	N	3
contig_25_90	3.2.1.86|3.2.1.23|3.2.1.21	GH1(3-476)	GH1_e0	GH1	N	3
contig_29_22	-	AA6(2-169)	AA6_e2	N	N	2
contig_2_10	3.2.1.-	GH18(126-404)	CBM50_e699+GH18_e157	CBM50+GH18	N	3
contig_31_12	N	CE1(14-241)	N	N	N	1
contig_31_6	3.2.1.52	GH3(108-343)	GH3_e28	GH3	N	3
contig_31_7	3.2.1.92	GH171(57-414)	GH171_e0	GH171	N	3
contig_3_131	3.5.1.25	CE9(8-384)	CE9_e51	CE9	N	3
contig_3_29	-	CE14(4-114)	CE14_e46	N	N	2
contig_3_71	-	GT2(7-131)	GT2	GT2	N	3
contig_3_73	-	GT2(7-136)	GT2	GT2	N	3
contig_3_74	N	GT122(39-352)	N	GT122	N	2
contig_3_75	-	GT4(199-305)	GT4_e1131	GT4	N	3
contig_3_76	-	GT2(10-163)	GT2	GT2	N	3
contig_3_77	-	GT4(193-345)	GT4_e2316	GT4	N	3
contig_3_91	3.2.1.26	GH32(27-331)	GH32_e0	GH32	N	3
contig_42_11	3.2.1.-	GH73(58-193)	GH73_e25	GH73	N	3
contig_4_75	-	CE4(52-178)	CE4_e14	CE4	N	3
contig_5_125	3.2.1.132	GH46(38-262)	GH46_e0	GH46	N	3
contig_5_193	N	CE1(31-261)	N	N	N	1
contig_5_242	-	GT51(64-238)	GT51_e55	GT51	N	3
contig_5_266	5.4.99.11|3.2.1.20|3.2.1.10|3.2.1.-|3.2.1.70	GH13_31(28-371)	GH13_e122	GH13_31	N	3
contig_5_27	N	GH109(1-152)	N	N	N	1
contig_5_276	N	GH188(2-145)	N	N	N	1
contig_5_311	3.2.1.17|4.2.2.n1	GH23(1436-1547)	GH23_e335	GH23	N	3
contig_6_139	-	GH177(3-295)	GH109_e5	N	N	2
contig_6_170	3.2.1.99	GH43_4(33-359)	GH43_e38	GH43_4	N	3
contig_6_175	-	PL1_8(88-284)	PL1_e106	PL1_8	N	3
contig_6_198	3.2.1.73|3.2.1.6|3.2.1.8	GH16_21(38-238)	GH16_e14	GH16_21	N	3
contig_6_216	-	CE4(114-242)	CE4_e274	N	N	2
contig_6_226	3.2.1.78|3.2.1.-	GH26(30-348)	GH26_e0	GH26	N	3
contig_6_229	3.2.1.86|3.2.1.85|3.2.1.21	GH1(7-465)	GH1_e13	GH1	N	3
contig_6_243	3.2.1.86	GH4(6-185)	GH4_e24	GH4	N	3
contig_6_253	-	GT8(5-257)	GT8_e207	GT8	N	3
contig_6_268	-	GT2(8-218)	GT2	N	N	2
contig_6_282	N	GT119(19-384)	N	GT119	N	2
contig_6_290	3.2.1.26	GH32(33-338)	GH32_e57	GH32	N	3
contig_6_293	-	GT2(4-162)	GT2	GT2	N	3
contig_6_299	-	GT2(5-168)	GT2	GT2	N	3
contig_6_347	-	GT51(63-237)	GT51_e8	GT51	N	3
contig_6_50	3.2.1.65|3.2.1.64	GH32(64-368)	GH32_e117	GH32	N	3
contig_6_51	2.4.1.10	GH68_1(42-469)	GH68_e0	GH68_1	N	3
contig_6_62	-	GT4(254-406)	GT4_e3738	GT4	N	3
contig_6_65	-	GT4(251-402)	GT4_e2274	GT4	N	3
contig_8_107	N	GT119(18-374)	N	GT119	N	2
contig_8_144	N	GT119(2-337)	N	GT119	N	2
contig_8_145	-	GT28(188-350)	GT28_e1	GT28	N	3
contig_8_291	-	CE4(123-247)	CE4_e20	CE4	N	3
contig_8_378	3.2.1.37|3.2.1.55|3.2.1.-|-	GH43_11(3-301)+CBM91(331-532)	GH43_e285+CBM91_e24	CBM91+GH43_11	N	3
contig_8_396	1.14.99.53	AA10(28-203)	AA10_e1	AA10	N	3
contig_8_448	3.2.1.4|3.2.1.78|3.2.1.132	GH5_2(53-290)+CBM3(357-437)	GH5_e251+CBM3_e18	CBM3+GH5_2	N	3
contig_8_452	3.2.1.8|3.2.1.136	GH30_8(33-419)	GH30_e78	GH30_8	N	3
contig_8_453	3.2.1.55|3.2.1.8|3.2.1.-|3.2.1.37	GH43_16(38-360)+CBM6(382-511)	GH43_e261+CBM6_e6	CBM6+GH43_16	N	3
contig_8_88	-	CE4(73-195)	CE4_e129	N	N	2
contig_9_162	-	GT2(49-279)	GT2	GT2	N	3
contig_9_165	-	GH126(42-350)	GH126_e0	GH126	N	3
contig_9_18	-	GT1(95-391)	GT1_e274	GT1	N	3
contig_9_70	1.1.3.-	AA7(23-242)	AA7_e0	N	N	2
contig_14_155	-	N	CBM50_e1	CBM50	N	2
contig_17_95	-	N	CBM50_e1023	N	N	1
contig_1_116	-	N	GH23_e126	GH0	N	2
contig_1_142	-	N	CBM50_e826	CBM50	N	2
contig_1_437	-	N	CBM50_e833+CBM50_e1004+CBM50_e	CBM50	N	2
contig_25_39	-	N	GH23_e590	GH23	N	2
contig_25_45	-	N	CBM50_e375+CBM50_e520	CBM50	N	2
contig_25_69	-	N	CBM50_e817+CBM50_e1004+CBM50_e	CBM50	N	2
contig_1_115	-	N	CBM50_e932	CBM50	N	2
contig_1_432	-	N	CBM50_e520+CBM50_e438	CBM50	N	2
contig_8_23	-	N	CBM50_e55	N	N	1
contig_9_157	-	N	CBM50_e826	N	N	1
contig_19_37	-	N	GT4_e101	GT4	N	2
contig_1_8	-	N	CBM50_e77	N	N	1
contig_1_9	-	N	CBM50_e1022	CBM50	N	2
contig_1_19	N	N	N	GT4	N	1
contig_1_241	N	N	N	CBM50	N	1
contig_1_443	N	N	N	GH130_1	N	1
contig_2_4	N	N	N	CBM13	N	1
contig_3_37	N	N	N	GH13_31	N	1
contig_3_70	N	N	N	GT2	N	1
contig_4_4	N	N	N	GT4	N	1
contig_4_25	N	N	N	GH1	N	1
contig_4_29	N	N	N	CE4	N	1
contig_5_30	N	N	N	CBM50	N	1
contig_5_60	N	N	N	CE8	N	1
contig_5_142	N	N	N	CE4	N	1
contig_5_156	N	N	N	GH0	N	1
contig_5_198	N	N	N	GT1	N	1
contig_6_33	N	N	N	GH9	N	1
contig_6_53	N	N	N	CBM50	N	1
contig_6_89	N	N	N	GT22	N	1
contig_6_112	N	N	N	GH13_3	N	1
contig_6_176	N	N	N	CBM0+CBM16	N	1
contig_6_285	N	N	N	GH0	N	1
contig_6_305	N	N	N	GT0	N	1
contig_7_1	N	N	N	GT2	N	1
contig_7_2	N	N	N	GT1	N	1
contig_8_75	N	N	N	GT2	N	1
contig_8_77	N	N	N	GT2	N	1
contig_8_80	N	N	N	GT2	N	1
contig_8_164	N	N	N	GT0	N	1
contig_8_187	N	N	N	CBM50	N	1
contig_8_230	N	N	N	GT2	N	1
contig_8_278	N	N	N	CBM50+GH25	N	1
contig_8_284	N	N	N	GT2	N	1
contig_8_290	N	N	N	GH13_48	N	1
contig_8_302	N	N	N	GT2	N	1
contig_8_339	N	N	N	GT2	N	1
contig_8_340	N	N	N	GT2	N	1
contig_8_341	N	N	N	GT2	N	1
contig_8_342	N	N	N	GT2	N	1
contig_8_401	N	N	N	GH0	N	1
contig_8_454	N	N	N	GT2	N	1
contig_8_455	N	N	N	GT1	N	1
contig_8_456	N	N	N	GT2	N	1
contig_8_476	N	N	N	GT32	N	1
contig_8_480	N	N	N	GT2	N	1
contig_8_481	N	N	N	GT1	N	1
contig_9_164	N	N	N	GH126	N	1
contig_9_199	N	N	N	GH36	N	1
contig_9_243	N	N	N	GT2	N	1
contig_10_7	N	N	N	GT4	N	1
contig_10_29	N	N	N	GH4	N	1
contig_10_33	N	N	N	GT4	N	1
contig_10_60	N	N	N	GH13_3	N	1
contig_12_31	N	N	N	GH0	N	1
contig_12_32	N	N	N	GH4	N	1
contig_12_67	N	N	N	CBM50	N	1
contig_13_20	N	N	N	GT0	N	1
contig_13_22	N	N	N	GT0	N	1
contig_13_27	N	N	N	GT4	N	1
contig_13_76	N	N	N	GH13_23	N	1
contig_13_95	N	N	N	GH131	N	1
contig_13_192	N	N	N	GH13	N	1
contig_14_40	N	N	N	GH13_30	N	1
contig_14_69	N	N	N	GH28	N	1
contig_14_140	N	N	N	GT2	N	1
contig_14_146	N	N	N	GT2	N	1
contig_14_158	N	N	N	GH5_11	N	1
contig_15_38	N	N	N	GH0	N	1
contig_15_63	N	N	N	GT4	N	1
contig_15_116	N	N	N	GH28	N	1
contig_15_120	N	N	N	GT1	N	1
contig_15_277	N	N	N	GH101	N	1
contig_15_282	N	N	N	GT30	N	1
contig_15_294	N	N	N	GT2	N	1
contig_16_1	N	N	N	GT1	N	1
contig_16_3	N	N	N	GT2	N	1
contig_16_4	N	N	N	GT2	N	1
contig_16_6	N	N	N	GT2	N	1
contig_16_19	N	N	N	GH0	N	1
contig_16_46	N	N	N	CBM6	N	1
contig_16_118	N	N	N	GT1	N	1
contig_17_32	N	N	N	GH24	N	1
contig_17_44	N	N	N	CE8	N	1
contig_17_55	N	N	N	GH1	N	1
contig_17_178	N	N	N	GT2	N	1
contig_17_181	N	N	N	GT0	N	1
contig_19_41	N	N	N	GT2	N	1
contig_19_49	N	N	N	GT0	N	1
contig_19_53	N	N	N	GT2	N	1
contig_19_111	N	N	N	GT2	N	1
contig_19_166	N	N	N	CBM48+GH13_8	N	1
contig_19_175	N	N	N	GH13_11	N	1
contig_19_186	N	N	N	GH23	N	1
contig_22_1	N	N	N	GT119	N	1
contig_22_14	N	N	N	GT50	N	1
contig_22_45	N	N	N	GH84	N	1
contig_24_13	N	N	N	GH38	N	1
contig_25_1	N	N	N	GT1	N	1
contig_25_58	N	N	N	GH92	N	1
contig_25_65	N	N	N	GT2	N	1
contig_26_2	N	N	N	GT2	N	1
contig_27_1	N	N	N	GT2	N	1
contig_28_1	N	N	N	GT1	N	1
contig_29_7	N	N	N	CBM50	N	1
contig_29_12	N	N	N	GH1	N	1
contig_30_1	N	N	N	GT0	N	1
contig_30_4	N	N	N	GT2	N	1
contig_34_1	N	N	N	GT1	N	1
contig_34_2	N	N	N	GT2	N	1
contig_36_18	N	N	N	GH0	N	1
contig_36_21	N	N	N	GH113	N	1
contig_36_23	N	N	N	GH23	N	1
contig_42_25	N	N	N	GH113	N	1
contig_42_26	N	N	N	GH23	N	1
contig_43_1	N	N	N	GT2	N	1
contig_43_2	N	N	N	GT1	N	1
contig_47_1	N	N	N	GT1	N	1
contig_47_2	N	N	N	GT1	N	1
contig_51_1	N	N	N	GT1	N	1

S111.4_overview

Gene ID	EC#	HMMER	dbCAN_sub	DIAMOND	Signalp	#ofTools
contig_10_113	3.2.1.1	GH13_28(58-339)+CBM26(565-642)	GH13_e34+CBM26_e0	CBM26+GH13_28	N	3
contig_10_12	3.2.1.92	GH171(57-414)	GH171_e0	GH171	N	3
contig_10_127	3.1.1.41|3.1.1.72	CE7(4-312)	CE7_e11	CE7	N	3
contig_10_13	3.2.1.52	GH3(108-343)	GH3_e28	GH3	N	3
contig_10_144	3.2.1.86|3.2.1.38|3.2.1.85|3.2.1.21|3.2.1.-	GH1(8-475)	GH1_e115	GH1	N	3
contig_10_7	N	CE1(14-241)	N	N	N	1
contig_10_95	5.4.99.11|3.2.1.20|3.2.1.10|3.2.1.-|3.2.1.70	GH13_31(28-375)	GH13_e122	GH13_31	N	3
contig_11_80	-	GH126(42-350)	GH126_e0	GH126	N	3
contig_11_83	-	GT2(49-279)	GT2	GT2	N	3
contig_12_75	-	CE4(52-178)	CE4_e14	CE4	N	3
contig_16_10	3.2.1.-	GH18(126-404)	CBM50_e699+CBM50_e699+GH18_e157	CBM50+GH18	N	3
contig_1_123	4.2.2.2	PL1_6(135-348)	PL1_e108	PL1_6	N	3
contig_1_133	-	GT2(5-126)	GT2	GT2	N	3
contig_1_140	-	GT2(4-129)	GT2	GT2	N	3
contig_1_16	-	GT2(7-168)	GT2	GT2	N	3
contig_1_226	N	AA1(48-499)	N	N	N	1
contig_1_47	3.2.1.122	GH4(8-185)	GH4_e4	GH4	N	3
contig_1_67	3.5.1.104	CE4(59-185)	CE4_e23	CE4	N	3
contig_1_89	3.2.1.93	GH13_29(30-376)	GH13_e1	GH13_29	N	3
contig_2_111	2.4.1.157	GT28(204-354)	GT28_e89	GT28	N	3
contig_2_133	-	GT4(318-470)	GT4_e3580	GT4	N	3
contig_2_142	-	GH73(13-149)	GH73_e27	GH73	N	3
contig_2_207	2.4.1.129	GT51(87-263)	GT51_e113	GT51	N	3
contig_2_221	2.4.1.-	GT4(196-344)	GT4_e1442	GT4	N	3
contig_2_222	-	CE14(8-113)	CE14_e0	CE14	N	3
contig_2_39	-	GT1(9-393)	GT1_e505	GT1	N	3
contig_2_44	-	CE14(6-124)	CE14_e39	N	N	2
contig_2_562	N	CE12(30-200)	N	N	N	1
contig_2_563	-	CE4(42-163)	CE4_e317	CE4	N	3
contig_2_567	-	CE6(55-146)	CE6_e1	CE0	N	3
contig_2_59	-	GH30_3(81-359)	GH30_e56	GH30_3	N	3
contig_2_60	3.2.1.86|3.2.1.23|3.2.1.21	GH1(3-476)	GH1_e0	GH1	N	3
contig_2_659	-	CBM50(4-48)	CBM50_e257	CBM50	N	3
contig_2_732	3.2.1.55	GH51_1(7-490)	GH51_e19	GH51_1	N	3
contig_2_750	-	AA4(11-236)	AA4_e1	N	N	2
contig_2_753	3.2.1.55	GH51_1(7-496)	GH51_e0	GH51_1	N	3
contig_2_762	3.2.1.99	GH43_5(41-319)	GH43_e56	GH43_5	N	3
contig_2_84	-	GT2(6-168)	GT2	GT2	N	3
contig_2_902	3.2.1.22	GH4(3-180)	GH4_e17	GH4	N	3
contig_3_147	-	GH188(3-160)	GH109_e5	N	N	2
contig_3_183	3.2.1.99	GH43_4(33-359)	GH43_e38	GH43_4	N	3
contig_3_188	-	PL1_8(88-284)	PL1_e106	PL1_8	N	3
contig_3_192	3.2.1.73|3.2.1.6|3.2.1.8	GH16_21(38-238)	GH16_e14	GH16_21	N	3
contig_3_210	-	CE4(114-242)	CE4_e274	N	N	2
contig_3_220	3.2.1.78|3.2.1.-	GH26(30-348)	GH26_e0	GH26	N	3
contig_3_223	3.2.1.86|3.2.1.85|3.2.1.21	GH1(7-465)	GH1_e13	GH1	N	3
contig_3_237	3.2.1.86	GH4(6-185)	GH4_e24	GH4	N	3
contig_3_248	-	GT8(5-257)	GT8_e207	GT8	N	3
contig_3_263	-	GT2(8-218)	GT2	N	N	2
contig_3_277	N	GT119(19-384)	N	GT119	N	2
contig_3_285	3.2.1.26	GH32(33-338)	GH32_e57	GH32	N	3
contig_3_291	-	GT2(4-162)	GT2	GT2	N	3
contig_3_297	-	GT2(5-168)	GT2	GT2	N	3
contig_3_349	-	GT51(63-237)	GT51_e8	GT51	N	3
contig_3_432	3.2.1.8	GH11(32-211)	GH11_e15	GH11	N	3
contig_3_441	N	GT119(17-362)	N	GT119	N	2
contig_3_519	-	GT2(4-133)	GT2	GT2	N	3
contig_3_52	3.2.1.65|3.2.1.64	GH32(64-369)	GH32_e117	GH32	N	3
contig_3_524	3.2.1.-|3.2.1.17	GH73(734-860)	GH73_e162	GH73	N	3
contig_3_526	2.4.1.-	GT26(57-224)	GT26_e19	GT26	N	3
contig_3_528	-	GT2(39-168)	GT2	GT2	N	3
contig_3_53	2.4.1.10	GH68_1(42-469)	GH68_e0	GH68_1	N	3
contig_3_540	-	GT4(215-345)	GT4_e23	GT4	N	3
contig_3_544	-	GT2(9-123)	GT2	GT2	N	3
contig_3_575	-	PL9_2(33-390)	PL9_e18	PL9_2	N	3
contig_3_64	-	GT4(253-406)	GT4_e3738	GT4	N	3
contig_3_67	-	GT4(251-402)	GT4_e2274	GT4	N	3
contig_3_68	-	GT83(8-225)	GT83_e60	N	N	2
contig_4_29	3.2.1.55|3.2.1.8|3.2.1.-|3.2.1.37	GH43_16(37-359)+CBM6(381-510)	GH43_e261+CBM6_e6	CBM6+GH43_16	N	3
contig_4_30	3.2.1.8|3.2.1.136	GH30_8(33-419)	GH30_e78	GH30_8	N	3
contig_4_34	3.2.1.4|3.2.1.78|3.2.1.132	GH5_2(53-290)+CBM3(357-439)	GH5_e251+CBM3_e18	CBM3+GH5_2	N	3
contig_4_90	1.14.99.53	AA10(28-203)	AA10_e1	AA10	N	3
contig_5_105	-	CE4(123-247)	CE4_e20	CE4	N	3
contig_5_15	3.2.1.37|3.2.1.55|3.2.1.-|-	GH43_11(3-301)+CBM91(331-532)	GH43_e285+CBM91_e24	CBM91+GH43_11	N	3
contig_5_250	-	GT28(188-350)	GT28_e1	GT28	N	3
contig_5_251	N	GT119(2-337)	N	GT119	N	2
contig_5_287	N	GT119(18-374)	N	GT119	N	2
contig_5_309	-	CE4(70-192)	CE4_e129	N	N	2
contig_5_448	-	GH18(171-401)	CBM50_e96+CBM50_e96+GH18_e174	CBM50+GH18	N	3
contig_5_458	-	GT2(11-95)	GT2	GT2	N	3
contig_5_486	-	GT2(8-170)	GT2	GT2	N	3
contig_5_487	-	GT83(10-262)	GT83_e35	N	N	2
contig_5_494	-	CBM50(161-203)	CBM50_e826	CBM50	N	3
contig_5_530	2.4.1.-	GT1(175-387)	GT1_e225	GT1	N	3
contig_5_543	3.2.1.85	GH1(3-463)	GH1_e65	GH1	N	3
contig_5_549	-	GH53(40-341)	GH53_e0	GH53	N	3
contig_5_564	N	CE1(15-237)	N	N	N	1
contig_5_598	3.2.1.17|4.2.2.n1	GH23(123-226)	GH23_e335	GH23	N	3
contig_5_663	N	GH177(5-308)	N	N	N	1
contig_5_727	2.4.1.129	GT51(55-229)	GT51_e20	GT51	N	3
contig_5_773	-	CE4(81-207)	CE4_e25	CE4	N	3
contig_5_832	-	AA6(2-169)	AA6_e2	N	N	2
contig_5_861	1.1.3.-	AA7(26-248)	AA7_e0	N	N	2
contig_7_2	1.1.3.-	AA7(23-243)	AA7_e0	N	N	2
contig_7_53	-	GT1(115-391)	GT1_e274	GT1	N	3
contig_8_162	N	GH109(1-153)	N	N	N	1
contig_8_21	3.5.1.25	CE9(8-384)	CE9_e51	CE9	N	3
contig_8_256	3.2.1.132	GH46(38-262)	GH46_e0	GH46	N	3
contig_8_326	N	CE1(31-261)	N	N	N	1
contig_8_375	-	GT51(64-238)	GT51_e55	GT51	N	3
contig_8_399	5.4.99.11|3.2.1.20|3.2.1.10|3.2.1.-|3.2.1.70	GH13_31(28-371)	GH13_e122	GH13_31	N	3
contig_8_409	N	GH188(2-145)	N	N	N	1
contig_8_415	3.2.1.-	GH73(58-193)	GH73_e25	GH73	N	3
contig_8_54	3.2.1.54|3.2.1.133|3.2.1.135	CBM34(6-126)+GH13_20(175-468)	CBM34_e0+GH13_e225	CBM34+GH13_20	N	3
contig_8_59	2.4.1.8	GH65(319-687)	GH65_e0	GH65	N	3
contig_8_60	5.4.99.11|3.2.1.20|3.2.1.10|3.2.1.-|3.2.1.70	GH13_31(27-377)	GH13_e122	GH13_31	N	3
contig_8_65	3.2.1.26	GH32(27-331)	GH32_e0	GH32	N	3
contig_8_83	-	GT4(193-345)	GT4_e2316	GT4	N	3
contig_8_84	-	GT2(10-163)	GT2	GT2	N	3
contig_8_85	-	GT4(197-305)	GT4_e1131	GT4	N	3
contig_8_86	N	GT122(39-352)	N	GT122	N	2
contig_8_87	-	GT2(7-147)	GT2	GT2	N	3
contig_8_89	-	GT2(7-132)	GT2	GT2	N	3
contig_11_88	-	N	CBM50_e826	N	N	1
contig_2_14	-	N	CBM50_e375+CBM50_e520	CBM50	N	2
contig_2_144	-	N	GH23_e126	GH0	N	2
contig_2_38	-	N	CBM50_e817+CBM50_e1012+CBM50_e1	CBM50	N	2
contig_2_8	-	N	GH23_e590	GH23	N	2
contig_5_512	-	N	CBM50_e826	CBM50	N	2
contig_5_794	-	N	CBM50_e520+CBM50_e438	CBM50	N	2
contig_5_799	-	N	CBM50_e833+CBM50_e1004+CBM50_e4	CBM50	N	2
contig_2_277	-	N	CBM50_e1023	N	N	1
contig_2_687	-	N	CBM50_e1	CBM50	N	2
contig_3_545	-	N	GT4_e101	GT4	N	2
contig_5_374	-	N	CBM50_e55	N	N	1
contig_1_45	N	N	N	GH4	N	1
contig_1_84	N	N	N	CBM50	N	1
contig_1_139	N	N	N	GT0	N	1
contig_1_141	N	N	N	GT0	N	1
contig_1_145	N	N	N	GT4	N	1
contig_1_197	N	N	N	GH13_23	N	1
contig_1_220	N	N	N	GH131	N	1
contig_1_246	N	N	N	GH13	N	1
contig_2_27	N	N	N	GH92	N	1
contig_2_34	N	N	N	GT2	N	1
contig_2_141	N	N	N	GH0	N	1
contig_2_215	N	N	N	GH24	N	1
contig_2_237	N	N	N	GH1	N	1
contig_2_360	N	N	N	GT2	N	1
contig_2_363	N	N	N	GT2	N	1
contig_2_379	N	N	N	GT30	N	1
contig_2_384	N	N	N	GH101	N	1
contig_2_539	N	N	N	GT1	N	1
contig_2_543	N	N	N	GH28	N	1
contig_2_593	N	N	N	GT4	N	1
contig_2_684	N	N	N	GH5_11	N	1
contig_2_696	N	N	N	GT2	N	1
contig_2_702	N	N	N	GT2	N	1
contig_2_774	N	N	N	GH28	N	1
contig_2_806	N	N	N	GH13_30	N	1
contig_2_870	N	N	N	GT2	N	1
contig_2_879	N	N	N	GT4	N	1
contig_2_901	N	N	N	GH4	N	1
contig_2_905	N	N	N	GT4	N	1
contig_2_929	N	N	N	GH13_3	N	1
contig_3_32	N	N	N	GH9	N	1
contig_3_55	N	N	N	CBM50	N	1
contig_3_92	N	N	N	GT22	N	1
contig_3_120	N	N	N	GH13_3	N	1
contig_3_280	N	N	N	GH0	N	1
contig_3_303	N	N	N	GT0	N	1
contig_3_393	N	N	N	GH23	N	1
contig_3_404	N	N	N	GH13_11	N	1
contig_3_413	N	N	N	CBM48+GH13_8	N	1
contig_3_467	N	N	N	GT2	N	1
contig_3_529	N	N	N	GT2	N	1
contig_3_533	N	N	N	GT0	N	1
contig_3_541	N	N	N	GT2	N	1
contig_3_582	N	N	N	GT1	N	1
contig_3_583	N	N	N	GT2	N	1
contig_4_1	N	N	N	GT1	N	1
contig_4_2	N	N	N	GT2	N	1
contig_4_26	N	N	N	GT2	N	1
contig_4_27	N	N	N	GT1	N	1
contig_4_28	N	N	N	GT2	N	1
contig_4_84	N	N	N	GH0	N	1
contig_5_53	N	N	N	GT2	N	1
contig_5_55	N	N	N	GT2	N	1
contig_5_56	N	N	N	GT2	N	1
contig_5_94	N	N	N	GT2	N	1
contig_5_106	N	N	N	GH13_48	N	1
contig_5_112	N	N	N	GT2	N	1
contig_5_118	N	N	N	CBM50+GH25	N	1
contig_5_166	N	N	N	GT2	N	1
contig_5_209	N	N	N	CBM50	N	1
contig_5_232	N	N	N	GT0	N	1
contig_5_317	N	N	N	GT2	N	1
contig_5_320	N	N	N	GT2	N	1
contig_5_322	N	N	N	GT2	N	1
contig_5_410	N	N	N	GT4	N	1
contig_5_602	N	N	N	CBM50	N	1
contig_5_805	N	N	N	GH130_1	N	1
contig_5_817	N	N	N	CBM50	N	1
contig_5_822	N	N	N	GH1	N	1
contig_5_854	N	N	N	GH38	N	1
contig_8_55	N	N	N	CBM50+CE4	N	1
contig_8_56	N	N	N	GH13_48	N	1
contig_8_90	N	N	N	GT2	N	1
contig_8_121	N	N	N	GH13_31	N	1
contig_8_165	N	N	N	CBM50	N	1
contig_8_195	N	N	N	CE8	N	1
contig_8_277	N	N	N	CE4	N	1
contig_8_289	N	N	N	GH0	N	1
contig_8_331	N	N	N	GT1	N	1
contig_10_56	N	N	N	GH0	N	1
contig_10_78	N	N	N	CBM6	N	1
contig_10_150	N	N	N	GT1	N	1
contig_11_2	N	N	N	GT2	N	1
contig_11_46	N	N	N	GH36	N	1
contig_11_81	N	N	N	GH126	N	1
contig_12_4	N	N	N	GT4	N	1
contig_12_25	N	N	N	GH1	N	1
contig_12_29	N	N	N	CE4	N	1
contig_13_1	N	N	N	GT119	N	1
contig_13_14	N	N	N	GT50	N	1
contig_13_45	N	N	N	GH84	N	1
contig_14_2	N	N	N	GT1	N	1
contig_15_11	N	N	N	GH28	N	1
contig_16_4	N	N	N	CBM13	N	1
contig_22_1	N	N	N	GT1	N	1
contig_22_2	N	N	N	GT1	N	1
contig_25_1	N	N	N	GT1	N	1
contig_25_2	N	N	N	GT1	N	1

S106.1b_overview

Gene ID	EC#	HMMER	dbCAN_sub	DIAMOND	Signalp	#ofTools
contig_11_153	3.2.1.52	GH3(108-343)	GH3_e28	GH3	N	3
contig_11_154	3.2.1.92	GH171(57-414)	GH171_e0	GH171	N	3
contig_11_159	N	CE1(14-241)	N	N	N	1
contig_11_25	3.1.1.41|3.1.1.72	CE7(4-312)	CE7_e11	CE7	N	3
contig_11_38	3.2.1.1	GH13_28(58-339)+CBM26(565-642)	GH13_e34+CBM26_e0	CBM26+GH13_28	N	3
contig_11_58	5.4.99.11|3.2.1.20|3.2.1.10|3.2.1.-|3.2.1.70	GH13_31(28-375)	GH13_e122	GH13_31	N	3
contig_12_109	1.1.3.-	AA7(22-243)	AA7_e0	N	N	2
contig_12_11	-	GH126(42-350)	GH126_e0	GH126	N	3
contig_12_14	-	GT2(49-279)	GT2	GT2	N	3
contig_12_149	-	GT1(49-391)	GT1_e274	GT1	N	3
contig_15_8	3.2.1.-	GH18(127-404)	CBM50_e699+CBM50_e699+GH18_e157	CBM50+GH18	N	3
contig_1_1001	3.2.1.-	GH73(58-193)	GH73_e25	GH73	N	3
contig_1_144	-	GH188(3-160)	GH109_e5	N	N	2
contig_1_175	3.2.1.99	GH43_4(33-359)	GH43_e38	GH43_4	N	3
contig_1_180	-	PL1_8(88-284)	PL1_e106	PL1_8	N	3
contig_1_193	3.2.1.73|3.2.1.6|3.2.1.8	GH16_21(38-238)	GH16_e14	GH16_21	N	3
contig_1_210	-	CE4(114-242)	CE4_e274	N	N	2
contig_1_220	3.2.1.78|3.2.1.-	GH26(30-348)	GH26_e0	GH26	N	3
contig_1_223	3.2.1.86|3.2.1.85|3.2.1.21	GH1(7-465)	GH1_e13	GH1	N	3
contig_1_237	3.2.1.86	GH4(6-185)	GH4_e24	GH4	N	3
contig_1_246	-	GT8(5-257)	GT8_e207	GT8	N	3
contig_1_261	-	GT2(8-218)	GT2	N	N	2
contig_1_275	N	GT119(19-384)	N	GT119	N	2
contig_1_282	3.2.1.26	GH32(33-338)	GH32_e57	GH32	N	3
contig_1_288	-	GT2(4-162)	GT2	GT2	N	3
contig_1_294	-	GT2(5-168)	GT2	GT2	N	3
contig_1_348	-	GT51(63-237)	GT51_e8	GT51	N	3
contig_1_434	3.2.1.8	GH11(32-211)	GH11_e15	GH11	N	3
contig_1_443	N	GT119(17-362)	N	GT119	N	2
contig_1_520	-	GT2(4-169)	GT2	GT2	N	3
contig_1_525	3.2.1.-|3.2.1.17	GH73(734-861)	GH73_e162	GH73	N	3
contig_1_527	2.4.1.-	GT26(57-224)	GT26_e19	GT26	N	3
contig_1_529	-	GT2(39-168)	GT2	GT2	N	3
contig_1_541	-	GT4(215-346)	GT4_e23	GT4	N	3
contig_1_545	-	GT2(9-123)	GT2	GT2	N	3
contig_1_56	3.2.1.65|3.2.1.64	GH32(45-350)	GH32_e117	GH32	N	3
contig_1_57	2.4.1.10	GH68_1(42-469)	GH68_e0	GH68_1	N	3
contig_1_576	-	PL9_2(33-390)	PL9_e18	PL9_2	N	3
contig_1_596	3.5.1.25	CE9(9-384)	CE9_e51	CE9	N	3
contig_1_630	3.2.1.54|3.2.1.133|3.2.1.135	CBM34(6-126)+GH13_20(175-468)	CBM34_e0+GH13_e225	CBM34+GH13_20	N	3
contig_1_635	2.4.1.8	GH65(319-687)	GH65_e0	GH65	N	3
contig_1_636	5.4.99.11|3.2.1.20|3.2.1.10|3.2.1.-|3.2.1.70	GH13_31(27-377)	GH13_e122	GH13_31	N	3
contig_1_641	3.2.1.26	GH32(27-331)	GH32_e0	GH32	N	3
contig_1_659	-	GT4(193-345)	GT4_e2316	GT4	N	3
contig_1_660	-	GT2(10-163)	GT2	GT2	N	3
contig_1_661	-	GT4(197-305)	GT4_e1131	GT4	N	3
contig_1_662	N	GT122(39-352)	N	GT122	N	2
contig_1_663	-	GT2(7-164)	GT2	GT2	N	3
contig_1_665	-	GT2(7-132)	GT2	GT2	N	3
contig_1_68	-	GT4(253-405)	GT4_e3738	GT4	N	3
contig_1_708	-	CE14(4-114)	CE14_e46	N	N	2
contig_1_71	-	GT4(252-402)	GT4_e2274	GT4	N	3
contig_1_752	N	GH109(1-152)	N	N	N	1
contig_1_849	3.2.1.132	GH46(38-262)	GH46_e0	GH46	N	3
contig_1_912	N	CE1(31-261)	N	N	N	1
contig_1_961	-	GT51(64-238)	GT51_e55	GT51	N	3
contig_1_985	5.4.99.11|3.2.1.20|3.2.1.10|3.2.1.-|3.2.1.70	GH13_31(28-371)	GH13_e122	GH13_31	N	3
contig_1_995	N	GH188(2-145)	N	N	N	1
contig_2_172	2.4.1.129	GT51(55-229)	GT51_e20	GT51	N	3
contig_2_219	-	CE4(81-207)	CE4_e25	CE4	N	3
contig_2_278	-	AA6(2-169)	AA6_e2	N	N	2
contig_2_309	1.1.3.-	AA7(21-242)	AA7_e0	N	N	2
contig_2_44	3.2.1.17|4.2.2.n1	GH23(123-226)	GH23_e335	GH23	N	3
contig_2_8	N	CE1(15-237)	N	N	N	1
contig_3_115	3.2.1.37|3.2.1.55|3.2.1.-|-	GH43_11(3-301)+CBM91(331-532)	GH43_e285+CBM91_e24	CBM91+GH43_11	N	3
contig_3_201	-	CE4(123-247)	CE4_e20	CE4	N	3
contig_3_29	3.2.1.55|3.2.1.8|3.2.1.-|3.2.1.37	GH43_16(38-360)+CBM6(382-511)	GH43_e261+CBM6_e6	CBM6+GH43_16	N	3
contig_3_30	3.2.1.8|3.2.1.136	GH30_8(33-419)	GH30_e78	GH30_8	N	3
contig_3_34	3.2.1.4|3.2.1.78|3.2.1.132	GH5_2(53-290)+CBM3(357-437)	GH5_e251+CBM3_e18	CBM3+GH5_2	N	3
contig_3_346	-	GT28(188-350)	GT28_e1	GT28	N	3
contig_3_347	N	GT119(2-337)	N	GT119	N	2
contig_3_384	N	GT119(18-374)	N	GT119	N	2
contig_3_406	-	CE4(73-195)	CE4_e129	N	N	2
contig_3_547	-	GH18(171-401)	CBM50_e96+CBM50_e96+GH18_e174	CBM50+GH18	N	3
contig_3_557	-	GT2(11-95)	GT2	GT2	N	3
contig_3_585	-	GT2(8-170)	GT2	GT2	N	3
contig_3_586	-	GT83(10-262)	GT83_e35	N	N	2
contig_3_593	-	CBM50(161-203)	CBM50_e826	CBM50	N	3
contig_3_652	2.4.1.-	GT1(175-387)	GT1_e225	GT1	N	3
contig_3_665	3.2.1.85	GH1(3-463)	GH1_e65	GH1	N	3
contig_3_671	-	GH53(40-341)	GH53_e0	GH53	N	3
contig_3_90	1.14.99.53	AA10(28-203)	AA10_e1	AA10	N	3
contig_4_108	3.5.1.104	CE4(59-185)	CE4_e23	CE4	N	3
contig_4_127	3.2.1.122	GH4(8-185)	GH4_e4	GH4	N	3
contig_4_158	-	GT2(7-168)	GT2	GT2	N	3
contig_4_35	-	GT2(4-129)	GT2	GT2	N	3
contig_4_42	-	GT2(5-126)	GT2	GT2	N	3
contig_4_52	4.2.2.2	PL1_6(135-348)	PL1_e108	PL1_6	N	3
contig_4_86	3.2.1.93	GH13_29(30-376)	GH13_e1	GH13_29	N	3
contig_5_1	-	CE4(52-178)	CE4_e14	CE4	N	3
contig_6_114	-	GT2(6-168)	GT2	GT2	N	3
contig_6_160	2.4.1.157	GT28(204-354)	GT28_e89	GT28	N	3
contig_6_203	2.4.1.129	GT51(87-263)	GT51_e113	GT51	N	3
contig_6_217	2.4.1.-	GT4(196-344)	GT4_e1442	GT4	N	3
contig_6_218	-	CE14(8-113)	CE14_e0	CE14	N	3
contig_6_70	-	GT1(9-393)	GT1_e505	GT1	N	3
contig_6_75	-	CE14(6-124)	CE14_e39	N	N	2
contig_6_89	-	GH30_3(82-488)	GH30_e56	GH30_3	N	3
contig_6_90	3.2.1.86|3.2.1.23|3.2.1.21	GH1(3-476)	GH1_e0	GH1	N	3
contig_7_133	-	GH73(13-149)	GH73_e27	GH73	N	3
contig_7_183	-	CE6(55-146)	CE6_e1	CE0	N	3
contig_7_191	-	CE4(42-163)	CE4_e317	CE4	N	3
contig_7_192	N	CE12(30-200)	N	N	N	1
contig_7_29	-	CBM50(4-48)	CBM50_e257	CBM50	N	3
contig_8_198	3.2.1.22	GH4(3-180)	GH4_e17	GH4	N	3
contig_8_40	3.2.1.55	GH51_1(7-305)	GH51_e19	GH51_1	N	3
contig_8_58	-	AA4(11-237)	AA4_e1	N	N	2
contig_8_61	3.2.1.55	GH51_1(7-496)	GH51_e0	GH51_1	N	3
contig_8_70	3.2.1.99	GH43_5(41-319)	GH43_e56	GH43_5	N	3
contig_9_59	N	AA1(48-499)	N	N	N	1
contig_3_607	-	N	GH23_e126	GH0	N	2
contig_3_634	-	N	CBM50_e826	CBM50	N	2
contig_7_131	-	N	GH23_e126	GH0	N	2
contig_1_546	-	N	GT4_e101	GT4	N	2
contig_2_240	-	N	CBM50_e520+CBM50_e438	CBM50	N	2
contig_2_245	-	N	CBM50_e833+CBM50_e1004+CBM50_e43	CBM50	N	2
contig_3_470	-	N	CBM50_e55	N	N	1
contig_3_606	-	N	CBM50_e932	CBM50	N	2
contig_6_273	-	N	CBM50_e1023	N	N	1
contig_6_39	-	N	GH23_e590	GH23	N	2
contig_6_45	-	N	CBM50_e375+CBM50_e520	CBM50	N	2
contig_6_69	-	N	CBM50_e817+CBM50_e1004+CBM50_e1	CBM50	N	2
contig_7_1	-	N	CBM50_e1	CBM50	N	2
contig_1_34	N	N	N	GH9	N	1
contig_1_59	N	N	N	CBM50	N	1
contig_1_95	N	N	N	GT22	N	1
contig_1_118	N	N	N	GH13_3	N	1
contig_1_181	N	N	N	CBM0+CBM16	N	1
contig_1_278	N	N	N	GH0	N	1
contig_1_300	N	N	N	GT0	N	1
contig_1_395	N	N	N	GH23	N	1
contig_1_406	N	N	N	GH13_11	N	1
contig_1_415	N	N	N	CBM48+GH13_8	N	1
contig_1_469	N	N	N	GT2	N	1
contig_1_530	N	N	N	GT2	N	1
contig_1_534	N	N	N	GT0	N	1
contig_1_542	N	N	N	GT2	N	1
contig_1_631	N	N	N	CBM50+CE4	N	1
contig_1_632	N	N	N	GH13_48	N	1
contig_1_666	N	N	N	GT2	N	1
contig_1_700	N	N	N	GH13_31	N	1
contig_1_755	N	N	N	CBM50	N	1
contig_1_785	N	N	N	CE8	N	1
contig_1_864	N	N	N	CE4	N	1
contig_1_875	N	N	N	GH0	N	1
contig_1_917	N	N	N	GT1	N	1
contig_2_48	N	N	N	CBM50	N	1
contig_2_251	N	N	N	GH130_1	N	1
contig_2_263	N	N	N	CBM50	N	1
contig_2_268	N	N	N	GH1	N	1
contig_2_302	N	N	N	GH38	N	1
contig_3_1	N	N	N	GT1	N	1
contig_3_2	N	N	N	GT2	N	1
contig_3_26	N	N	N	GT2	N	1
contig_3_27	N	N	N	GT1	N	1
contig_3_28	N	N	N	GT2	N	1
contig_3_85	N	N	N	GH0	N	1
contig_3_153	N	N	N	GT2	N	1
contig_3_155	N	N	N	GT2	N	1
contig_3_156	N	N	N	GT2	N	1
contig_3_190	N	N	N	GT2	N	1
contig_3_202	N	N	N	GH13_48	N	1
contig_3_208	N	N	N	GT2	N	1
contig_3_214	N	N	N	CBM50+GH25	N	1
contig_3_262	N	N	N	GT2	N	1
contig_3_305	N	N	N	CBM50	N	1
contig_3_328	N	N	N	GT0	N	1
contig_3_414	N	N	N	GT2	N	1
contig_3_417	N	N	N	GT2	N	1
contig_3_419	N	N	N	GT2	N	1
contig_3_510	N	N	N	GT4	N	1
contig_4_19	N	N	N	GT4	N	1
contig_4_34	N	N	N	GT0	N	1
contig_4_36	N	N	N	GT0	N	1
contig_4_91	N	N	N	CBM50	N	1
contig_4_129	N	N	N	GH4	N	1
contig_5_47	N	N	N	CE4	N	1
contig_5_51	N	N	N	GH1	N	1
contig_5_72	N	N	N	GT4	N	1
contig_6_1	N	N	N	GT2	N	1
contig_6_58	N	N	N	GH92	N	1
contig_6_65	N	N	N	GT2	N	1
contig_6_211	N	N	N	GH24	N	1
contig_6_233	N	N	N	GH1	N	1
contig_6_357	N	N	N	GT2	N	1
contig_6_360	N	N	N	GT0	N	1
contig_7_4	N	N	N	GH5_11	N	1
contig_7_134	N	N	N	GH0	N	1
contig_7_159	N	N	N	GT4	N	1
contig_7_211	N	N	N	GH28	N	1
contig_7_215	N	N	N	GT1	N	1
contig_7_370	N	N	N	GH101	N	1
contig_7_375	N	N	N	GT30	N	1
contig_7_388	N	N	N	GT2	N	1
contig_8_1	N	N	N	CBM50	N	1
contig_8_10	N	N	N	GT2	N	1
contig_8_16	N	N	N	GT2	N	1
contig_8_82	N	N	N	GH28	N	1
contig_8_111	N	N	N	GH13_30	N	1
contig_8_168	N	N	N	GT2	N	1
contig_8_175	N	N	N	GT4	N	1
contig_8_197	N	N	N	GH4	N	1
contig_8_201	N	N	N	GT4	N	1
contig_8_228	N	N	N	GH13_3	N	1
contig_9_36	N	N	N	GH13_23	N	1
contig_9_54	N	N	N	GH131	N	1
contig_9_82	N	N	N	GH13	N	1
contig_10_1	N	N	N	GT119	N	1
contig_10_14	N	N	N	GT50	N	1
contig_10_45	N	N	N	GH84	N	1
contig_11_1	N	N	N	GT1	N	1
contig_11_79	N	N	N	CBM6	N	1
contig_11_106	N	N	N	GH0	N	1
contig_12_12	N	N	N	GH126	N	1
contig_14_24	N	N	N	GH36	N	1
contig_14_68	N	N	N	GT2	N	1
contig_15_14	N	N	N	CBM13	N	1
contig_16_1	N	N	N	GT1	N	1
contig_17_1	N	N	N	GT2	N	1
contig_17_2	N	N	N	GT1	N	1
contig_17_3	N	N	N	GT1	N	1
contig_17_4	N	N	N	GT1	N	1
contig_19_1	N	N	N	GT0	N	1
contig_19_4	N	N	N	GT2	N	1
contig_21_1	N	N	N	GT1	N	1

N20.3_overview

Gene ID	EC#	HMMER	dbCAN_sub	DIAMOND	Signalp	#ofTools
contig_13_8	3.2.1.-	GH18(127-404)	CBM50_e699+CBM50_e699+GH18_e157	CBM50+GH18	N	3
contig_1_1004	3.2.1.-	GH73(58-193)	GH73_e25	GH73	N	3
contig_1_145	N	GH177(3-295)	N	N	N	1
contig_1_176	3.2.1.99	GH43_4(33-359)	GH43_e38	GH43_4	N	3
contig_1_181	-	PL1_8(88-284)	PL1_e106	PL1_8	N	3
contig_1_199	3.2.1.73|3.2.1.6|3.2.1.8	GH16_21(38-238)	GH16_e14	GH16_21	N	3
contig_1_216	-	CE4(114-242)	CE4_e274	N	N	2
contig_1_226	3.2.1.78|3.2.1.-	GH26(30-348)	GH26_e0	GH26	N	3
contig_1_229	3.2.1.86|3.2.1.85|3.2.1.21	GH1(7-465)	GH1_e13	GH1	N	3
contig_1_243	3.2.1.86	GH4(6-185)	GH4_e24	GH4	N	3
contig_1_254	-	GT8(5-257)	GT8_e207	GT8	N	3
contig_1_269	-	GT2(8-218)	GT2	N	N	2
contig_1_283	N	GT119(19-384)	N	GT119	N	2
contig_1_290	3.2.1.26	GH32(33-338)	GH32_e57	GH32	N	3
contig_1_296	-	GT2(4-162)	GT2	GT2	N	3
contig_1_302	-	GT2(5-168)	GT2	GT2	N	3
contig_1_359	-	GT51(63-237)	GT51_e8	GT51	N	3
contig_1_447	3.2.1.8	GH11(32-211)	GH11_e15	GH11	N	3
contig_1_456	N	GT119(17-362)	N	GT119	N	2
contig_1_529	-	GT2(4-133)	GT2	GT2	N	3
contig_1_534	3.2.1.-|3.2.1.17	GH73(734-861)	GH73_e162	GH73	N	3
contig_1_536	2.4.1.-	GT26(57-224)	GT26_e19	GT26	N	3
contig_1_538	-	GT2(39-168)	GT2	GT2	N	3
contig_1_550	-	GT4(215-345)	GT4_e23	GT4	N	3
contig_1_554	-	GT2(9-130)	GT2	GT2	N	3
contig_1_57	3.2.1.65|3.2.1.64	GH32(45-350)	GH32_e117	GH32	N	3
contig_1_58	2.4.1.10	GH68_1(42-469)	GH68_e0	GH68_1	N	3
contig_1_585	-	PL9_2(31-390)	PL9_e18	PL9_2	N	3
contig_1_605	3.5.1.25	CE9(8-384)	CE9_e51	CE9	N	3
contig_1_643	3.2.1.26	GH32(27-331)	GH32_e0	GH32	N	3
contig_1_662	-	GT4(193-345)	GT4_e2316	GT4	N	3
contig_1_663	-	GT2(10-139)	GT2	GT2	N	3
contig_1_664	-	GT4(197-305)	GT4_e1131	GT4	N	3
contig_1_665	N	GT122(39-352)	N	GT122	N	2
contig_1_666	-	GT2(7-151)	GT2	GT2	N	3
contig_1_668	-	GT2(7-132)	GT2	GT2	N	3
contig_1_69	-	GT4(253-405)	GT4_e3738	GT4	N	3
contig_1_710	-	CE14(4-114)	CE14_e46	N	N	2
contig_1_72	-	GT4(251-402)	GT4_e2274	GT4	N	3
contig_1_73	-	GT83(8-225)	GT83_e60	N	N	2
contig_1_754	N	GH109(1-152)	N	N	N	1
contig_1_850	3.2.1.132	GH46(38-262)	GH46_e0	GH46	N	3
contig_1_914	N	CE1(31-261)	N	N	N	1
contig_1_964	-	GT51(64-238)	GT51_e55	GT51	N	3
contig_1_988	5.4.99.11|3.2.1.20|3.2.1.10|3.2.1.-|3.2.1.70	GH13_31(28-371)	GH13_e122	GH13_31	N	3
contig_1_998	N	GH188(2-145)	N	N	N	1
contig_2_1008	2.4.1.-	GT1(179-387)	GT1_e225	GT1	N	3
contig_2_1020	3.2.1.85	GH1(3-463)	GH1_e65	GH1	N	3
contig_2_1026	-	GH53(40-338)	GH53_e0	GH53	N	3
contig_2_1043	N	CE1(15-237)	N	N	N	1
contig_2_1075	3.2.1.17|4.2.2.n1	GH23(123-226)	GH23_e335	GH23	N	3
contig_2_1205	2.4.1.129	GT51(55-229)	GT51_e20	GT51	N	3
contig_2_1251	-	CE4(81-207)	CE4_e25	CE4	N	3
contig_2_1310	-	AA6(2-169)	AA6_e2	N	N	2
contig_2_1339	1.1.3.-	AA7(21-242)	AA7_e0	N	N	2
contig_2_141	-	CE14(8-113)	CE14_e0	CE14	N	3
contig_2_142	2.4.1.-	GT4(196-344)	GT4_e1442	GT4	N	3
contig_2_156	2.4.1.129	GT51(87-263)	GT51_e113	GT51	N	3
contig_2_222	-	GH73(13-149)	GH73_e27	GH73	N	3
contig_2_231	-	GT4(318-470)	GT4_e3580	GT4	N	3
contig_2_254	2.4.1.157	GT28(204-354)	GT28_e89	GT28	N	3
contig_2_282	-	GT2(6-168)	GT2	GT2	N	3
contig_2_306	3.2.1.86|3.2.1.23|3.2.1.21	GH1(3-476)	GH1_e0	GH1	N	3
contig_2_307	-	GH30_3(82-488)	GH30_e56	GH30_3	N	3
contig_2_321	-	CE14(6-124)	CE14_e39	N	N	2
contig_2_326	-	GT1(9-393)	GT1_e505	GT1	N	3
contig_2_425	3.2.1.55|3.2.1.8|3.2.1.-|3.2.1.37	GH43_16(38-360)+	GH43_e261+CBM6_e6	CBM6+GH43_1	N	3
contig_2_426	3.2.1.8|3.2.1.136	GH30_8(33-419)	GH30_e78	GH30_8	N	3
contig_2_430	3.2.1.4|3.2.1.78|3.2.1.132	GH5_2(53-290)+C	GH5_e251+CBM3_e18	CBM3+GH5_2	N	3
contig_2_484	1.14.99.53	AA10(28-203)	AA10_e1	AA10	N	3
contig_2_504	3.2.1.37|3.2.1.55|3.2.1.-|-	GH43_11(3-301)+C	GH43_e285+CBM91_e24	CBM91+GH43_	N	3
contig_2_584	-	CE4(123-247)	CE4_e20	CE4	N	3
contig_2_729	-	GT28(188-350)	GT28_e1	GT28	N	3
contig_2_730	N	GT119(2-337)	N	GT119	N	2
contig_2_766	N	GT119(18-374)	N	GT119	N	2
contig_2_785	-	CE4(70-192)	CE4_e129	N	N	2
contig_2_923	-	GH18(171-401)	CBM50_e96+CBM50_e96+GH18_e174	CBM50+GH18	N	3
contig_2_933	-	GT2(11-95)	GT2	GT2	N	3
contig_2_962	-	GT2(8-100)	GT2	N	N	2
contig_2_963	-	GT83(10-262)	GT83_e35	N	N	2
contig_2_970	-	CBM50(161-203)	CBM50_e826	CBM50	N	3
contig_4_199	N	CE12(30-200)	N	N	N	1
contig_4_200	-	CE4(42-163)	CE4_e317	CE4	N	3
contig_4_207	-	CE6(55-146)	CE6_e1	CE0	N	3
contig_4_300	-	CBM50(4-48)	CBM50_e257	CBM50	N	3
contig_4_366	3.2.1.55	GH51_1(7-490)	GH51_e19	GH51_1	N	3
contig_4_384	-	AA4(11-235)	AA4_e1	N	N	2
contig_4_387	3.2.1.55	GH51_1(7-496)	GH51_e0	GH51_1	N	3
contig_4_396	3.2.1.99	GH43_5(41-319)	GH43_e56	GH43_5	N	3
contig_4_531	3.2.1.22	GH4(3-180)	GH4_e17	GH4	N	3
contig_5_104	5.4.99.11|3.2.1.20|3.2.1.10|3.2.1.-|3.2.1.70	GH13_31(28-375)	GH13_e122	GH13_31	N	3
contig_5_12	3.2.1.92	GH171(57-414)	GH171_e0	GH171	N	3
contig_5_122	3.2.1.1	GH13_28(58-339)+	GH13_e34+CBM26_e0	CBM26+GH13_	N	3
contig_5_13	3.2.1.52	GH3(108-343)	GH3_e28	GH3	N	3
contig_5_136	3.1.1.41|3.1.1.72	CE7(4-312)	CE7_e11	CE7	N	3
contig_5_238	-	GH126(42-350)	GH126_e0	GH126	N	3
contig_5_241	-	GT2(49-279)	GT2	GT2	N	3
contig_5_326	1.1.3.-	AA7(22-243)	AA7_e0	N	N	2
contig_5_365	-	GT1(95-391)	GT1_e274	GT1	N	3
contig_5_7	N	CE1(14-241)	N	N	N	1
contig_6_1	-	CE4(52-178)	CE4_e14	CE4	N	3
contig_7_38	N	AA1(48-499)	N	N	N	1
contig_8_102	3.5.1.104	CE4(59-185)	CE4_e23	CE4	N	3
contig_8_121	3.2.1.122	GH4(8-185)	GH4_e4	GH4	N	3
contig_8_154	-	GT2(7-168)	GT2	GT2	N	3
contig_8_30	-	GT2(4-129)	GT2	GT2	N	3
contig_8_37	-	GT2(5-127)	GT2	GT2	N	3
contig_8_48	4.2.2.2	PL1_6(135-348)	PL1_e108	PL1_6	N	3
contig_8_80	3.2.1.93	GH13_29(30-376)	GH13_e1	GH13_29	N	3
contig_2_220	-	N	GH23_e126	GH0	N	2
contig_2_327	-	N	CBM50_e817+CBM50_e1004+CBM50_e1	CBM50	N	2
contig_2_351	-	N	CBM50_e375+CBM50_e520	CBM50	N	2
contig_2_87	-	N	CBM50_e1023	N	N	1
contig_1_555	-	N	GT4_e101	GT4	N	2
contig_2_1272	-	N	CBM50_e520+CBM50_e438	CBM50	N	2
contig_2_1277	-	N	CBM50_e833+CBM50_e1004+CBM50_e4	CBM50	N	2
contig_2_357	-	N	GH23_e590	GH23	N	2
contig_2_850	-	N	CBM50_e55	N	N	1
contig_2_990	-	N	CBM50_e826	CBM50	N	2
contig_4_328	-	N	CBM50_e1	CBM50	N	2
contig_1_33	N	N	N	GH9	N	1
contig_1_60	N	N	N	CBM50	N	1
contig_1_94	N	N	N	GT22	N	1
contig_1_119	N	N	N	GH13_3	N	1
contig_1_182	N	N	N	CBM0+CBM16	N	1
contig_1_286	N	N	N	GH0	N	1
contig_1_308	N	N	N	GT0	N	1
contig_1_406	N	N	N	GH23	N	1
contig_1_417	N	N	N	GH13_11	N	1
contig_1_426	N	N	N	CBM48+GH13_	N	1
contig_1_482	N	N	N	GT2	N	1
contig_1_539	N	N	N	GT2	N	1
contig_1_543	N	N	N	GT0	N	1
contig_1_551	N	N	N	GT2	N	1
contig_1_669	N	N	N	GT2	N	1
contig_1_702	N	N	N	GH13_31	N	1
contig_1_757	N	N	N	CBM50	N	1
contig_1_786	N	N	N	CE8	N	1
contig_1_865	N	N	N	CE4	N	1
contig_1_876	N	N	N	GH0	N	1
contig_1_919	N	N	N	GT1	N	1
contig_2_1	N	N	N	GT0	N	1
contig_2_4	N	N	N	GT2	N	1
contig_2_127	N	N	N	GH1	N	1
contig_2_136	N	N	N	CE8	N	1
contig_2_148	N	N	N	GH24	N	1
contig_2_223	N	N	N	GH0	N	1
contig_2_331	N	N	N	GT2	N	1
contig_2_338	N	N	N	GH92	N	1
contig_2_394	N	N	N	GT1	N	1
contig_2_395	N	N	N	GT1	N	1
contig_2_396	N	N	N	GT1	N	1
contig_2_397	N	N	N	GT1	N	1
contig_2_398	N	N	N	GT2	N	1
contig_2_422	N	N	N	GT2	N	1
contig_2_423	N	N	N	GT1	N	1
contig_2_424	N	N	N	GT2	N	1
contig_2_480	N	N	N	GH0	N	1
contig_2_536	N	N	N	GT2	N	1
contig_2_537	N	N	N	GT2	N	1
contig_2_538	N	N	N	GT2	N	1
contig_2_539	N	N	N	GT2	N	1
contig_2_573	N	N	N	GT2	N	1
contig_2_585	N	N	N	GH13_48	N	1
contig_2_591	N	N	N	GT2	N	1
contig_2_597	N	N	N	CBM50+GH25	N	1
contig_2_645	N	N	N	GT2	N	1
contig_2_688	N	N	N	CBM50	N	1
contig_2_711	N	N	N	GT0	N	1
contig_2_793	N	N	N	GT2	N	1
contig_2_795	N	N	N	GT2	N	1
contig_2_796	N	N	N	GT2	N	1
contig_2_798	N	N	N	GT2	N	1
contig_2_886	N	N	N	GT4	N	1
contig_2_961	N	N	N	GT2	N	1
contig_2_1079	N	N	N	CBM50	N	1
contig_2_1283	N	N	N	GH130_1	N	1
contig_2_1295	N	N	N	CBM50	N	1
contig_2_1300	N	N	N	GH1	N	1
contig_2_1332	N	N	N	GH38	N	1
contig_3_1	N	N	N	GT2	N	1
contig_3_4	N	N	N	GT0	N	1
contig_4_15	N	N	N	GT30	N	1
contig_4_20	N	N	N	GH101	N	1
contig_4_176	N	N	N	GT1	N	1
contig_4_180	N	N	N	GH28	N	1
contig_4_234	N	N	N	GT4	N	1
contig_4_325	N	N	N	GH5_11	N	1
contig_4_337	N	N	N	GT2	N	1
contig_4_343	N	N	N	GT2	N	1
contig_4_408	N	N	N	GH28	N	1
contig_4_441	N	N	N	GH13_30	N	1
contig_4_501	N	N	N	GT2	N	1
contig_4_508	N	N	N	GT4	N	1
contig_4_530	N	N	N	GH4	N	1
contig_4_534	N	N	N	GT4	N	1
contig_4_559	N	N	N	GH13_3	N	1
contig_5_60	N	N	N	GH0	N	1
contig_5_87	N	N	N	CBM6	N	1
contig_5_158	N	N	N	GT1	N	1
contig_5_159	N	N	N	GT1	N	1
contig_5_160	N	N	N	GT2	N	1
contig_5_204	N	N	N	GH36	N	1
contig_5_239	N	N	N	GH126	N	1
contig_6_47	N	N	N	CE4	N	1
contig_6_51	N	N	N	GH1	N	1
contig_6_72	N	N	N	GT4	N	1
contig_7_14	N	N	N	GH13	N	1
contig_7_43	N	N	N	GH131	N	1
contig_7_66	N	N	N	GH13_23	N	1
contig_7_95	N	N	N	GT2	N	1
contig_7_99	N	N	N	GT0	N	1
contig_8_25	N	N	N	GT4	N	1
contig_8_29	N	N	N	GT0	N	1
contig_8_31	N	N	N	GT0	N	1
contig_8_85	N	N	N	CBM50	N	1
contig_8_123	N	N	N	GH4	N	1
contig_8_124	N	N	N	GH0	N	1
contig_12_15	N	N	N	GH84	N	1
contig_12_46	N	N	N	GT50	N	1
contig_12_59	N	N	N	GT119	N	1
contig_13_14	N	N	N	CBM13	N	1
contig_17_1	N	N	N	GT2	N	1
contig_17_2	N	N	N	GT2	N	1

S77.1_overview

Gene ID	EC#	HMMER	dbCAN_sub	DIAMOND	Signalp	#ofTools
contig_10_57	3.2.1.17	GH23(76-203)	GH23_e566	GH23	N	3
contig_12_12	3.2.1.92	GH171(57-414)	GH171_e0	GH171	N	3
contig_12_13	3.2.1.52	GH3(108-343)	GH3_e28	GH3	N	3
contig_12_7	N	CE1(14-241)	N	N	N	1
contig_13_27	5.4.99.11|3.2.1.20|3.2.1.10|3.2.1.-|3.2.1.70	GH13_31(28-375)	GH13_e122	GH13_31	N	3
contig_14_14	3.2.1.65|3.2.1.64	GH32(45-349)	GH32_e117	GH32	N	3
contig_14_15	2.4.1.10	GH68_1(42-469)	GH68_e0	GH68_1	N	3
contig_14_26	-	GT4(253-406)	GT4_e3738	GT4	N	3
contig_14_29	-	GT4(245-396)	GT4_e2274	GT4	N	3
contig_15_182	3.2.1.99	GH43_5(41-319)	GH43_e56	GH43_5	N	3
contig_15_57	3.2.1.22	GH4(284-461)	GH4_e17	GH4	N	3
contig_17_22	3.1.1.41|3.1.1.72	CE7(4-312)	CE7_e11	CE7	N	3
contig_17_7	3.2.1.86|3.2.1.38|3.2.1.85|3.2.1.21|3.2.1.-	GH1(9-475)	GH1_e115	GH1	N	3
contig_18_8	3.2.1.-	GH18(126-404)	CBM50_e699+GH18_e157	CBM50+GH18	N	3
contig_1_102	N	CE1(31-261)	N	N	N	1
contig_1_11	3.2.1.-	GH73(58-193)	GH73_e25	GH73	N	3
contig_1_169	3.2.1.132	GH46(38-261)	GH46_e0	GH46	N	3
contig_1_21	N	GH179(19-198)	N	N	N	1
contig_1_268	N	GH109(1-152)	N	N	N	1
contig_1_30	5.4.99.11|3.2.1.20|3.2.1.10|3.2.1.-|3.2.1.70	GH13_31(28-371)	GH13_e122	GH13_31	N	3
contig_1_312	-	CE14(4-114)	CE14_e46	N	N	2
contig_1_352	-	GT2(7-132)	GT2	GT2	N	3
contig_1_354	-	GT2(7-153)	GT2	GT2	N	3
contig_1_355	N	GT122(39-352)	N	GT122	N	2
contig_1_356	-	GT4(202-305)	GT4_e1131	GT4	N	3
contig_1_357	-	GT2(10-139)	GT2	GT2	N	3
contig_1_358	-	GT4(191-345)	GT4_e2316	GT4	N	3
contig_1_378	3.2.1.26	GH32(27-331)	GH32_e0	GH32	N	3
contig_1_417	3.5.1.25	CE9(8-384)	CE9_e51	CE9	N	3
contig_1_438	-	PL9_2(31-390)	PL9_e18	PL9_2	N	3
contig_1_469	-	GT2(9-123)	GT2	GT2	N	3
contig_1_473	-	GT4(216-345)	GT4_e23	GT4	N	3
contig_1_485	-	GT4(342-492)	GT4_e2213	GT4	N	3
contig_1_487	2.4.1.-	GT26(57-224)	GT26_e19	GT26	N	3
contig_1_489	3.2.1.-|3.2.1.17	GH73(734-861)	GH73_e162	GH73	N	3
contig_1_494	-	GT2(4-133)	GT2	GT2	N	3
contig_1_53	-	GT51(64-238)	GT51_e55	GT51	N	3
contig_1_565	N	GT119(17-362)	N	GT119	N	2
contig_1_652	-	GT51(63-237)	GT51_e8	GT51	N	3
contig_1_702	-	GT2(5-167)	GT2	GT2	N	3
contig_1_708	-	GT2(4-162)	GT2	GT2	N	3
contig_1_712	3.2.1.26	GH32(33-338)	GH32_e57	GH32	N	3
contig_1_723	N	GT119(19-384)	N	GT119	N	2
contig_1_737	-	GT2(8-218)	GT2	N	N	2
contig_1_754	-	GT8(5-257)	GT8_e207	GT8	N	3
contig_1_764	3.2.1.86	GH4(6-185)	GH4_e24	GH4	N	3
contig_1_779	3.2.1.86|3.2.1.85|3.2.1.21	GH1(7-465)	GH1_e13	GH1	N	3
contig_1_782	3.2.1.78|3.2.1.-	GH26(30-345)	GH26_e0	GH26	N	3
contig_1_792	-	CE4(115-242)	CE4_e274	N	N	2
contig_1_811	3.2.1.73|3.2.1.6|3.2.1.8	GH16_21(34-234)	GH16_e14	GH16_21	N	3
contig_1_823	-	PL1_8(88-284)	PL1_e106	PL1_8	N	3
contig_1_826	3.2.1.86|3.2.1.21|3.2.1.37	GH1(2-462)	GH1_e85	GH1	N	3
contig_1_834	3.2.1.99	GH43_4(33-359)	GH43_e38	GH43_4	N	3
contig_1_861	N	GH188(3-159)	N	N	N	1
contig_2_11	N	CE1(15-237)	N	N	N	1
contig_2_110	-	GH177(5-305)	GH109_e7	N	N	2
contig_2_175	2.4.1.129	GT51(55-229)	GT51_e20	GT51	N	3
contig_2_220	-	CE4(80-207)	CE4_e25	CE4	N	3
contig_2_279	-	AA6(2-169)	AA6_e2	N	N	2
contig_2_309	1.1.3.-	AA7(20-243)	AA7_e0	N	N	2
contig_2_47	3.2.1.17|4.2.2.n1	GH23(123-226)	GH23_e335	GH23	N	3
contig_3_128	2.4.1.157	GT28(204-354)	GT28_e89	GT28	N	3
contig_3_170	2.4.1.129	GT51(87-263)	GT51_e113	GT51	N	3
contig_3_184	2.4.1.-	GT4(196-344)	GT4_e1442	GT4	N	3
contig_3_185	-	CE14(8-113)	CE14_e0	CE14	N	3
contig_3_48	-	GT1(9-393)	GT1_e505	GT1	N	3
contig_3_511	N	CE12(25-195)	N	N	N	1
contig_3_512	-	CE4(42-163)	CE4_e317	CE4	N	3
contig_3_53	-	CE14(6-124)	CE14_e39	N	N	2
contig_3_613	-	CE6(55-146)	CE6_e1	CE0	N	3
contig_3_66	-	GH30_3(82-488)	GH30_e56	GH30_3	N	3
contig_3_67	3.2.1.86|3.2.1.23|3.2.1.21	GH1(3-476)	GH1_e0	GH1	N	3
contig_3_699	-	CBM50(4-48)	CBM50_e257	CBM50	N	3
contig_3_765	3.2.1.55	GH51_1(7-490)	GH51_e19	GH51_1	N	3
contig_3_783	-	AA4(30-234)	AA4_e1	N	N	2
contig_3_786	3.2.1.55	GH51_1(26-350)	GH51_e0	GH51_1	N	3
contig_3_789	3.2.1.20	GH31_1(261-708)	GH31_e23	GH31_1	N	3
contig_3_790	3.2.1.11	GH66(54-600)	GH66_e8	GH66	N	3
contig_3_93	-	GT2(6-168)	GT2	GT2	N	3
contig_4_11	3.2.1.37|3.2.1.55|3.2.1.-|-	GH43_11(3-301)+C	GH43_e285+CBM91_e24	CBM91+GH43_	N	3
contig_4_14	3.2.1.8	GH11(32-211)	GH11_e15	GH11	N	3
contig_4_7	3.2.1.8|3.1.1.72	GH11(40-221)	GH11_e22	GH11	N	3
contig_5_48	3.2.1.55|3.2.1.8|3.2.1.-|3.2.1.37	GH43_16(37-360)+	GH43_e261+CBM6_e6	CBM6+GH43_1	N	3
contig_5_49	3.2.1.8|3.2.1.136	GH30_8(34-420)	GH30_e78	GH30_8	N	3
contig_5_97	1.14.99.53	AA10(28-203)	AA10_e1	AA10	N	3
contig_6_1	-	CE4(51-178)	CE4_e14	CE4	N	3
contig_7_126	-	GT2(11-95)	GT2	GT2	N	3
contig_7_136	-	GH18(171-401)	CBM50_e96+CBM50_e96+	CBM50+GH18	N	3
contig_7_16	-	GH53(40-341)	GH53_e0	GH53	N	3
contig_7_19	3.2.1.85	GH1(3-463)	GH1_e65	GH1	N	3
contig_7_266	-	CE4(74-195)	CE4_e129	N	N	2
contig_7_286	N	GT119(18-374)	N	GT119	N	2
contig_7_32	2.4.1.-	GT1(183-387)	GT1_e225	GT1	N	3
contig_7_322	N	GT119(2-337)	N	GT119	N	2
contig_7_323	-	GT28(188-350)	GT28_e1	GT28	N	3
contig_7_362	3.2.1.1|3.2.1.98|3.2.1.41	GH13_5(60-400)	GH13_e5	GH13_5	N	3
contig_7_467	-	CE4(123-247)	CE4_e20	CE4	N	3
contig_7_492	-	GH113(782-1068)	GH113_e12	GH113	N	3
contig_7_494	3.2.1.17|4.2.2.n1	GH23(1442-1554)	GH23_e335	GH23	N	3
contig_7_91	-	CBM50(161-203)	CBM50_e826	CBM50	N	3
contig_7_98	-	GT83(11-263)	GT83_e74	N	N	2
contig_7_99	-	GT2(8-170)	GT2	GT2	N	3
contig_8_103	N	AA1(48-499)	N	N	N	1
contig_8_182	-	GT2(4-127)	GT2	GT2	N	3
contig_8_189	-	GT2(5-134)	GT2	GT2	N	3
contig_8_199	4.2.2.2	PL1_6(135-348)	PL1_e108	PL1_6	N	3
contig_8_227	3.2.1.93	GH13_29(30-376)	GH13_e1	GH13_29	N	3
contig_8_249	3.5.1.104	CE4(59-185)	CE4_e23	CE4	N	3
contig_8_275	3.2.1.122	GH4(8-185)	GH4_e4	GH4	N	3
contig_8_308	-	GT2(7-168)	GT2	GT2	N	3
contig_9_136	-	GT2(49-279)	GT2	GT2	N	3
contig_9_139	-	GH126(42-350)	GH126_e0	GH126	N	3
contig_9_62	1.1.3.-	AA7(22-197)	AA7_e0	N	N	2
contig_3_542	-	N	CBM50_e932	CBM50	N	2
contig_3_727	-	N	CBM50_e1	CBM50	N	2
contig_7_52	-	N	CBM50_e826	CBM50	N	2
contig_7_77	-	N	GH23_e126	GH23	N	2
contig_7_78	-	N	CBM50_e932	CBM50	N	2
contig_1_468	-	N	GT4_e101	GT4	N	2
contig_2_241	-	N	CBM50_e1004+CBM50_e4	CBM50	N	2
contig_2_246	-	N	CBM50_e833+CBM50_e10	CBM50	N	2
contig_3_17	-	N	GH23_e590	GH23	N	2
contig_3_23	-	N	CBM50_e375+CBM50_e52	CBM50	N	2
contig_3_240	-	N	CBM50_e1023	N	N	1
contig_3_47	-	N	CBM50_e817+CBM50_e10	CBM50	N	2
contig_3_543	-	N	GH23_e126	GH23	N	2
contig_7_208	-	N	CBM50_e55	N	N	1
contig_8_80	-	N	CBM50_e826	CBM50	N	2
contig_9_18	-	N	GT1_e274	N	N	1
contig_1_97	N	N	N	GT1	N	1
contig_1_139	N	N	N	GH0	N	1
contig_1_149	N	N	N	CE4	N	1
contig_1_233	N	N	N	CE8	N	1
contig_1_265	N	N	N	CBM50	N	1
contig_1_320	N	N	N	GH13_31	N	1
contig_1_351	N	N	N	GT2	N	1
contig_1_472	N	N	N	GT2	N	1
contig_1_480	N	N	N	GT0	N	1
contig_1_484	N	N	N	GT2	N	1
contig_1_539	N	N	N	GT2	N	1
contig_1_594	N	N	N	CBM48+GH13_	N	1
contig_1_603	N	N	N	GH13_11	N	1
contig_1_614	N	N	N	GH23	N	1
contig_1_696	N	N	N	GT0	N	1
contig_1_716	N	N	N	GH0	N	1
contig_1_888	N	N	N	GH13_3	N	1
contig_2_51	N	N	N	CBM50	N	1
contig_2_251	N	N	N	GH130_1	N	1
contig_2_263	N	N	N	CBM50	N	1
contig_2_269	N	N	N	GH1	N	1
contig_2_302	N	N	N	GH38	N	1
contig_3_36	N	N	N	GH92	N	1
contig_3_43	N	N	N	GT2	N	1
contig_3_178	N	N	N	GH24	N	1
contig_3_190	N	N	N	CE8	N	1
contig_3_199	N	N	N	GH1	N	1
contig_3_325	N	N	N	GT30	N	1
contig_3_330	N	N	N	GH101	N	1
contig_3_488	N	N	N	GT1	N	1
contig_3_492	N	N	N	GH28	N	1
contig_3_530	N	N	N	CBM50	N	1
contig_3_633	N	N	N	GT4	N	1
contig_3_724	N	N	N	GH5_11	N	1
contig_3_736	N	N	N	GT2	N	1
contig_3_742	N	N	N	GT2	N	1
contig_4_45	N	N	N	GT2	N	1
contig_4_46	N	N	N	GT2	N	1
contig_4_47	N	N	N	GT2	N	1
contig_4_48	N	N	N	GT2	N	1
contig_4_81	N	N	N	GT2	N	1
contig_5_10	N	N	N	GH28	N	1
contig_5_21	N	N	N	GT1	N	1
contig_5_22	N	N	N	GT2	N	1
contig_5_26	N	N	N	GT32	N	1
contig_5_45	N	N	N	GT2	N	1
contig_5_46	N	N	N	GT1	N	1
contig_5_47	N	N	N	GT2	N	1
contig_5_93	N	N	N	GH0	N	1
contig_6_46	N	N	N	CE4	N	1
contig_6_50	N	N	N	GH1	N	1
contig_6_71	N	N	N	GT4	N	1
contig_7_174	N	N	N	GT4	N	1
contig_7_341	N	N	N	GT0	N	1
contig_7_364	N	N	N	CBM50	N	1
contig_7_406	N	N	N	GT2	N	1
contig_7_454	N	N	N	CBM50+GH25	N	1
contig_7_460	N	N	N	GT2	N	1
contig_7_466	N	N	N	GH13_48	N	1
contig_7_490	N	N	N	GH0	N	1
contig_8_14	N	N	N	GH13	N	1
contig_8_83	N	N	N	GH0	N	1
contig_8_109	N	N	N	GH131	N	1
contig_8_127	N	N	N	GH13_23	N	1
contig_8_176	N	N	N	GT4	N	1
contig_8_181	N	N	N	GT0	N	1
contig_8_183	N	N	N	GT0	N	1
contig_8_232	N	N	N	CBM50	N	1
contig_8_277	N	N	N	GH4	N	1
contig_8_278	N	N	N	GH0	N	1
contig_9_138	N	N	N	GH126	N	1
contig_9_173	N	N	N	GH36	N	1
contig_9_219	N	N	N	GT1	N	1
contig_13_45	N	N	N	CBM6	N	1
contig_14_17	N	N	N	CBM50	N	1
contig_14_49	N	N	N	GT22	N	1
contig_15_30	N	N	N	GH13_3	N	1
contig_15_54	N	N	N	GT4	N	1
contig_15_79	N	N	N	GT4	N	1
contig_15_86	N	N	N	GT2	N	1
contig_15_142	N	N	N	GH13_30	N	1
contig_15_170	N	N	N	GH28	N	1
contig_16_16	N	N	N	GH84	N	1
contig_16_47	N	N	N	GT50	N	1
contig_16_60	N	N	N	GT119	N	1
contig_17_1	N	N	N	GT1	N	1
contig_18_14	N	N	N	CBM13	N	1
contig_23_1	N	N	N	GT1	N	1

N45.1_overview

Gene ID	EC#	HMMER	dbCAN_sub	DIAMOND	Signalp	#ofTools
contig_10_49	3.2.1.55|3.2.1.8|3.2.1.-|3.2.1.37	GH43_16(37-360)+CBM6(381-510)	GH43_e261+CBM6_e6	CBM6+GH43_16	N	3
contig_10_50	3.2.1.8|3.2.1.136	GH30_8(34-420)	GH30_e78	GH30_8	N	3
contig_10_97	1.14.99.53	AA10(28-203)	AA10_e1	AA10	N	3
contig_11_158	1.1.3.-	AA7(22-197)	AA7_e0	N	N	2
contig_11_81	-	GH126(42-350)	GH126_e0	GH126	N	3
contig_11_84	-	GT2(49-279)	GT2	GT2	N	3
contig_12_10	3.1.1.41|3.1.1.72	CE7(4-312)	CE7_e11	CE7	N	3
contig_12_25	3.2.1.86|3.2.1.38|3.2.1.85|3.2.1.21|3.2.1.-	GH1(9-475)	GH1_e115	GH1	N	3
contig_13_57	3.2.1.17	GH23(76-203)	GH23_e566	GH23	N	3
contig_13_78	3.2.1.65|3.2.1.64	GH32(45-349)	GH32_e117	GH32	N	3
contig_13_79	2.4.1.10	GH68_1(42-469)	GH68_e0	GH68_1	N	3
contig_13_90	-	GT4(253-406)	GT4_e3738	GT4	N	3
contig_13_93	-	GT4(245-396)	GT4_e2274	GT4	N	3
contig_16_10	3.2.1.-	GH18(126-404)	CBM50_e699+GH18_e157	CBM50+GH18	N	3
contig_1_1	-	CE4(51-178)	CE4_e14	CE4	N	3
contig_22_113	3.2.1.52	GH3(108-343)	GH3_e28	GH3	N	3
contig_22_114	3.2.1.92	GH171(57-414)	GH171_e0	GH171	N	3
contig_22_119	N	CE1(14-241)	N	N	N	1
contig_22_27	5.4.99.11|3.2.1.20|3.2.1.10|3.2.1.-|3.2.1.70	GH13_31(28-375)	GH13_e122	GH13_31	N	3
contig_2_140	3.2.1.22	GH4(284-461)	GH4_e17	GH4	N	3
contig_2_15	3.2.1.99	GH43_5(41-319)	GH43_e56	GH43_5	N	3
contig_3_102	N	CE1(31-261)	N	N	N	1
contig_3_11	3.2.1.-	GH73(58-193)	GH73_e25	GH73	N	3
contig_3_169	3.2.1.132	GH46(38-261)	GH46_e0	GH46	N	3
contig_3_21	N	GH179(19-198)	N	N	N	1
contig_3_268	N	GH109(1-152)	N	N	N	1
contig_3_30	5.4.99.11|3.2.1.20|3.2.1.10|3.2.1.-|3.2.1.70	GH13_31(28-371)	GH13_e122	GH13_31	N	3
contig_3_312	-	CE14(4-114)	CE14_e46	N	N	2
contig_3_351	-	GT2(7-132)	GT2	GT2	N	3
contig_3_353	-	GT2(7-153)	GT2	GT2	N	3
contig_3_354	N	GT122(39-352)	N	GT122	N	2
contig_3_355	-	GT4(202-305)	GT4_e1131	GT4	N	3
contig_3_356	-	GT2(10-139)	GT2	GT2	N	3
contig_3_357	-	GT4(191-345)	GT4_e2316	GT4	N	3
contig_3_377	3.2.1.26	GH32(27-331)	GH32_e0	GH32	N	3
contig_3_416	3.5.1.25	CE9(8-384)	CE9_e51	CE9	N	3
contig_3_437	-	PL9_2(31-390)	PL9_e18	PL9_2	N	3
contig_3_468	-	GT2(9-123)	GT2	GT2	N	3
contig_3_472	-	GT4(216-345)	GT4_e23	GT4	N	3
contig_3_484	-	GT4(342-492)	GT4_e2213	GT4	N	3
contig_3_486	2.4.1.-	GT26(57-224)	GT26_e19	GT26	N	3
contig_3_488	3.2.1.-|3.2.1.17	GH73(734-861)	GH73_e162	GH73	N	3
contig_3_493	-	GT2(4-133)	GT2	GT2	N	3
contig_3_53	-	GT51(64-238)	GT51_e55	GT51	N	3
contig_3_564	N	GT119(17-362)	N	GT119	N	2
contig_3_651	-	GT51(63-237)	GT51_e8	GT51	N	3
contig_3_701	-	GT2(5-167)	GT2	GT2	N	3
contig_3_707	-	GT2(4-162)	GT2	GT2	N	3
contig_3_711	3.2.1.26	GH32(33-338)	GH32_e57	GH32	N	3
contig_3_722	N	GT119(19-384)	N	GT119	N	2
contig_3_736	-	GT2(8-218)	GT2	N	N	2
contig_3_753	-	GT8(5-257)	GT8_e207	GT8	N	3
contig_3_763	3.2.1.86	GH4(6-185)	GH4_e24	GH4	N	3
contig_3_777	3.2.1.86|3.2.1.85|3.2.1.21	GH1(7-465)	GH1_e13	GH1	N	3
contig_3_780	3.2.1.78|3.2.1.-	GH26(30-348)	GH26_e0	GH26	N	3
contig_3_790	-	CE4(115-242)	CE4_e274	N	N	2
contig_3_809	3.2.1.73|3.2.1.6|3.2.1.8	GH16_21(34-234)	GH16_e14	GH16_21	N	3
contig_3_821	-	PL1_8(88-284)	PL1_e106	PL1_8	N	3
contig_3_824	3.2.1.86|3.2.1.21|3.2.1.37	GH1(2-462)	GH1_e85	GH1	N	3
contig_3_832	3.2.1.99	GH43_4(33-359)	GH43_e38	GH43_4	N	3
contig_3_859	N	GH188(3-159)	N	N	N	1
contig_4_142	-	GT2(4-127)	GT2	GT2	N	3
contig_4_149	-	GT2(5-134)	GT2	GT2	N	3
contig_4_159	4.2.2.2	PL1_6(135-348)	PL1_e108	PL1_6	N	3
contig_4_194	3.2.1.93	GH13_29(30-376)	GH13_e1	GH13_29	N	3
contig_4_216	3.5.1.104	CE4(59-185)	CE4_e23	CE4	N	3
contig_4_242	3.2.1.122	GH4(8-185)	GH4_e4	GH4	N	3
contig_4_274	-	GT2(7-168)	GT2	GT2	N	3
contig_4_63	N	AA1(48-499)	N	N	N	1
contig_6_128	2.4.1.157	GT28(204-354)	GT28_e89	GT28	N	3
contig_6_170	2.4.1.129	GT51(87-263)	GT51_e113	GT51	N	3
contig_6_184	2.4.1.-	GT4(196-344)	GT4_e1442	GT4	N	3
contig_6_185	-	CE14(8-113)	CE14_e0	CE14	N	3
contig_6_48	-	GT1(9-393)	GT1_e505	GT1	N	3
contig_6_512	N	CE12(25-195)	N	N	N	1
contig_6_513	-	CE4(42-163)	CE4_e317	CE4	N	3
contig_6_53	-	CE14(6-124)	CE14_e39	N	N	2
contig_6_615	-	CE6(55-146)	CE6_e1	CE0	N	3
contig_6_66	-	GH30_3(82-488)	GH30_e56	GH30_3	N	3
contig_6_67	3.2.1.86|3.2.1.23|3.2.1.21	GH1(3-476)	GH1_e0	GH1	N	3
contig_6_701	-	CBM50(4-48)	CBM50_e257	CBM50	N	3
contig_6_767	3.2.1.55	GH51_1(7-490)	GH51_e19	GH51_1	N	3
contig_6_785	-	AA4(30-234)	AA4_e1	N	N	2
contig_6_788	3.2.1.55	GH51_1(26-350)	GH51_e0	GH51_1	N	3
contig_6_791	3.2.1.20	GH31_1(261-708)	GH31_e23	GH31_1	N	3
contig_6_792	3.2.1.11	GH66(54-600)	GH66_e8	GH66	N	3
contig_6_93	-	GT2(6-168)	GT2	GT2	N	3
contig_7_131	-	CE4(80-207)	CE4_e25	CE4	N	3
contig_7_175	2.4.1.129	GT51(55-229)	GT51_e20	GT51	N	3
contig_7_239	-	GH177(5-305)	GH109_e7	N	N	2
contig_7_302	3.2.1.17|4.2.2.n1	GH23(123-226)	GH23_e335	GH23	N	3
contig_7_338	N	CE1(15-237)	N	N	N	1
contig_7_41	1.1.3.-	AA7(20-243)	AA7_e0	N	N	2
contig_7_71	-	AA6(2-169)	AA6_e2	N	N	2
contig_8_190	3.2.1.1|3.2.1.98|3.2.1.41	GH13_5(60-400)	GH13_e5	GH13_5	N	3
contig_8_229	-	GT28(188-350)	GT28_e1	GT28	N	3
contig_8_230	N	GT119(2-337)	N	GT119	N	2
contig_8_266	N	GT119(18-374)	N	GT119	N	2
contig_8_286	-	CE4(74-195)	CE4_e129	N	N	2
contig_8_417	-	GH18(171-401)	CBM50_e96+CBM50_e96+(	CBM50+GH18	N	3
contig_8_427	-	GT2(11-95)	GT2	GT2	N	3
contig_8_454	-	GT2(8-170)	GT2	GT2	N	3
contig_8_455	-	GT83(11-263)	GT83_e74	N	N	2
contig_8_462	-	CBM50(161-203)	CBM50_e826	CBM50	N	3
contig_8_521	2.4.1.-	GT1(183-387)	GT1_e225	GT1	N	3
contig_8_534	3.2.1.85	GH1(3-463)	GH1_e65	GH1	N	3
contig_8_537	-	GH53(40-341)	GH53_e0	GH53	N	3
contig_8_58	3.2.1.17|4.2.2.n1	GH23(1442-1554)	GH23_e335	GH23	N	3
contig_8_60	-	GH113(782-1068)	GH113_e12	GH113	N	3
contig_8_85	-	CE4(123-247)	CE4_e20	CE4	N	3
contig_9_11	3.2.1.37|3.2.1.55|3.2.1.-|-	GH43_11(3-301)+CBM91(331-532)	GH43_e285+CBM91_e24	CBM91+GH43_11	N	3
contig_9_14	3.2.1.8	GH11(32-211)	GH11_e15	GH11	N	3
contig_9_7	3.2.1.8|3.1.1.72	GH11(40-221)	GH11_e22	GH11	N	3
contig_11_202	-	N	GT1_e274	N	N	1
contig_3_467	-	N	GT4_e101	GT4	N	2
contig_6_17	-	N	GH23_e590	GH23	N	2
contig_6_23	-	N	CBM50_e375+CBM50_e520	CBM50	N	2
contig_6_47	-	N	CBM50_e817+CBM50_e100	CBM50	N	2
contig_6_543	-	N	CBM50_e932	CBM50	N	2
contig_8_476	-	N	GH23_e126	GH23	N	2
contig_4_40	-	N	CBM50_e826	CBM50	N	2
contig_6_240	-	N	CBM50_e1023	N	N	1
contig_6_544	-	N	GH23_e126	GH23	N	2
contig_6_729	-	N	CBM50_e1	CBM50	N	2
contig_7_105	-	N	CBM50_e833+CBM50_e100	CBM50	N	2
contig_7_110	-	N	CBM50_e1004+CBM50_e43	CBM50	N	2
contig_7_26	-	N	GH23_e126	GH23	N	2
contig_8_344	-	N	CBM50_e55	N	N	1
contig_8_475	-	N	CBM50_e932	CBM50	N	2
contig_8_501	-	N	CBM50_e826	CBM50	N	2
contig_1_46	N	N	N	CE4	N	1
contig_1_50	N	N	N	GH1	N	1
contig_1_71	N	N	N	GT4	N	1
contig_2_27	N	N	N	GH28	N	1
contig_2_55	N	N	N	GH13_30	N	1
contig_2_111	N	N	N	GT2	N	1
contig_2_118	N	N	N	GT4	N	1
contig_2_143	N	N	N	GT4	N	1
contig_2_167	N	N	N	GH13_3	N	1
contig_3_97	N	N	N	GT1	N	1
contig_3_139	N	N	N	GH0	N	1
contig_3_149	N	N	N	CE4	N	1
contig_3_233	N	N	N	CE8	N	1
contig_3_265	N	N	N	CBM50	N	1
contig_3_320	N	N	N	GH13_31	N	1
contig_3_350	N	N	N	GT2	N	1
contig_3_471	N	N	N	GT2	N	1
contig_3_479	N	N	N	GT0	N	1
contig_3_483	N	N	N	GT2	N	1
contig_3_538	N	N	N	GT2	N	1
contig_3_593	N	N	N	CBM48+GH13_8	N	1
contig_3_602	N	N	N	GH13_11	N	1
contig_3_613	N	N	N	GH23	N	1
contig_3_695	N	N	N	GT0	N	1
contig_3_715	N	N	N	GH0	N	1
contig_3_886	N	N	N	GH13_3	N	1
contig_4_43	N	N	N	GH0	N	1
contig_4_69	N	N	N	GH131	N	1
contig_4_87	N	N	N	GH13_23	N	1
contig_4_136	N	N	N	GT4	N	1
contig_4_141	N	N	N	GT0	N	1
contig_4_143	N	N	N	GT0	N	1
contig_4_199	N	N	N	CBM50	N	1
contig_4_244	N	N	N	GH4	N	1
contig_4_245	N	N	N	GH0	N	1
contig_5_1	N	N	N	GT119	N	1
contig_5_14	N	N	N	GT50	N	1
contig_5_45	N	N	N	GH84	N	1
contig_6_36	N	N	N	GH92	N	1
contig_6_43	N	N	N	GT2	N	1
contig_6_178	N	N	N	GH24	N	1
contig_6_190	N	N	N	CE8	N	1
contig_6_199	N	N	N	GH1	N	1
contig_6_326	N	N	N	GT30	N	1
contig_6_331	N	N	N	GH101	N	1
contig_6_489	N	N	N	GT1	N	1
contig_6_493	N	N	N	GH28	N	1
contig_6_531	N	N	N	CBM50	N	1
contig_6_635	N	N	N	GT4	N	1
contig_6_726	N	N	N	GH5_11	N	1
contig_6_738	N	N	N	GT2	N	1
contig_6_744	N	N	N	GT2	N	1
contig_7_29	N	N	N	GH0	N	1
contig_7_48	N	N	N	GH38	N	1
contig_7_81	N	N	N	GH1	N	1
contig_7_87	N	N	N	CBM50	N	1
contig_7_99	N	N	N	GH130_1	N	1
contig_7_298	N	N	N	CBM50	N	1
contig_8_62	N	N	N	GH0	N	1
contig_8_86	N	N	N	GH13_48	N	1
contig_8_92	N	N	N	GT2	N	1
contig_8_98	N	N	N	CBM50+GH25	N	1
contig_8_146	N	N	N	GT2	N	1
contig_8_188	N	N	N	CBM50	N	1
contig_8_211	N	N	N	GT0	N	1
contig_8_379	N	N	N	GT4	N	1
contig_9_45	N	N	N	GT2	N	1
contig_9_46	N	N	N	GT2	N	1
contig_9_47	N	N	N	GT2	N	1
contig_9_48	N	N	N	GT2	N	1
contig_9_81	N	N	N	GT2	N	1
contig_10_11	N	N	N	GH28	N	1
contig_10_22	N	N	N	GT1	N	1
contig_10_23	N	N	N	GT2	N	1
contig_10_27	N	N	N	GT32	N	1
contig_10_46	N	N	N	GT2	N	1
contig_10_47	N	N	N	GT1	N	1
contig_10_48	N	N	N	GT2	N	1
contig_10_93	N	N	N	GH0	N	1
contig_11_2	N	N	N	GT1	N	1
contig_11_47	N	N	N	GH36	N	1
contig_11_82	N	N	N	GH126	N	1
contig_12_31	N	N	N	GT1	N	1
contig_13_81	N	N	N	CBM50	N	1
contig_13_113	N	N	N	GT22	N	1
contig_16_4	N	N	N	CBM13	N	1
contig_19_2	N	N	N	GT1	N	1
contig_22_45	N	N	N	CBM6	N	1
contig_23_23	N	N	N	GH13	N	1

ATCC8473_overview

Gene ID	EC#	HMMER	dbCAN_sub	DIAMOND	Signalp	#ofTools
**contig_10_15**	1.14.99.53	AA10(28-203)	AA10_e1	AA10	N	3
**contig_10_62**	3.2.1.8|3.2.1.136	GH30_8(34-420)	GH30_e78	GH30_8	N	3
**contig_10_63**	3.2.1.55|3.2.1.8|3.2.1.-|3.2.1.37	GH43_16(37-360)+CBM6(381-510)	GH43_e261+CBM6_e6	CBM6+GH43_16	N	3
**contig_11_136**	-	GT2(49-279)	GT2	GT2	N	3
**contig_11_139**	-	GH126(42-350)	GH126_e0	GH126	N	3
**contig_11_62**	1.1.3.-	AA7(22-197)	AA7_e0	N	N	2
**contig_131_1**	-	CBM50(28-69)	CBM50_e35	N	N	2
**contig_137_1**	-	GH73(29-150)	GH73_e150	N	N	2
**contig_14_10**	3.1.1.41|3.1.1.72	CE7(4-312)	CE7_e11	CE7	N	3
**contig_14_25**	3.2.1.86|3.2.1.38|3.2.1.85|3.2.1.21|3.2.1.-	GH1(9-475)	GH1_e115	GH1	N	3
**contig_19_8**	3.2.1.-	GH18(126-404)	CBM50_e699+GH18_e157	CBM50+GH18	N	3
**contig_1_100**	-	GT2(8-170)	GT2	GT2	N	3
**contig_1_127**	-	GT2(11-95)	GT2	GT2	N	3
**contig_1_137**	-	GH18(171-401)	CBM50_e96+CBM50_e96+GH18_e174	CBM50+GH18	N	3
**contig_1_16**	-	GH53(40-341)	GH53_e0	GH53	N	3
**contig_1_19**	3.2.1.85	GH1(3-463)	GH1_e65	GH1	N	3
**contig_1_267**	-	CE4(74-195)	CE4_e129	N	N	2
**contig_1_287**	N	GT119(18-374)	N	GT119	N	2
**contig_1_32**	2.4.1.-	GT1(183-387)	GT1_e225	GT1	N	3
**contig_1_323**	N	GT119(2-337)	N	GT119	N	2
**contig_1_324**	-	GT28(188-350)	GT28_e1	GT28	N	3
**contig_1_364**	3.2.1.1|3.2.1.98|3.2.1.41	GH13_5(60-400)	GH13_e5	GH13_5	N	3
**contig_1_469**	-	CE4(123-247)	CE4_e20	CE4	N	3
**contig_1_494**	-	GH113(782-1068)	GH113_e12	GH113	N	3
**contig_1_496**	3.2.1.17|4.2.2.n1	GH23(1442-1554)	GH23_e335	GH23	N	3
**contig_1_92**	-	CBM50(161-203)	CBM50_e826	CBM50	N	3
**contig_1_99**	-	GT83(11-263)	GT83_e74	N	N	2
**contig_22_75**	-	CE4(51-178)	CE4_e14	CE4	N	3
**contig_2_128**	2.4.1.157	GT28(204-354)	GT28_e89	GT28	N	3
**contig_2_170**	2.4.1.129	GT51(87-263)	GT51_e113	GT51	N	3
**contig_2_184**	2.4.1.-	GT4(196-344)	GT4_e1442	GT4	N	3
**contig_2_185**	-	CE14(8-113)	CE14_e0	CE14	N	3
**contig_2_48**	-	GT1(9-393)	GT1_e505	GT1	N	3
**contig_2_512**	N	CE12(25-195)	N	N	N	1
**contig_2_513**	-	CE4(42-163)	CE4_e317	CE4	N	3
**contig_2_53**	-	CE14(6-124)	CE14_e39	N	N	2
**contig_2_616**	-	CE6(55-146)	CE6_e1	CE0	N	3
**contig_2_66**	-	GH30_3(82-488)	GH30_e56	GH30_3	N	3
**contig_2_67**	3.2.1.86|3.2.1.23|3.2.1.21	GH1(3-476)	GH1_e0	GH1	N	3
**contig_2_702**	-	CBM50(4-48)	CBM50_e257	CBM50	N	3
**contig_2_768**	3.2.1.55	GH51_1(7-490)	GH51_e19	GH51_1	N	3
**contig_2_786**	-	AA4(30-234)	AA4_e1	N	N	2
**contig_2_789**	3.2.1.55	GH51_1(26-350)	GH51_e0	GH51_1	N	3
**contig_2_792**	3.2.1.20	GH31_1(261-708)	GH31_e23	GH31_1	N	3
**contig_2_793**	3.2.1.11	GH66(54-600)	GH66_e8	GH66	N	3
**contig_2_93**	-	GT2(6-168)	GT2	GT2	N	3
**contig_3_102**	N	CE1(31-261)	N	N	N	1
**contig_3_11**	3.2.1.-	GH73(58-193)	GH73_e25	GH73	N	3
**contig_3_169**	3.2.1.132	GH46(38-261)	GH46_e0	GH46	N	3
**contig_3_21**	N	GH179(19-198)	N	N	N	1
**contig_3_268**	N	GH109(1-152)	N	N	N	1
**contig_3_30**	5.4.99.11|3.2.1.20|3.2.1.10|3.2.1.-|3.2.1.70	GH13_31(28-371)	GH13_e122	GH13_31	N	3
**contig_3_312**	-	CE14(4-114)	CE14_e46	N	N	2
**contig_3_351**	-	GT2(7-132)	GT2	GT2	N	3
**contig_3_353**	-	GT2(7-153)	GT2	GT2	N	3
**contig_3_354**	N	GT122(39-352)	N	GT122	N	2
**contig_3_355**	-	GT4(202-305)	GT4_e1131	GT4	N	3
**contig_3_356**	-	GT2(10-139)	GT2	GT2	N	3
**contig_3_357**	-	GT4(191-345)	GT4_e2316	GT4	N	3
**contig_3_377**	3.2.1.26	GH32(27-331)	GH32_e0	GH32	N	3
**contig_3_416**	3.5.1.25	CE9(8-384)	CE9_e51	CE9	N	3
**contig_3_437**	-	PL9_2(31-390)	PL9_e18	PL9_2	N	3
**contig_3_468**	-	GT2(9-123)	GT2	GT2	N	3
**contig_3_472**	-	GT4(216-345)	GT4_e23	GT4	N	3
**contig_3_484**	-	GT4(342-492)	GT4_e2213	GT4	N	3
**contig_3_486**	2.4.1.-	GT26(57-224)	GT26_e19	GT26	N	3
**contig_3_488**	3.2.1.-|3.2.1.17	GH73(734-861)	GH73_e162	GH73	N	3
**contig_3_493**	-	GT2(4-133)	GT2	GT2	N	3
**contig_3_53**	-	GT51(64-238)	GT51_e55	GT51	N	3
**contig_3_564**	N	GT119(17-362)	N	GT119	N	2
**contig_3_651**	-	GT51(63-237)	GT51_e8	GT51	N	3
**contig_3_701**	-	GT2(5-167)	GT2	GT2	N	3
**contig_3_707**	-	GT2(4-162)	GT2	GT2	N	3
**contig_3_711**	3.2.1.26	GH32(33-338)	GH32_e57	GH32	N	3
**contig_3_722**	N	GT119(19-384)	N	GT119	N	2
**contig_3_736**	-	GT2(8-218)	GT2	N	N	2
**contig_3_753**	-	GT8(5-257)	GT8_e207	GT8	N	3
**contig_3_763**	3.2.1.86	GH4(6-185)	GH4_e24	GH4	N	3
**contig_3_777**	3.2.1.86|3.2.1.85|3.2.1.21	GH1(7-465)	GH1_e13	GH1	N	3
**contig_3_780**	3.2.1.78|3.2.1.-	GH26(30-348)	GH26_e0	GH26	N	3
**contig_3_790**	-	CE4(115-242)	CE4_e274	N	N	2
**contig_3_809**	3.2.1.73|3.2.1.6|3.2.1.8	GH16_21(34-234)	GH16_e14	GH16_21	N	3
**contig_3_821**	-	PL1_8(88-284)	PL1_e106	PL1_8	N	3
**contig_3_824**	3.2.1.86|3.2.1.21|3.2.1.37	GH1(2-462)	GH1_e85	GH1	N	3
**contig_3_832**	3.2.1.99	GH43_4(33-359)	GH43_e38	GH43_4	N	3
**contig_3_859**	N	GH188(3-159)	N	N	N	1
**contig_4_43**	-	GT4(245-396)	GT4_e2274	GT4	N	3
**contig_4_46**	-	GT4(253-406)	GT4_e3738	GT4	N	3
**contig_4_57**	2.4.1.10	GH68_1(42-469)	GH68_e0	GH68_1	N	3
**contig_4_58**	3.2.1.65|3.2.1.64	GH32(45-349)	GH32_e117	GH32	N	3
**contig_4_79**	3.2.1.17	GH23(76-203)	GH23_e566	GH23	N	3
**contig_5_11**	N	CE1(15-237)	N	N	N	1
**contig_5_110**	-	GH177(5-305)	GH109_e7	N	N	2
**contig_5_174**	2.4.1.129	GT51(55-229)	GT51_e20	GT51	N	3
**contig_5_218**	-	CE4(80-207)	CE4_e25	CE4	N	3
**contig_5_278**	-	AA6(2-169)	AA6_e2	N	N	2
**contig_5_308**	1.1.3.-	AA7(20-243)	AA7_e0	N	N	2
**contig_5_47**	3.2.1.17|4.2.2.n1	GH23(123-226)	GH23_e335	GH23	N	3
**contig_6_183**	3.2.1.8	GH11(32-211)	GH11_e15	GH11	N	3
**contig_6_186**	3.2.1.37|3.2.1.55|3.2.1.-|-	GH43_11(3-301)+CBM91(331-532)	GH43_e285+CBM91_e24	CBM91+GH43_11	N	3
**contig_6_190**	3.2.1.8|3.1.1.72	GH11(40-221)	GH11_e22	GH11	N	3
**contig_7_12**	3.2.1.92	GH171(57-414)	GH171_e0	GH171	N	3
**contig_7_13**	3.2.1.52	GH3(108-343)	GH3_e28	GH3	N	3
**contig_7_7**	N	CE1(14-241)	N	N	N	1
**contig_7_99**	5.4.99.11|3.2.1.20|3.2.1.10|3.2.1.-|3.2.1.70	GH13_31(28-375)	GH13_e122	GH13_31	N	3
**contig_8_103**	N	AA1(48-499)	N	N	N	1
**contig_8_182**	-	GT2(4-127)	GT2	GT2	N	3
**contig_8_189**	-	GT2(5-134)	GT2	GT2	N	3
**contig_8_199**	4.2.2.2	PL1_6(135-348)	PL1_e108	PL1_6	N	3
**contig_8_231**	3.2.1.93	GH13_29(30-376)	GH13_e1	GH13_29	N	3
**contig_8_253**	3.5.1.104	CE4(59-185)	CE4_e23	CE4	N	3
**contig_8_279**	3.2.1.122	GH4(8-185)	GH4_e4	GH4	N	3
**contig_8_311**	-	GT2(7-168)	GT2	GT2	N	3
**contig_9_181**	3.2.1.99	GH43_5(41-319)	GH43_e56	GH43_5	N	3
**contig_9_57**	3.2.1.22	GH4(284-461)	GH4_e17	GH4	N	3
**contig_11_18**	-	N	GT1_e274	N	N	1
**contig_1_209**	-	N	CBM50_e55	N	N	1
**contig_1_52**	-	N	CBM50_e826	CBM50	N	2
**contig_2_17**	-	N	GH23_e590	GH23	N	2
**contig_2_23**	-	N	CBM50_e375+CBM50_e520	CBM50	N	2
**contig_2_47**	-	N	CBM50_e817+CBM50_e1004+CBM50_e	CBM50	N	2
**contig_2_544**	-	N	GH23_e126	GH23	N	2
**contig_3_467**	-	N	GT4_e101	GT4	N	2
**contig_5_323**	-	N	GH23_e126	GH23	N	2
**contig_1_78**	-	N	GH23_e126	GH23	N	2
**contig_1_79**	-	N	CBM50_e932	CBM50	N	2
**contig_2_240**	-	N	CBM50_e1023	N	N	1
**contig_2_543**	-	N	CBM50_e932	CBM50	N	2
**contig_2_730**	-	N	CBM50_e1	CBM50	N	2
**contig_5_239**	-	N	CBM50_e1004+CBM50_e438	CBM50	N	2
**contig_5_244**	-	N	CBM50_e833+CBM50_e1004+CBM50_e	CBM50	N	2
**contig_8_80**	-	N	CBM50_e826	CBM50	N	2
**contig_1_175**	N	N	N	GT4	N	1
**contig_1_342**	N	N	N	GT0	N	1
**contig_1_366**	N	N	N	CBM50	N	1
**contig_1_408**	N	N	N	GT2	N	1
**contig_1_456**	N	N	N	CBM50+GH25	N	1
**contig_1_462**	N	N	N	GT2	N	1
**contig_1_468**	N	N	N	GH13_48	N	1
**contig_1_492**	N	N	N	GH0	N	1
**contig_2_36**	N	N	N	GH92	N	1
**contig_2_43**	N	N	N	GT2	N	1
**contig_2_178**	N	N	N	GH24	N	1
**contig_2_190**	N	N	N	CE8	N	1
**contig_2_199**	N	N	N	GH1	N	1
**contig_2_326**	N	N	N	GT30	N	1
**contig_2_331**	N	N	N	GH101	N	1
**contig_2_489**	N	N	N	GT1	N	1
**contig_2_493**	N	N	N	GH28	N	1
**contig_2_531**	N	N	N	CBM50	N	1
**contig_2_636**	N	N	N	GT4	N	1
**contig_2_727**	N	N	N	GH5_11	N	1
**contig_2_739**	N	N	N	GT2	N	1
**contig_2_745**	N	N	N	GT2	N	1
**contig_3_97**	N	N	N	GT1	N	1
**contig_3_139**	N	N	N	GH0	N	1
**contig_3_149**	N	N	N	CE4	N	1
**contig_3_233**	N	N	N	CE8	N	1
**contig_3_265**	N	N	N	CBM50	N	1
**contig_3_320**	N	N	N	GH13_31	N	1
**contig_3_350**	N	N	N	GT2	N	1
**contig_3_471**	N	N	N	GT2	N	1
**contig_3_479**	N	N	N	GT0	N	1
**contig_3_483**	N	N	N	GT2	N	1
**contig_3_538**	N	N	N	GT2	N	1
**contig_3_593**	N	N	N	CBM48+GH13_8	N	1
**contig_3_602**	N	N	N	GH13_11	N	1
**contig_3_613**	N	N	N	GH23	N	1
**contig_3_695**	N	N	N	GT0	N	1
**contig_3_715**	N	N	N	GH0	N	1
**contig_3_886**	N	N	N	GH13_3	N	1
**contig_4_23**	N	N	N	GT22	N	1
**contig_4_55**	N	N	N	CBM50	N	1
**contig_5_51**	N	N	N	CBM50	N	1
**contig_5_250**	N	N	N	GH130_1	N	1
**contig_5_262**	N	N	N	CBM50	N	1
**contig_5_268**	N	N	N	GH1	N	1
**contig_5_301**	N	N	N	GH38	N	1
**contig_5_320**	N	N	N	GH0	N	1
**contig_6_115**	N	N	N	GT2	N	1
**contig_6_148**	N	N	N	GT2	N	1
**contig_6_149**	N	N	N	GT2	N	1
**contig_6_150**	N	N	N	GT2	N	1
**contig_6_151**	N	N	N	GT2	N	1
**contig_7_81**	N	N	N	CBM6	N	1
**contig_8_14**	N	N	N	GH13	N	1
**contig_8_83**	N	N	N	GH0	N	1
**contig_8_109**	N	N	N	GH131	N	1
**contig_8_127**	N	N	N	GH13_23	N	1
**contig_8_176**	N	N	N	GT4	N	1
**contig_8_181**	N	N	N	GT0	N	1
**contig_8_183**	N	N	N	GT0	N	1
**contig_8_236**	N	N	N	CBM50	N	1
**contig_8_281**	N	N	N	GH4	N	1
**contig_8_282**	N	N	N	GH0	N	1
**contig_9_30**	N	N	N	GH13_3	N	1
**contig_9_54**	N	N	N	GT4	N	1
**contig_9_79**	N	N	N	GT4	N	1
**contig_9_86**	N	N	N	GT2	N	1
**contig_9_141**	N	N	N	GH13_30	N	1
**contig_9_169**	N	N	N	GH28	N	1
**contig_10_19**	N	N	N	GH0	N	1
**contig_10_64**	N	N	N	GT2	N	1
**contig_10_65**	N	N	N	GT1	N	1
**contig_10_66**	N	N	N	GT2	N	1
**contig_10_85**	N	N	N	GT32	N	1
**contig_10_89**	N	N	N	GT2	N	1
**contig_10_90**	N	N	N	GT1	N	1
**contig_10_101**	N	N	N	GH28	N	1
**contig_11_138**	N	N	N	GH126	N	1
**contig_11_173**	N	N	N	GH36	N	1
**contig_11_218**	N	N	N	GT1	N	1
**contig_12_1**	N	N	N	GT119	N	1
**contig_12_14**	N	N	N	GT50	N	1
**contig_12_45**	N	N	N	GH84	N	1
**contig_14_31**	N	N	N	GT1	N	1
**contig_19_14**	N	N	N	CBM13	N	1
**contig_20_1**	N	N	N	GT1	N	1
**contig_22_4**	N	N	N	GT4	N	1
**contig_22_25**	N	N	N	GH1	N	1
**contig_22_29**	N	N	N	CE4	N	1

S110.5_overview

Gene ID	EC#	HMMER	dbCAN_sub	DIAMOND	Signalp	#ofTools
contig_11_1	-	CE4(50-179)	CE4_e14	CE4	N	3
contig_12_122	5.4.99.11|3.2.1.20|3.2.1.10|3.2.1.-|3.2.1.70	GH13_31(28-375)	GH13_e122	GH13_31	N	3
contig_12_146	3.2.1.1	GH13_28(58-339)+CBM26(564-642)	GH13_e34+CBM26_e0	CBM26+GH13_28	N	3
contig_12_161	3.1.1.41|3.1.1.72	CE7(3-312)	CE7_e11	CE7	N	3
contig_12_189	3.2.1.86|3.2.1.38|3.2.1.85|3.2.1.21|3.2.1.-	GH1(7-474)	GH1_e115	GH1	N	3
contig_12_3	N	CE1(2-228)	N	N	N	1
contig_12_8	3.2.1.92	GH171(57-414)	GH171_e0	GH171	N	3
contig_12_9	3.2.1.52	GH3(111-347)	GH3_e28	GH3	N	3
contig_14_141	N	GH177(3-295)	N	N	N	1
contig_14_184	N	GH188(5-209)	N	N	N	1
contig_14_194	3.2.1.99	GH43_4(37-363)	GH43_e38	GH43_4	N	3
contig_14_203	3.2.1.86|3.2.1.21|3.2.1.37	GH1(6-466)	GH1_e85	GH1	N	3
contig_14_61	-	GT83(9-225)	GT83_e5	N	N	2
contig_15_111	3.2.1.17|4.2.2.n1	GH23(138-241)	GH23_e335	GH23	N	3
contig_15_141	N	CE1(15-237)	N	N	N	1
contig_15_2	-	GH188(3-206)	GH109_e7	N	N	2
contig_15_34	N	GH177(5-304)	N	N	N	1
contig_16_83	-	GH126(45-353)	GH126_e0	GH126	N	3
contig_16_86	-	GT2(49-279)	GT2	GT2	N	3
contig_17_131	3.2.1.41	CBM68(5-97)+GH13_14(257-570)	CBM68_e0+CBM48_e46+GH13_e94	CBM48+CBM68+GH13_14	N	3
contig_17_147	N	GH188(30-212)	N	N	N	1
contig_17_149	3.2.1.172	GH105(30-362)	GH105_e52	GH105	N	3
contig_17_161	-	CE19(14-298)	CE19_e3	N	N	2
contig_17_166	3.2.1.22	GH4(3-181)	GH4_e17	GH4	N	3
contig_17_18	3.2.1.99	GH43_5(43-321)	GH43_e56	GH43_5	N	3
contig_17_2	-	AA4(8-239)	AA4_e1	N	N	2
contig_17_222	2.4.1.1	GT35(93-795)	GT35_e0	GT35	N	3
contig_17_223	2.4.1.21	GT5(1-452)	GT5_e9	GT5	N	3
contig_17_226	2.4.1.18	CBM48(26-109)+GH13_9(178-468)	CBM48_e2+GH13_e200	CBM48+GH13_9	N	3
contig_17_9	3.2.1.55	GH51_1(6-496)	GH51_e0	GH51_1	N	3
contig_18_16	-	AA6(2-169)	AA6_e2	N	N	2
contig_19_19	-	CE14(4-114)	CE14_e46	N	N	2
contig_19_56	N	GH179(33-197)	N	N	N	1
contig_1_9	3.2.1.-	GH18(126-405)	CBM50_e699+CBM50_e699+GH18_e157	CBM50+GH18	N	3
contig_20_2	N	GT119(19-372)	N	GT119	N	2
contig_21_29	-	GT2(9-114)	GT2	GT2	N	3
contig_21_33	-	GT4(218-351)	GT4_e23	GT4	N	3
contig_21_36	-	GT2(4-136)	GT2	GT2	N	3
contig_21_42	-	GT2(7-132)	GT2	GT2	N	3
contig_21_43	-	GT4(327-476)	GT4_e14	GT4	N	3
contig_21_49	-	GT2(7-153)	GT2	GT2	N	3
contig_21_51	-	GT2(2-112)	GT2	GT2	N	3
contig_21_54	2.4.1.-	GT26(57-224)	GT26_e19	GT26	N	3
contig_23_103	3.2.1.-	GH73(59-194)	GH73_e25	GH73	N	3
contig_23_12	N	CE1(36-267)	N	N	N	1
contig_23_62	-	GT51(63-239)	GT51_e55	GT51	N	3
contig_23_84	5.4.99.11|3.2.1.20|3.2.1.10|3.2.1.-|3.2.1.70	GH13_31(28-372)	GH13_e122	GH13_31	N	3
contig_23_98	N	GH188(2-145)	N	N	N	1
contig_24_27	-	CE4(80-207)	CE4_e25	CE4	N	3
contig_24_70	2.4.1.129	GT51(56-230)	GT51_e20	GT51	N	3
contig_26_12	3.1.1.-	CE12(179-374)	CE12_e21	CE12	N	3
contig_26_21	N	CBM16(1290-1426)	N	CBM0+CBM16	N	2
contig_26_5	3.2.1.21|3.2.1.-|3.2.1.23	GH1(8-468)	GH1_e87	GH1	N	3
contig_27_101	-	GT2(8-170)	GT2	GT2	N	3
contig_27_102	-	GT83(5-262)	GT83_e35	N	N	2
contig_27_110	-	CBM50(161-203)	CBM50_e826	CBM50	N	3
contig_27_123	-	CBM50(161-218)	CBM50_e932	CBM50	N	3
contig_27_175	-	GT1(203-386)	GT1_e509	GT1	N	3
contig_27_4	-	GH18(31-301)	GH18_e71	GH18	N	3
contig_27_47	-	GT2(9-179)	GT2	GT2	N	3
contig_27_51	-	GT28(198-342)	GT28_e9	GT28	N	3
contig_27_72	-	GT2(7-135)	GT2	GT2	N	3
contig_28_1	3.2.1.86	GH4(6-185)	GH4_e24	GH4	N	3
contig_28_25	-	CE4(113-242)	CE4_e274	N	N	2
contig_29_19	3.2.1.132	GH46(38-262)	GH46_e0	GH46	N	3
contig_29_34	3.5.1.104	CE4(272-383)	CE4_e217	CE4	N	3
contig_2_17	-	GT1(59-389)	GT1_e274	GT1	N	3
contig_2_18	-	GH18(171-401)	CBM50_e96+CBM50_e96+GH18_e174	CBM50+GH18	N	3
contig_2_2	3.2.1.78|3.2.1.-	GH26(32-350)	GH26_e0	GH26	N	3
contig_2_35	N	GH188(5-234)	N	N	N	1
contig_2_6	3.2.1.86|3.2.1.85|3.2.1.21	GH1(7-464)	GH1_e13	GH1	N	3
contig_30_9	3.2.1.-|3.2.1.17	GH73(735-862)	GH73_e162	GH73	N	3
contig_35_14	1.14.99.53	AA10(28-203)	AA10_e1	AA10	N	3
contig_35_18	3.2.1.37|3.2.1.55|3.2.1.-|-	GH43_11(3-301)+CBM91(331-532)	GH43_e285+CBM91_e24	CBM91+GH43_11	N	3
contig_35_32	4.2.2.10	PL1_8(83-276)	PL1_e25	PL1_8	N	3
contig_35_34	-	CBM63(139-214)	CBM63_e7	CBM63	N	3
contig_3_118	2.4.1.129	GT51(84-260)	GT51_e113	GT51	N	3
contig_3_132	2.4.1.-	GT4(196-344)	GT4_e1442	GT4	N	3
contig_3_133	-	CE14(7-112)	CE14_e0	CE14	N	3
contig_3_14	-	CE14(6-124)	CE14_e39	N	N	2
contig_3_323	3.2.1.8	GH11(32-211)	GH11_e15	GH11	N	3
contig_3_332	3.2.1.73|3.2.1.6|3.2.1.8	GH16_21(37-238)	GH16_e14	GH16_21	N	3
contig_3_351	3.2.1.26	GH32(27-330)	GH32_e0	GH32	N	3
contig_3_381	3.2.1.1|3.2.1.98|3.2.1.41	GH13_5(62-402)	GH13_e5	GH13_5	N	3
contig_3_447	-	CE4(123-245)	CE4_e20	CE4	N	3
contig_3_49	-	GT2(6-168)	GT2	GT2	N	3
contig_3_594	-	GT28(188-350)	GT28_e1	GT28	N	3
contig_3_595	N	GT119(2-337)	N	GT119	N	2
contig_3_78	2.4.1.157	GT28(205-353)	GT28_e89	GT28	N	3
contig_3_9	-	GT1(191-396)	GT1_e505	GT1	N	3
contig_4_131	N	AA1(48-499)	N	N	N	1
contig_4_27	-	GT2(4-126)	GT2	GT2	N	3
contig_4_3	4.2.2.2	PL1_6(135-347)	PL1_e108	PL1_6	N	3
contig_4_44	3.2.1.67	GH4(11-186)	GH4_e30	GH4	N	3
contig_4_48	4.2.2.24	PL26(3-845)	PL26_e0	PL26	N	3
contig_4_49	3.2.1.23	GH42(10-371)	GH42_e10	GH42	N	3
contig_4_50	3.1.1.-	CE12(5-201)	CE12_e0	CE12	N	3
contig_4_52	4.2.2.23|4.2.2.24	PL11(1-601)	PL11_e0	PL11_1	N	3
contig_4_53	4.2.2.23|4.2.2.24	PL11(33-613)	PL11_e0	PL11_1	N	3
contig_4_56	3.1.1.72	CE12(6-211)	CE12_e45	CE12	N	3
contig_4_58	3.2.1.172	GH105(25-337)	GH105_e0	GH105	N	3
contig_5_110	-	GT2(5-172)	GT2	GT2	N	3
contig_5_111	-	GT2(5-112)	GT2	GT2	N	3
contig_5_113	-	GT2(11-220)	GT2	N	N	2
contig_5_17	-	GT2(7-168)	GT2	GT2	N	3
contig_5_55	3.2.1.122	GH4(8-185)	GH4_e4	GH4	N	3
contig_5_75	3.5.1.104	CE4(59-185)	CE4_e23	CE4	N	3
contig_5_92	3.2.1.93	GH13_29(30-377)	GH13_e1	GH13_29	N	3
contig_6_137	-	GT4(177-325)	GT4_e992	GT4	N	3
contig_6_17	3.2.1.55|3.2.1.8|3.2.1.-|3.2.1.37	GH43_16(38-361)+CBM6(382-511)	GH43_e261+CBM6_e6	CBM6+GH43_16	N	3
contig_6_18	3.2.1.8|3.2.1.136	GH30_8(34-420)	GH30_e78	GH30_8	N	3
contig_6_19	-	CE6(55-146)	CE6_e1	CE0	N	3
contig_6_216	N	CE12(25-196)	N	N	N	1
contig_6_22	3.2.1.4|3.2.1.78|3.2.1.132	GH5_2(52-290)+CBM3(357-437)	GH5_e251+CBM3_e18	CBM3+GH5_2	N	3
contig_6_268	3.2.1.65|3.2.1.80|3.2.1.26|3.2.1.153|3.2.1.64	GH32(39-349)+CBM66(520-673)	GH32_e16+CBM66_e0	CBM66+GH32	N	3
contig_6_348	-	GH188(8-251)	GH109_e7	N	N	2
contig_6_355	-	CBM50(4-48)	CBM50_e257	CBM50	N	3
contig_6_424	3.2.1.55	GH51_1(7-490)	GH51_e19	GH51_1	N	3
contig_7_12	3.2.1.89	GH53(54-417)	GH53_e14	GH53	N	3
contig_7_13	3.2.1.23	GH42(20-399)	GH42_e20	GH42	N	3
contig_7_2	-	GH18(33-331)	GH18_e163	GH18	N	3
contig_7_27	-	GT2(6-147)	GT2	GT2	N	3
contig_7_29	-	GT2(7-169)	GT2	GT2	N	3
contig_7_30	N	GT122(39-352)	N	GT122	N	2
contig_7_31	-	GT4(204-310)	GT4_e1080	GT4	N	3
contig_7_32	-	GT2(8-160)	GT2	GT2	N	3
contig_7_33	-	GT4(190-345)	GT4_e2316	GT4	N	3
contig_7_48	2.4.1.10	GH68_1(41-469)	GH68_e0	GH68_1	N	3
contig_7_49	3.2.1.65|3.2.1.64	GH32(48-355)	GH32_e117	GH32	N	3
contig_7_55	1.1.3.-	AA7(23-242)	AA7_e0	N	N	2
contig_7_91	4.2.2.2	PL3_1(28-204)	PL3_e8	PL3_1	N	3
contig_7_97	3.5.1.25	CE9(9-384)	CE9_e51	CE9	N	3
contig_8_109	-	GT51(62-238)	GT51_e8	GT51	N	3
contig_8_13	-	GT8(5-258)	GT8_e207	GT8	N	3
contig_8_218	-	PL9_2(33-390)	PL9_e18	PL9_2	N	3
contig_8_45	N	GT119(19-382)	N	GT119	N	2
contig_8_53	3.2.1.26	GH32(33-338)	GH32_e57	GH32	N	3
contig_8_60	-	GT2(4-163)	GT2	GT2	N	3
contig_8_67	-	GT2(5-167)	GT2	GT2	N	3
contig_22_19	-	N	CBM50_e55	N	N	1
contig_24_2	-	N	CBM50_e1004+CBM50_e1004+CBM50_e4	CBM50	N	2
contig_27_149	-	N	CBM50_e826	CBM50	N	2
contig_27_6	-	N	CBM50_e1022	CBM50	N	2
contig_2_100	3.2.1.17	N	GH23_e566	N	N	1
contig_37_10	-	N	GH23_e590	GH23	N	2
contig_13_18	-	N	CBM50_e833+CBM50_e1004+CBM50_e10	CBM50	N	2
contig_21_28	-	N	GT4_e101	GT4	N	2
contig_27_124	-	N	GH23_e126	GH0	N	2
contig_37_20	3.2.1.96|3.2.1.-|3.2.1.17|-	N	CBM50_e375+CBM50_e259	CBM50	N	2
contig_3_188	-	N	CBM50_e1023	N	N	1
contig_3_8	-	N	CBM50_e1004+CBM50_e438+CBM50_e43	CBM50	N	2
contig_6_383	-	N	CBM50_e1	CBM50	N	2
contig_1_4	N	N	N	CBM13	N	1
contig_2_30	N	N	N	GH13_3	N	1
contig_3_4	N	N	N	GT2	N	1
contig_3_126	N	N	N	GH24	N	1
contig_3_146	N	N	N	GH1	N	1
contig_3_276	N	N	N	GT30	N	1
contig_3_282	N	N	N	GH101	N	1
contig_3_394	N	N	N	GT0	N	1
contig_3_395	N	N	N	GT2	N	1
contig_3_396	N	N	N	GT2	N	1
contig_3_397	N	N	N	GT2	N	1
contig_3_398	N	N	N	GT2	N	1
contig_3_436	N	N	N	GT2	N	1
contig_3_448	N	N	N	GH13_48	N	1
contig_3_454	N	N	N	GT2	N	1
contig_3_460	N	N	N	CBM50+GH25	N	1
contig_3_551	N	N	N	CBM50	N	1
contig_3_573	N	N	N	GT0	N	1
contig_4_26	N	N	N	GT0	N	1
contig_4_28	N	N	N	GT0	N	1
contig_4_32	N	N	N	GT4	N	1
contig_4_51	N	N	N	CE12	N	1
contig_4_59	N	N	N	GH105	N	1
contig_4_106	N	N	N	GH13_23	N	1
contig_4_125	N	N	N	GH131	N	1
contig_4_145	N	N	N	GH13	N	1
contig_5_52	N	N	N	GH0	N	1
contig_5_53	N	N	N	GH4	N	1
contig_5_89	N	N	N	CBM50	N	1
contig_5_93	N	N	N	GH13_31	N	1
contig_6_1	N	N	N	GT1	N	1
contig_6_2	N	N	N	GT2	N	1
contig_6_7	N	N	N	GT32	N	1
contig_6_192	N	N	N	GT1	N	1
contig_6_196	N	N	N	GH28	N	1
contig_6_274	N	N	N	GH13_3	N	1
contig_6_282	N	N	N	GT4	N	1
contig_6_380	N	N	N	GH5_11	N	1
contig_6_392	N	N	N	GT2	N	1
contig_6_398	N	N	N	GT2	N	1
contig_7_26	N	N	N	GT2	N	1
contig_8_48	N	N	N	GH0	N	1
contig_8_73	N	N	N	GT0	N	1
contig_8_103	N	N	N	GH28	N	1
contig_8_153	N	N	N	GT4	N	1
contig_8_157	N	N	N	GH23	N	1
contig_8_168	N	N	N	GH13_11	N	1
contig_8_177	N	N	N	GT58	N	1
contig_8_241	N	N	N	GT2	N	1
contig_10_48	N	N	N	CE4	N	1
contig_10_67	N	N	N	GH0	N	1
contig_11_46	N	N	N	CE4	N	1
contig_11_50	N	N	N	GH1	N	1
contig_11_71	N	N	N	GT4	N	1
contig_12_100	N	N	N	CBM6	N	1
contig_12_196	N	N	N	GT1	N	1
contig_13_12	N	N	N	GH130_1	N	1
contig_14_15	N	N	N	GH9	N	1
contig_14_54	N	N	N	CBM50	N	1
contig_14_76	N	N	N	GT22	N	1
contig_14_102	N	N	N	GT30	N	1
contig_15_59	N	N	N	CBM12	N	1
contig_15_107	N	N	N	CBM50	N	1
contig_16_2	N	N	N	GT2	N	1
contig_16_45	N	N	N	GH36	N	1
contig_16_84	N	N	N	GH126	N	1
contig_17_30	N	N	N	GH28	N	1
contig_17_59	N	N	N	GH13_30	N	1
contig_17_119	N	N	N	GT2	N	1
contig_17_126	N	N	N	GT4	N	1
contig_17_152	N	N	N	CBM91+GH43_11	N	1
contig_17_165	N	N	N	GH4	N	1
contig_17_168	N	N	N	GT4	N	1
contig_17_190	N	N	N	GH13_3	N	1
contig_17_225	N	N	N	CBM48+GH13_9	N	1
contig_18_2	N	N	N	CBM50	N	1
contig_18_6	N	N	N	GH1	N	1
contig_18_37	N	N	N	GH38	N	1
contig_19_11	N	N	N	GH13_31	N	1
contig_19_59	N	N	N	CBM50	N	1
contig_19_87	N	N	N	CE8	N	1
contig_21_32	N	N	N	GT2	N	1
contig_21_41	N	N	N	GT0	N	1
contig_21_44	N	N	N	GT4	N	1
contig_21_52	N	N	N	GT2	N	1
contig_23_17	N	N	N	GT1	N	1
contig_27_12	N	N	N	GT4	N	1
contig_34_1	N	N	N	GT119	N	1
contig_34_14	N	N	N	GT50	N	1
contig_34_45	N	N	N	GH84	N	1
contig_36_2	N	N	N	GT1	N	1
contig_37_26	N	N	N	GH92	N	1
contig_42_1	N	N	N	GT1	N	1
contig_42_2	N	N	N	GT1	N	1
contig_43_1	N	N	N	GT1	N	1
contig_43_2	N	N	N	GT1	N	1

N67B2_overview

Gene ID	EC#	HMMER	dbCAN_sub	DIAMOND	Signalp	#ofTools
**contig_10_12**	3.5.1.104	CE4(272-383)	CE4_e217	CE4	N	3
**contig_10_126**	-	GT2(7-127)	GT2	GT2	N	3
**contig_10_148**	-	GT28(198-342)	GT28_e9	GT28	N	3
**contig_10_152**	-	GT2(9-179)	GT2	GT2	N	3
**contig_10_192**	-	GH18(31-301)	GH18_e71	GH18	N	3
**contig_10_24**	-	GT1(212-386)	GT1_e509	GT1	N	3
**contig_10_76**	-	CBM50(161-218)	CBM50_e932	CBM50	N	3
**contig_10_89**	-	CBM50(161-203)	CBM50_e826	CBM50	N	3
**contig_10_96**	-	GT83(5-263)	GT83_e35	N	N	2
**contig_10_97**	-	GT2(8-170)	GT2	GT2	N	3
**contig_13_1**	-	CE4(50-179)	CE4_e14	CE4	N	3
**contig_14_63**	N	AA1(48-499)	N	N	N	1
**contig_15_9**	3.2.1.-	GH18(127-405)	CBM50_e699+CBM50_e699+GH18_e157	CBM50+GH18	N	3
**contig_1_1**	2.4.1.18	CBM48(26-109)+GH13_9(178-468)	CBM48_e2+GH13_e200	CBM48+GH13_9	N	3
**contig_1_197**	3.2.1.99	GH43_5(43-321)	GH43_e56	GH43_5	N	3
**contig_1_206**	3.2.1.55	GH51_1(6-496)	GH51_e0	GH51_1	N	3
**contig_1_213**	-	AA4(8-239)	AA4_e1	N	N	2
**contig_1_230**	3.2.1.55	GH51_1(7-490)	GH51_e19	GH51_1	N	3
**contig_1_298**	-	CBM50(4-48)	CBM50_e257	CBM50	N	3
**contig_1_305**	N	GH188(8-251)	N	N	N	1
**contig_1_385**	3.2.1.65|3.2.1.80|3.2.1.26|3.2.1.153|3.2.1.64|2.4.1.99	GH32(39-349)+CBM66(520-673)	GH32_e16+CBM66_e0	CBM66+GH32	N	3
**contig_1_4**	2.4.1.21	GT5(2-473)	GT5_e9	GT5	N	3
**contig_1_5**	2.4.1.1	GT35(93-795)	GT35_e0	GT35	N	3
**contig_1_61**	3.2.1.22	GH4(3-181)	GH4_e17	GH4	N	3
**contig_1_66**	-	CE19(14-297)	CE19_e3	N	N	2
**contig_1_78**	3.2.1.172	GH105(30-362)	GH105_e52	GH105	N	3
**contig_1_80**	N	GH188(27-212)	N	N	N	1
**contig_1_96**	3.2.1.41	CBM68(5-97)+GH13_14(257-570)	CBM68_e0+CBM48_e46+GH13_e94	CBM48+CBM68+GH13_14	N	3
**contig_20_2**	N	GT119(19-372)	N	GT119	N	2
**contig_23_14**	-	GT2(5-170)	GT2	GT2	N	3
**contig_30_1**	3.2.1.132	GH46(1-179)	GH46_e0	N	N	2
**contig_3_139**	N	GH177(5-304)	N	N	N	1
**contig_3_172**	-	GH188(3-206)	GH109_e7	N	N	2
**contig_3_214**	2.4.1.129	GT51(56-230)	GT51_e20	GT51	N	3
**contig_3_256**	-	CE4(80-207)	CE4_e25	CE4	N	3
**contig_3_30**	N	CE1(15-237)	N	N	N	1
**contig_3_319**	-	AA6(2-169)	AA6_e2	N	N	2
**contig_3_60**	3.2.1.17|4.2.2.n1	GH23(138-241)	GH23_e335	N	N	2
**contig_4_339**	-	CE14(7-112)	CE14_e0	CE14	N	3
**contig_4_340**	2.4.1.-	GT4(196-344)	GT4_e1442	GT4	N	3
**contig_4_354**	2.4.1.129	GT51(84-260)	GT51_e113	GT51	N	3
**contig_4_395**	2.4.1.157	GT28(205-353)	GT28_e89	GT28	N	3
**contig_4_425**	-	GT2(6-168)	GT2	GT2	N	3
**contig_4_464**	-	CE14(6-124)	CE14_e39	N	N	2
**contig_4_469**	-	GT1(201-396)	GT1_e505	GT1	N	3
**contig_4_518**	1.14.99.53	AA10(28-203)	AA10_e1	AA10	N	3
**contig_4_522**	3.2.1.37|3.2.1.55|3.2.1.-|-	GH43_11(3-301)+CBM91(331-532)	GH43_e285+CBM91_e24	CBM91+GH43_11	N	3
**contig_4_539**	4.2.2.10	PL1_8(83-276)	PL1_e25	PL1_8	N	3
**contig_4_541**	-	CBM63(139-214)	CBM63_e7	CBM63	N	3
**contig_4_7**	N	CE12(25-196)	N	N	N	1
**contig_4_88**	-	GT4(177-325)	GT4_e992	GT4	N	3
**contig_5_1018**	5.4.99.11|3.2.1.20|3.2.1.10|3.2.1.-|3.2.1.70	GH13_31(28-372)	GH13_e122	GH13_31	N	3
**contig_5_1032**	N	GH188(2-144)	N	N	N	1
**contig_5_1039**	3.2.1.-	GH73(59-194)	GH73_e25	GH73	N	3
**contig_5_140**	N	GH188(3-160)	N	N	N	1
**contig_5_176**	3.2.1.99	GH43_4(34-360)	GH43_e38	GH43_4	N	3
**contig_5_186**	3.2.1.86|3.2.1.21|3.2.1.37	GH1(6-466)	GH1_e85	GH1	N	3
**contig_5_189**	N	CBM16(1290-1426)	N	CBM0+CBM16	N	2
**contig_5_203**	3.1.1.-	CE12(179-374)	CE12_e21	CE12	N	3
**contig_5_209**	3.2.1.21|3.2.1.-|3.2.1.23	GH1(8-468)	GH1_e87	GH1	N	3
**contig_5_237**	-	CE4(114-242)	CE4_e274	N	N	2
**contig_5_261**	3.2.1.86	GH4(6-185)	GH4_e24	GH4	N	3
**contig_5_277**	-	GT8(5-258)	GT8_e207	GT8	N	3
**contig_5_311**	N	GT119(19-381)	N	GT119	N	2
**contig_5_319**	3.2.1.26	GH32(33-338)	GH32_e57	GH32	N	3
**contig_5_325**	-	GT2(4-163)	GT2	GT2	N	3
**contig_5_332**	-	GT2(5-167)	GT2	GT2	N	3
**contig_5_382**	-	GT51(62-238)	GT51_e8	GT51	N	3
**contig_5_490**	-	PL9_2(33-390)	PL9_e18	PL9_2	N	3
**contig_5_558**	3.2.1.-|3.2.1.17	GH73(735-862)	GH73_e162	GH73	N	3
**contig_5_561**	2.4.1.-	GT26(57-224)	GT26_e19	GT26	N	3
**contig_5_563**	-	GT4(345-496)	GT4_e2213	GT4	N	3
**contig_5_570**	-	GT4(211-361)	GT4_e633	GT4	N	3
**contig_5_571**	-	GT4(330-480)	GT4_e3978	GT4	N	3
**contig_5_580**	-	GT4(218-351)	GT4_e23	GT4	N	3
**contig_5_584**	-	GT2(9-114)	GT2	GT2	N	3
**contig_5_59**	-	GT83(9-225)	GT83_e5	N	N	2
**contig_5_635**	3.5.1.25	CE9(9-384)	CE9_e51	CE9	N	3
**contig_5_641**	4.2.2.2	PL3_1(28-204)	PL3_e8	PL3_1	N	3
**contig_5_676**	3.2.1.54|3.2.1.133|3.2.1.135	CBM34(6-126)+GH13_20(175-468)	CBM34_e0+GH13_e225	CBM34+GH13_20	N	3
**contig_5_681**	2.4.1.8	GH65(319-689)	GH65_e0	GH65	N	3
**contig_5_682**	5.4.99.11|3.2.1.20|3.2.1.10|3.2.1.-|3.2.1.70	GH13_31(27-377)	GH13_e122	GH13_31	N	3
**contig_5_691**	3.2.1.65|3.2.1.64	GH32(48-356)	GH32_e117	GH32	N	3
**contig_5_692**	2.4.1.10	GH68_1(41-469)	GH68_e0	GH68_1	N	3
**contig_5_707**	-	GT4(190-343)	GT4_e2316	GT4	N	3
**contig_5_708**	-	GT2(8-160)	GT2	GT2	N	3
**contig_5_709**	-	GT4(203-309)	GT4_e1080	GT4	N	3
**contig_5_710**	N	GT122(39-352)	N	GT122	N	2
**contig_5_711**	-	GT2(7-169)	GT2	GT2	N	3
**contig_5_713**	-	GT2(6-148)	GT2	GT2	N	3
**contig_5_727**	3.2.1.23	GH42(20-399)	GH42_e20	GH42	N	3
**contig_5_728**	3.2.1.89	GH53(54-417)	GH53_e14	GH53	N	3
**contig_5_735**	-	GH18(33-331)	GH18_e163	GH18	N	3
**contig_5_755**	-	CE14(4-114)	CE14_e46	N	N	2
**contig_5_792**	N	GH179(33-197)	N	N	N	1
**contig_5_946**	N	CE1(34-265)	N	N	N	1
**contig_5_996**	-	GT51(63-239)	GT51_e55	GT51	N	3
**contig_6_225**	-	GH18(172-401)	CBM50_e96+CBM50_e96+GH18_e174	CBM50+GH18	N	3
**contig_6_226**	-	GT1(28-389)	GT1_e274	GT1	N	3
**contig_6_236**	3.2.1.86|3.2.1.85|3.2.1.21	GH1(7-464)	GH1_e13	GH1	N	3
**contig_6_240**	3.2.1.78|3.2.1.-	GH26(32-350)	GH26_e0	GH26	N	3
**contig_6_85**	-	GH126(45-353)	GH126_e0	GH126	N	3
**contig_6_88**	-	GT2(49-279)	GT2	GT2	N	3
**contig_7_104**	3.2.1.93	GH13_29(30-377)	GH13_e1	GH13_29	N	3
**contig_7_121**	3.5.1.104	CE4(59-185)	CE4_e23	CE4	N	3
**contig_7_144**	3.2.1.122	GH4(8-185)	GH4_e4	GH4	N	3
**contig_7_184**	-	GT2(7-168)	GT2	GT2	N	3
**contig_7_27**	3.2.1.172	GH105(25-337)	GH105_e0	GH105	N	3
**contig_7_29**	3.1.1.72	CE12(6-211)	CE12_e45	CE12	N	3
**contig_7_32**	4.2.2.23|4.2.2.24	PL11(33-613)	PL11_e0	PL11_1	N	3
**contig_7_33**	4.2.2.23|4.2.2.24	PL11(1-601)	PL11_e0	PL11_1	N	3
**contig_7_35**	3.1.1.-	CE12(5-201)	CE12_e0	CE12	N	3
**contig_7_36**	3.2.1.23	GH42(10-371)	GH42_e10	GH42	N	3
**contig_7_37**	4.2.2.24	PL26(3-845)	PL26_e0	PL26	N	3
**contig_7_41**	3.2.1.67	GH4(11-186)	GH4_e30	GH4	N	3
**contig_7_57**	-	GT2(4-125)	GT2	GT2	N	3
**contig_7_80**	4.2.2.2	PL1_6(135-347)	PL1_e108	PL1_6	N	3
**contig_8_186**	-	CE4(123-245)	CE4_e20	CE4	N	3
**contig_8_255**	3.2.1.1|3.2.1.98|3.2.1.41	GH13_5(62-402)	GH13_e5	GH13_5	N	3
**contig_8_297**	3.2.1.73|3.2.1.6|3.2.1.8	GH16_21(37-238)	GH16_e14	GH16_21	N	3
**contig_8_307**	3.2.1.8	GH11(32-211)	GH11_e15	GH11	N	3
**contig_8_366**	3.2.1.4|3.2.1.78|3.2.1.132	GH5_2(52-290)+CBM3(357-437)	GH5_e251+CBM3_e18	CBM3+GH5_2	N	3
**contig_8_369**	-	CE6(55-146)	CE6_e1	CE0	N	3
**contig_8_37**	N	GT119(2-337)	N	GT119	N	2
**contig_8_370**	3.2.1.8|3.2.1.136	GH30_8(34-420)	GH30_e78	GH30_8	N	3
**contig_8_371**	3.2.1.55|3.2.1.8|3.2.1.-|3.2.1.37	GH43_16(38-361)+CBM6(382-511)	GH43_e261+CBM6_e6	CBM6+GH43_16	N	3
**contig_8_38**	-	GT28(188-350)	GT28_e1	GT28	N	3
**contig_9_117**	5.4.99.11|3.2.1.20|3.2.1.10|3.2.1.-|3.2.1.70	GH13_31(28-375)	GH13_e122	GH13_31	N	3
**contig_9_141**	3.2.1.1	GH13_28(58-339)+CBM26(564-642)	GH13_e34+CBM26_e0	CBM26+GH13_28	N	3
**contig_9_155**	3.1.1.41|3.1.1.72	CE7(3-312)	CE7_e11	CE7	N	3
**contig_9_182**	3.2.1.86|3.2.1.38|3.2.1.85|3.2.1.21|3.2.1.-	GH1(7-474)	GH1_e115	GH1	N	3
**contig_9_3**	N	CE1(2-228)	N	N	N	1
**contig_9_8**	3.2.1.92	GH171(57-414)	GH171_e0	GH171	N	3
**contig_9_9**	3.2.1.52	GH3(112-348)	GH3_e28	GH3	N	3
**contig_10_49**	-	N	CBM50_e826	CBM50	N	2
**contig_1_270**	-	N	CBM50_e1	CBM50	N	2
**contig_22_31**	-	N	CBM50_e826	CBM50	N	2
**contig_3_281**	-	N	CBM50_e1004+CBM50_e1004+CBM50_e	CBM50	N	2
**contig_4_284**	-	N	CBM50_e1023	N	N	1
**contig_4_470**	-	N	CBM50_e1004+CBM50_e1004+CBM50_e	CBM50	N	2
**contig_4_486**	3.2.1.96|3.2.1.-|3.2.1.17|-	N	CBM50_e375+CBM50_e259	CBM50	N	2
**contig_4_496**	-	N	GH23_e590	GH23	N	2
**contig_5_585**	-	N	GT4_e101	GT4	N	2
**contig_7_82**	-	N	GT2	N	N	1
**contig_10_191**	-	N	CBM50_e1022	CBM50	N	2
**contig_10_226**	-	N	CBM50_e55	N	N	1
**contig_10_75**	-	N	GH23_e126	GH0	N	2
**contig_3_286**	-	N	CBM50_e833+CBM50_e1004+CBM50_e1	CBM50	N	2
**contig_1_2**	N	N	N	CBM48+GH13_9	N	1
**contig_1_37**	N	N	N	GH13_3	N	1
**contig_1_59**	N	N	N	GT4	N	1
**contig_1_62**	N	N	N	GH4	N	1
**contig_1_75**	N	N	N	CBM91+GH43_11	N	1
**contig_1_101**	N	N	N	GT4	N	1
**contig_1_108**	N	N	N	GT2	N	1
**contig_1_155**	N	N	N	GH13_30	N	1
**contig_1_184**	N	N	N	GH28	N	1
**contig_1_255**	N	N	N	GT2	N	1
**contig_1_261**	N	N	N	GT2	N	1
**contig_1_273**	N	N	N	GH5_11	N	1
**contig_1_371**	N	N	N	GT4	N	1
**contig_1_379**	N	N	N	GH13_3	N	1
**contig_2_14**	N	N	N	GH13	N	1
**contig_3_64**	N	N	N	CBM50	N	1
**contig_3_114**	N	N	N	CBM12	N	1
**contig_3_292**	N	N	N	GH130_1	N	1
**contig_3_305**	N	N	N	CBM50	N	1
**contig_3_309**	N	N	N	GH1	N	1
**contig_3_340**	N	N	N	GH38	N	1
**contig_4_27**	N	N	N	GH28	N	1
**contig_4_31**	N	N	N	GT1	N	1
**contig_4_191**	N	N	N	GH101	N	1
**contig_4_197**	N	N	N	GT30	N	1
**contig_4_326**	N	N	N	GH1	N	1
**contig_4_346**	N	N	N	GH24	N	1
**contig_4_474**	N	N	N	GT2	N	1
**contig_4_480**	N	N	N	GH92	N	1
**contig_5_33**	N	N	N	GH9	N	1
**contig_5_52**	N	N	N	CBM50	N	1
**contig_5_74**	N	N	N	GT22	N	1
**contig_5_314**	N	N	N	GH0	N	1
**contig_5_338**	N	N	N	GT0	N	1
**contig_5_369**	N	N	N	GH28	N	1
**contig_5_427**	N	N	N	GT4	N	1
**contig_5_431**	N	N	N	GH23	N	1
**contig_5_442**	N	N	N	GH13_11	N	1
**contig_5_451**	N	N	N	GT58	N	1
**contig_5_513**	N	N	N	GT2	N	1
**contig_5_564**	N	N	N	GT2	N	1
**contig_5_573**	N	N	N	GT0	N	1
**contig_5_581**	N	N	N	GT2	N	1
**contig_5_677**	N	N	N	CBM50+CE4	N	1
**contig_5_678**	N	N	N	GH13_48	N	1
**contig_5_714**	N	N	N	GT2	N	1
**contig_5_747**	N	N	N	GH13_31	N	1
**contig_5_795**	N	N	N	CBM50	N	1
**contig_5_822**	N	N	N	CE8	N	1
**contig_5_897**	N	N	N	CE4	N	1
**contig_5_916**	N	N	N	GH0	N	1
**contig_5_951**	N	N	N	GT1	N	1
**contig_6_1**	N	N	N	GT1	N	1
**contig_6_2**	N	N	N	GT2	N	1
**contig_6_45**	N	N	N	GH36	N	1
**contig_6_86**	N	N	N	GH126	N	1
**contig_6_213**	N	N	N	GH13_3	N	1
**contig_7_2**	N	N	N	GT2	N	1
**contig_7_26**	N	N	N	GH105	N	1
**contig_7_34**	N	N	N	CE12	N	1
**contig_7_52**	N	N	N	GT4	N	1
**contig_7_56**	N	N	N	GT0	N	1
**contig_7_58**	N	N	N	GT0	N	1
**contig_7_103**	N	N	N	GH13_31	N	1
**contig_7_107**	N	N	N	CBM50	N	1
**contig_7_146**	N	N	N	GH4	N	1
**contig_8_60**	N	N	N	GT0	N	1
**contig_8_82**	N	N	N	CBM50	N	1
**contig_8_173**	N	N	N	CBM50+GH25	N	1
**contig_8_179**	N	N	N	GT2	N	1
**contig_8_185**	N	N	N	GH13_48	N	1
**contig_8_197**	N	N	N	GT2	N	1
**contig_8_238**	N	N	N	GT2	N	1
**contig_8_239**	N	N	N	GT2	N	1
**contig_8_240**	N	N	N	GT2	N	1
**contig_8_241**	N	N	N	GT2	N	1
**contig_8_242**	N	N	N	GT0	N	1
**contig_8_318**	N	N	N	GH0	N	1
**contig_8_381**	N	N	N	GT32	N	1
**contig_8_386**	N	N	N	GT2	N	1
**contig_8_387**	N	N	N	GT1	N	1
**contig_8_388**	N	N	N	GT1	N	1
**contig_9_53**	N	N	N	GH0	N	1
**contig_9_95**	N	N	N	CBM6	N	1
**contig_9_189**	N	N	N	GT1	N	1
**contig_10_185**	N	N	N	GT4	N	1
**contig_11_1**	N	N	N	GT119	N	1
**contig_11_14**	N	N	N	GT50	N	1
**contig_11_45**	N	N	N	GH84	N	1
**contig_13_46**	N	N	N	CE4	N	1
**contig_13_50**	N	N	N	GH1	N	1
**contig_13_71**	N	N	N	GT4	N	1
**contig_14_1**	N	N	N	GT0	N	1
**contig_14_5**	N	N	N	GT2	N	1
**contig_14_39**	N	N	N	GH13_23	N	1
**contig_14_57**	N	N	N	GH131	N	1
**contig_15_4**	N	N	N	CBM13	N	1
**contig_19_1**	N	N	N	GT1	N	1
**contig_21_2**	N	N	N	GT1	N	1
**contig_21_3**	N	N	N	GT2	N	1
**contig_24_2**	N	N	N	GT2	N	1
