# Supplementary material for: Genomic and functional characterization of Bacillus strains active against Fusarium graminearum
Source: Front Microbiol. 2026 May 29;17:1832933. doi: 10.3389/fmicb.2026.1832933 (PMC13260424; doi:10.3389/fmicb.2026.1832933)
Supplement: Supplementary file 2 [file Data_sheet_1.pdf]

Supplementary Material

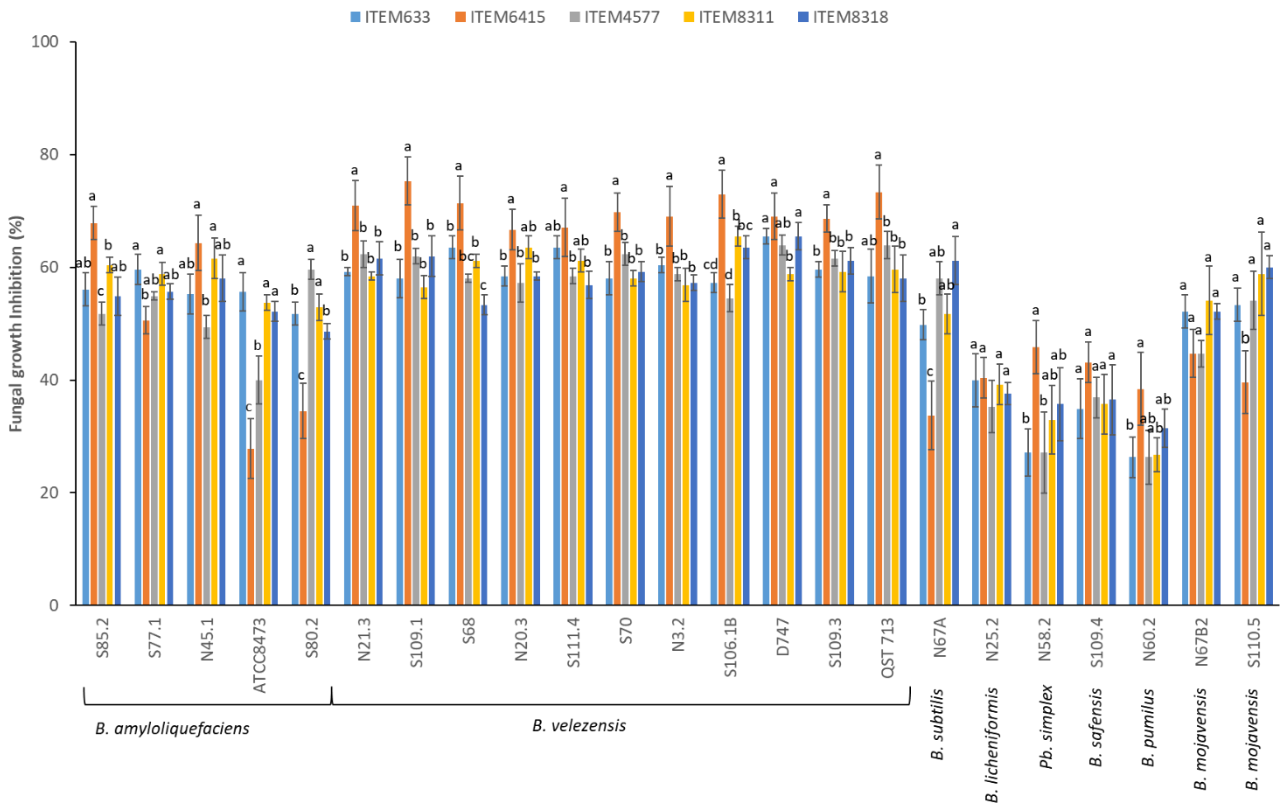

Supplementary Figure 1. Antagonistic activity of *Bacillus* strains against five *F. graminearum* strains (ITEM 633, ITEM 4577, ITEM 6415, ITEM 8311 and ITEM 8318) on TSA, after 7 days of incubation at 25 °C. Different letters indicate statistically different antagonistic activity of each *Bacillus* strain against five fungal strains ($P<0.05$), determined by one-way analysis of variance (ANOVA) followed by the Tukey test.

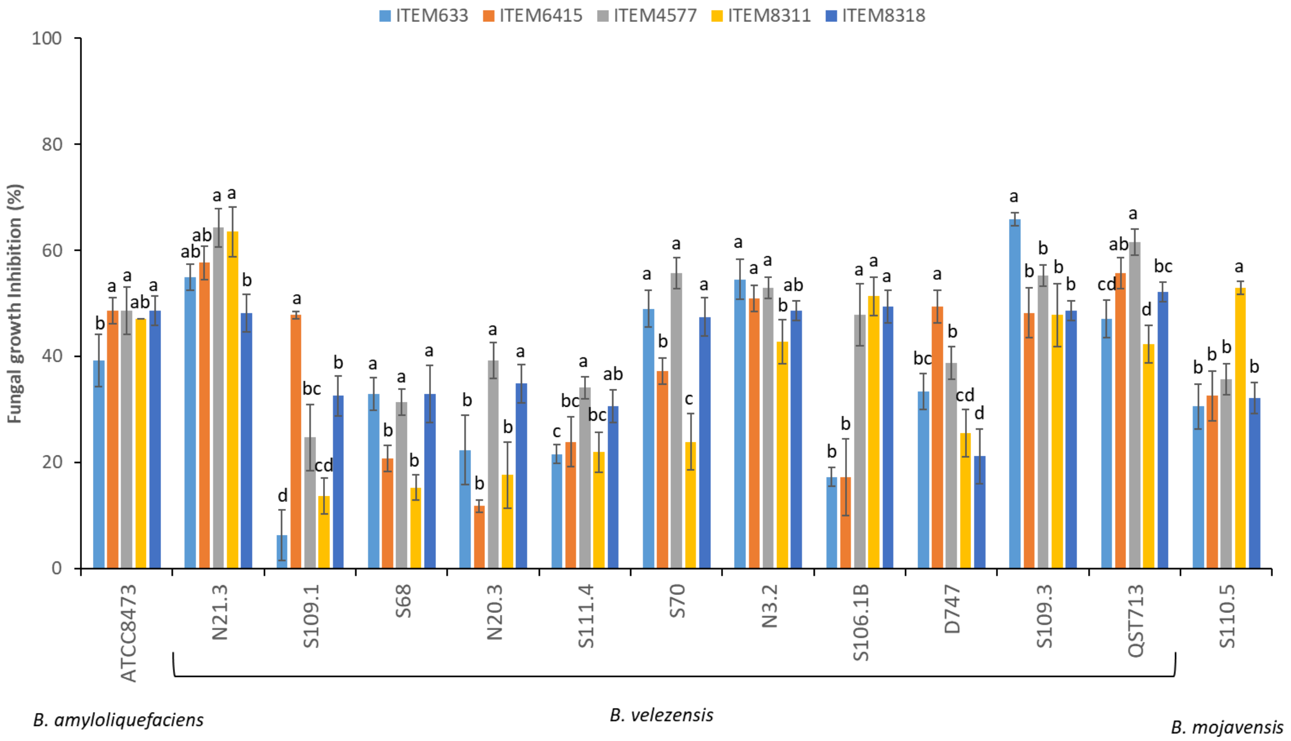

Supplementary Figure 2. Antifungal activity of *Bacillus* culture filtrates against five *F. graminearum* strains (ITEM 633, ITEM 4577, ITEM 6415, ITEM 8311 and ITEM 8318) on PDA, after 7 days of incubation at 25 °C. The fungal growth inhibition of each *F. graminearum* strain was expressed as percentage. Different letters indicate statistically different antifungal activity of each *Bacillus* strain against five fungal strains (P<0.05), determined by one-way analysis of variance (ANOVA) followed by the Tukey test.

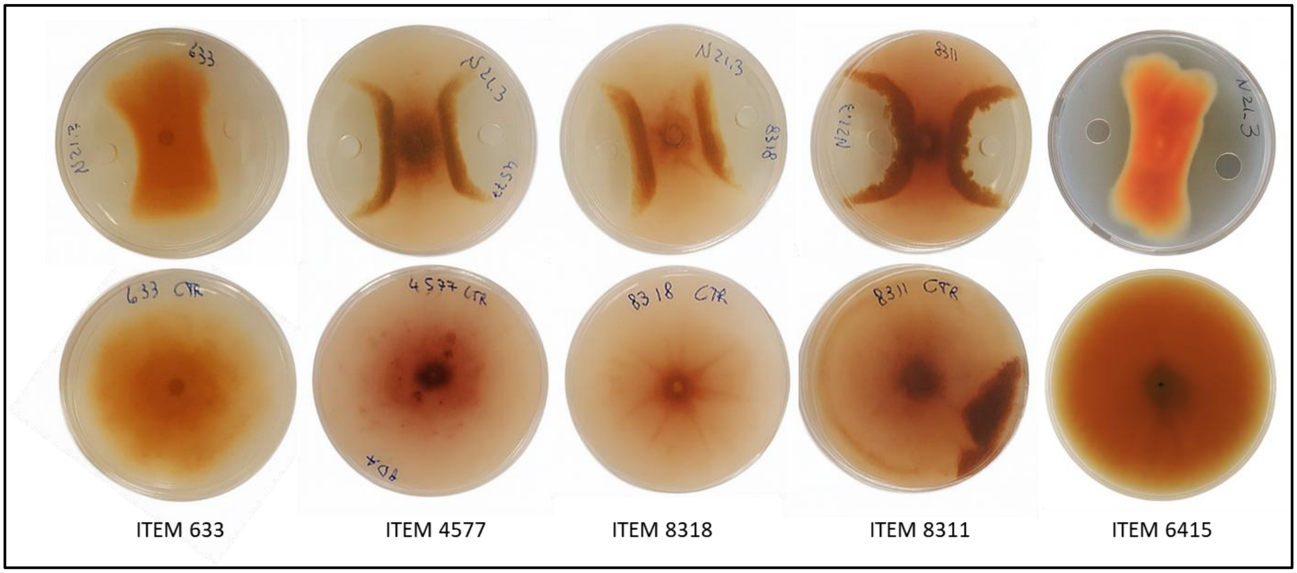

Supplementary Figure 3. Antifungal activity of *Bacillus velezensis* N21.3 culture filtrates against five *F. graminearum* strains (ITEM 633, ITEM 4577, ITEM 8318, ITEM 8311 and ITEM 6415). The activity was assessed on PDA, after 7 days of incubation at 25 °C.

Supplementary Figure 4.

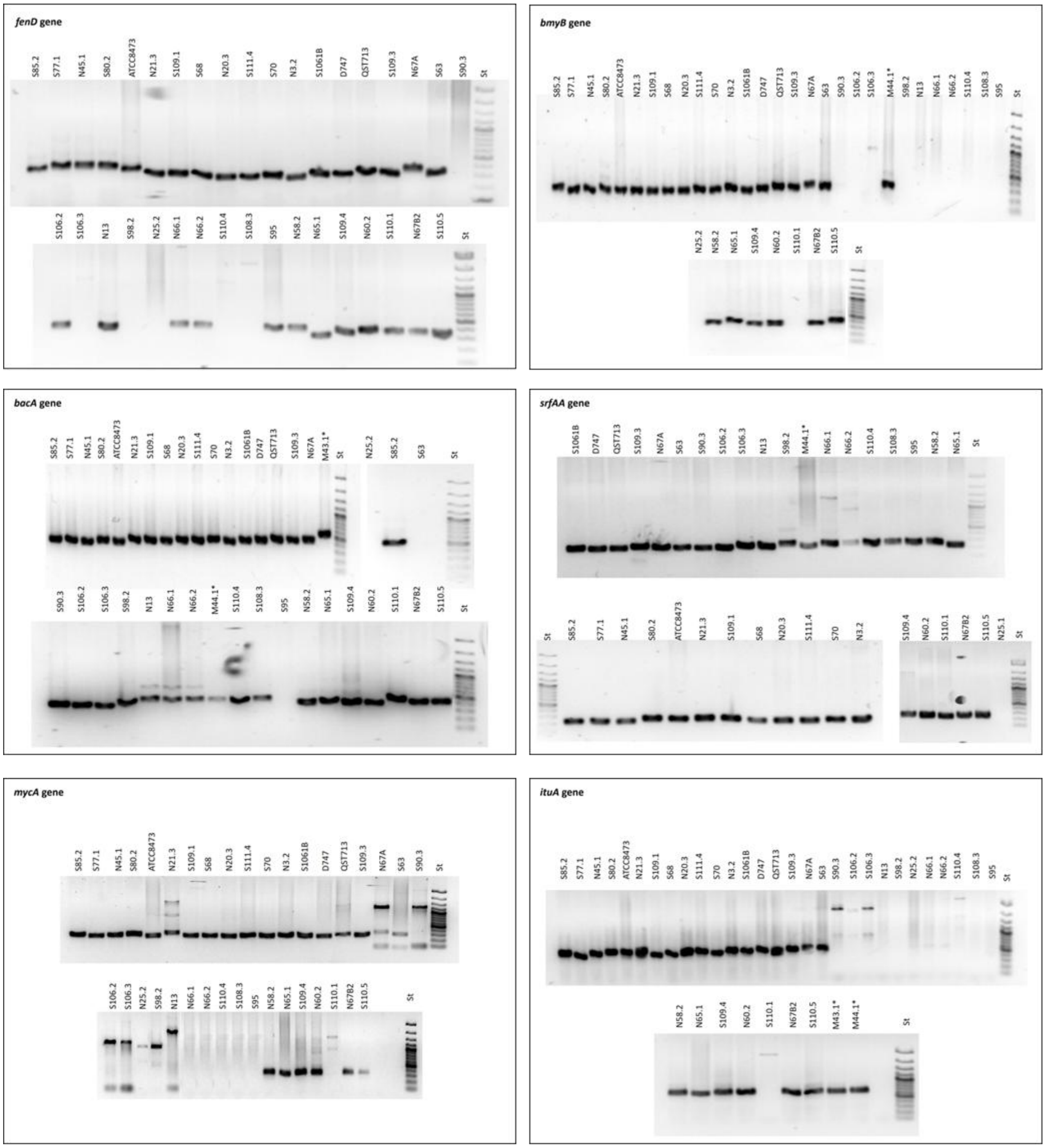

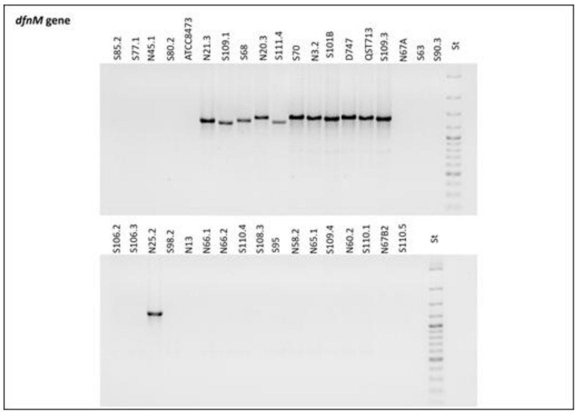

Supplementary Figure 4. Amplicons of fengycin (*fenD*), bacillomycin (*bmyB*), bacilysin (*bacA*), surfactin (*srfAA*), mycosubtilin (*mycA*), iturin (*ituA*), and difficidin (*dfnM*) genes of *Bacillus* strains used in this study.
* Strains not reported in this study
St = 100 bp DNA ladder (New England Bio-Labs, Beverly, MA).

Supplementary Table 1. Genome assembly metrics

Species	Strain	Reads	Count	Total	N75	N50	N25	Minimum	Maximum	Average
B. amyloliquefaciens	S77.1	8.176.614	93	3.944.324	208.822	502.626	710.662	203	875.935	42.412
	N45.1	9.677.346	78	4.010.911	208.884	527.322	716.436	153	876.131	51.422
	ATCC8473	23.983.216	481	4.145.047	208.933	527.324	716.445	200	876.188	8.618
B. velezensis	N21.3	9.378.436	121	4.221.698	159.578	249.822	355.860	196	618.985	34.890
	N20.3	8.195.468	66	3.952.289	577.869	1.006.667	1.446.172	200	1.446.172	59.883
	N3.2	7.413.796	111	4.263.107	115.021	158.392	337.885	201	412.796	38.406
	S106.1B	10.896.996	138	3.987.144	264.636	349.343	1.014.601	198	1.014.601	28.892
	S111.4	15.599.612	145	3.984.438	274.670	604.788	943.222	200	944.258	27.479
B. mojavensis	N67B2	13.270.600	250	4.131.514	226.859	410.386	1.074.085	150	1.074.085	16.526
	S110.5	7.713.258	93	4.023.937	115.412	175.971	411.107	206	629.829	43.268

Reads: amount for all reads (length 150bp) sequenced; **Count:** the total number of assembled contigs; **Total:** the number of bases in the contigs (genome size estimation; **N25, N50, N75:** the N25 contig set is calculated by summarizing the lengths of the biggest contigs until you reach 25 % of the total contig length. The minimum contig length in this set is the number that is usually used to report the N25 value of a de novo assembly. The same goes with N50 and N75 which are the 50 % and 75 % of the total contig length, respectively; **Minimum, Maximum, Average:** this refers to the contig lengths

Supplementary Table 2. Unknown gene clusters detected in the genomes of 10 *Bacillus* strains selected for whole-genome sequencing.

**Type**	***B. velezensis***					***B. amyloliquefaciens***			***B. mojavensis***	
	**N21.3**	**S106.1b**	**N3.2**	**N20.3**	**S111.4**	**S77.1**	**N45.1**	**ATCC8473**	**N67B2**	**S110.5**
Ladderane		**1** (28.790)								
Lanthipeptide-class-iii						**1** (22.598)	**1** (22.598)	**1** (22.598)		
LAP.thiopeptide			**1** (22.000)							
NRPS	**6** (3.190 - 6.688 - 6.540 - 6.690 - 1.180 - 1.171)				**2** (40.296 - 21.455)	**1** (45.528)	**1** (45.528)	**1** (45.528)		
NRPS-like		**1** (1.751)				**1** (1.065)	**1** (1.218)	**1** (1.223)		
Phosphonate RRE			**1** (39.912)	**1** (40.885)	**1** (40.885)					
T3PKS	**1** (41.101)	**1** (41.101)	**1** (41.101)	**1** (39.649)	**1** (41.101)	**1** (41.101)	**1** (41.101)	**1** (41.101)	**1** (41.098)	**1** (40.943)
Terpene	**2** (20.741 - 21.884)	**2** (20.741 - 21.884)	**2** (20.741 - 21.884)	**2** (20.741 - 20.129)	**2** (20.741 - 21.884)	**2** (20.741 - 21.884)	**2** (20.741 - 21.884)	**2** (20.741 - 21.884)	**2** (20.741 - 21.899)	**2** (20.807 - 12.766)

The number of gene clusters is in bold; the size of each cluster, expressed as the number of nucleotides is reported in brackets. The description of different types of secondary metabolite clusters is available at https://docs.antismash.secondarymetabolites.org/glossary/

Supplementary Table 4. *Bacillus* genomes were screened for virulence factor (VF) genes, plasmid replicons (PlasmidFinder), and antimicrobial resistance genes (ARGs) using ABRicate and abriTAMR. ARGs are reported as predicted resistance determinants; * indicates a high-confidence match, while ^ denotes a putative homologue with partial sequence divergence. Associated resistance profiles are: satA (streptothricin), clbA (lincosamide), blaBSU-1 (beta-lactam), tet(L) (tetracyclines), aadK (streptomycin), mph(K) (macrolides), and cfr(B) (PhLOPSA phenotype).

Classification	Strain	VF gene prediction/Total genes	Plasmid	ABRicate				abriTAMR			
B. velezensis	N21.3	39/4097	absent	tet(L)	cfr(B)				satA*	clbA*	
B. velezensis	N20.3	41/3925	absent	tet(L)					satA*		
B. velezensis	N3.2	39/4637	absent	tet(L)					satA*		
B. velezensis	S106.1B	39/3995	absent	tet(L)					satA*		
B. velezensis	S111.4	38/3926	absent	tet(L)					satA*		
B. amyloliquefaciens	S77.1	41/3925	absent	tet(L)	cfr(B)				satA*	clbA*	
B. amyloliquefaciens	N45.1	36/4214	absent	tet(L)	cfr(B)				satA*		
B. amyloliquefaciens	ATCC8473	36/4326	absent	tet(L)	cfr(B)				satA*	clbA^	
B. mojavensis	N67B2	38/4140	absent			aadK	mph(K)	mphK*			
B. mojavensis	S110.5	38/4200	absent				mph(K)	mphK*			blaBSU-1*

Supplementary Table 5. Virulence factors prediction for *Bacillus* strains according VFanalyser tool (http://www.mgc.ac.cn/VFs/main.htm). For each *Bacillus* strain, number of genes putatively associated with virulence factors are reported.

			Bacillus strains									
VFclass	Virulence factors	Related genes	N21.3	N20.3	N3.2	S106.1B	S111.4	S77.1	N45.1	ATCC8473	N67.B2	S110.5
Adherence	Fibronectin-binding protein	*fbpA*	1	1	1	1	1	1	1	1	1	1
	Flagella	*fliP*	1	1	1	1	1	1		1	1	
	D-alanine-polyphosphoribitol ligase	*dltA*							1			
Immune evasion	Hyaluronic acid (HA) capsule	*hasC*	1	1	1	1	1				1	1
	Polyglutamic acid capsule	*capA*	1	1	1	1	1	1	1	1	1	1
		capB	1	1	1	1	1	1	1	1	1	1
		capC	1	1	1	1	1	1	1	1	1	1
		capD	1	1	1	1	1	1	1	1	1	1
	Polysaccharide capsule	--	12	11	12	11	11	11	11	11	11	12
	Capsule	*cps4I*		1		1	1					
	LOS	--	1	1		1		1	1	1	1	
Iron acquisition	Bacillibactin	*dhbA*	1	1	1	1	1	1	1	1	1	1
		dhbB	1	1	1	1	1	1	1	1	1	1
		dhbC	1	1	1	1	1	1	1	1	1	1
		dhbE	1	1	1	1	1	1	1	1	1	1
		dhbF	1	1	1	1	1	1	1	1	1	1
Regulation	Carbon storage regulator A	*csrA*										1
	CheA/CheY	*cheY*	2	2	2	2	2	2	2	2	1	1
	VirR/VirS	*virR*		1		1						
Secretion system	Type III secretion system	*cdsN*	1	1	1	1	1	1	1	1	1	1
Toxin	Hemolysin III	*hlyIII*	1	1	1	1	1	1	1	1	1	1
	Cytolysin	*cylR2*			1							
	Phytotoxin phaseolotoxin	*cysC1*	1	1	1		1					
Acid resistance	Urease	*ureB*	1	1	1	1	1	1	1	1	1	1
Antiphagocytosis	Capsular polysaccharide	*wcaJ*	1	1	1	1	1	1	1	1	1	1
	Capsule	--	1	1	1	1	1	1	1	1	2	2
Invasion	Flagella	*fliQ*									1	1
Cell surface components	GPL locus	*mbtH*	1	1	1	1	1	1	1	1		
	Trehalose-recycling ABC transporter	*sugC*	1	1	1	1	1	1	1	1		
Copper uptake	Copper exporter	*ctpV*		1								
Invasion	Flagella	*fliQ*	1	1	1	1	1	1	1	1		
Iron uptake												
	Achromobactin biosynthesis and transport	*cbrD*									1	1
	Mycobactin	*mbtH*									1	1
	Periplasmic binding protein-dependent ABC transport systems	*vctC*	1	1	1	1	1	1	1	1	1	1
Motility and export apparatus	Flagella	*fliP*										1
Stress adaptation	Catalase	*katA*	1	1	1	1	1	1	1	1	1	1
Surface protein anchoring	Lipoprotein-specific signal peptidase II	*lspA*	1	1	1	1	1	1	1	1	1	
	Lipoprotein diacylglyceryl transferase	*lgt*									1	1
